# Rhodocenium Functionalization Enabled by Half‐Sandwich Capping, Zincke Reaction, Diazoniation and Sandmeyer Chemistry

**DOI:** 10.1002/ejic.202100525

**Published:** 2021-07-16

**Authors:** Markus Wiedemair, Holger Kopacka, Klaus Wurst, Thomas Müller, Klaus Eichele, Stefan Vanicek, Stephan Hohloch, Benno Bildstein

**Affiliations:** ^1^ Institute of General, Inorganic and Theoretical Chemistry University of Innsbruck Center for Chemistry and Biomedicine Innrain 80–82 6020 Innsbruck Austria; ^2^ Institute of Organic Chemistry University of Innsbruck, Center for Chemistry and Biomedicine, Innrain 80–82 6020 Innsbruck Austria; ^3^ Institut für Anorganische Chemie Universität Tübingen Auf der Morgenstelle 18 72076 Tübingen Germany

**Keywords:** Metallocenes, Molecular electrochemistry, Rhodium, Sandwich complexes, Structure elucidation

## Abstract

In continuation of our exploration of metallocenium chemistry we report here on innovative ways toward monofunctionalized rhodocenium salts applying half‐sandwich capping reactions of cyclopentadienyl rhodium(III) halide synthons with cyclopentadienyl ylides containing pyridine, phosphine or dinitrogen leaving groups, followed by Zincke and Sandmeyer reactions. Thereby amino, diazonio, bromo, azido and iodo rhodocenium salts containing valuable functional groups are accessible for the first time. Target compounds were characterized by spectroscopic (^1^H/^13^C/^103^Rh‐NMR, IR, HR‐MS), structural (single crystal XRD) and electrochemical (CV) methods and their properties were compared to those of isoelectronic cobaltocenium compounds. These new functionalized rhodocenium complexes significantly expand the so far extremely limited chemical space of rhodocenium salts with promising options for the future development in the area of rhodocenium chemistry.

## Graphical abstract

After 70 years of research in metallocene chemistry, new innovative synthetic methods allowed now access to monofunctionalized rhodocenium salts containing useful functional groups which were characterized by spectroscopic methods (multinuclear NMR, IR, HR‐MS, XRD) and cyclic voltammetry.

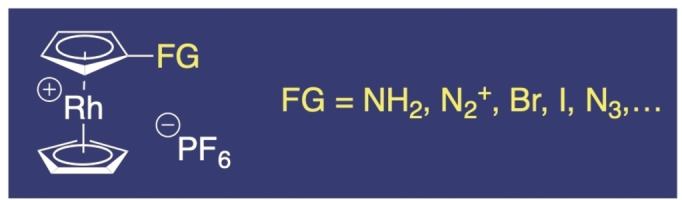

## Introduction

Metallocenes in general constitute a huge class of important organometallic compounds with examples throughout the periodic table of elements. By far, the most representatives are ferrocenes with their many useful applications in coordination chemistry and catalysis, materials science, redox chemistry, medicinal chemistry, etc. In contrast, the isoelectronic 18 valence electron cationic metallocenes of group 9‐cobaltocenium, rhodocenium and iridocenium‐are very much less studied, due to less convenient synthetic accessibility and functionalization caused by their deactivating positive charge preventing “standard” organic derivatization protocols. Chemically, cobaltocenium, rhodocenium and iridocenium salts are interesting for a number of reasons: (i) They are highly stable and non‐air‐sensitive, (ii) they have a rich electrochemistry displaying usually reversible (Co) or irreversible (Rh, Ir) redox events, (iii) they are highly polar with high solubility in polar solvents, including water, (iv) they are strongly electron‐withdrawing causing unusual reactivities with nucleophiles or of their appended functional groups, and‐last but not least‐(v) they represent a synthetic challenge even after 70 years of metallocene chemistry. Conceptually, we believe that functionalized Co/Rh/Ir metallocenium compounds will be valuable new building blocks in organometallic chemistry in general and in coordination chemistry in polar solvents, redox catalysis and redox sensing, or medicinal chemistry.

To this end, we have developed in the last couple of years new synthetic strategies in the area of cobaltocenium chemistry^[1]^ and now we put further efforts into rhodocenium functionalization. As a first contribution, we recently published our results on rhodocenium monocarboxylic acid hexafluoridophosphate and its derivatives^[2]^ and here we report on nitrogen and phosphorus derivatives, including the very important, synthetically very valuable amino and diazonio rhodocenium salts.

## Results and Discussion

### Synthesis

**Scheme 1:**
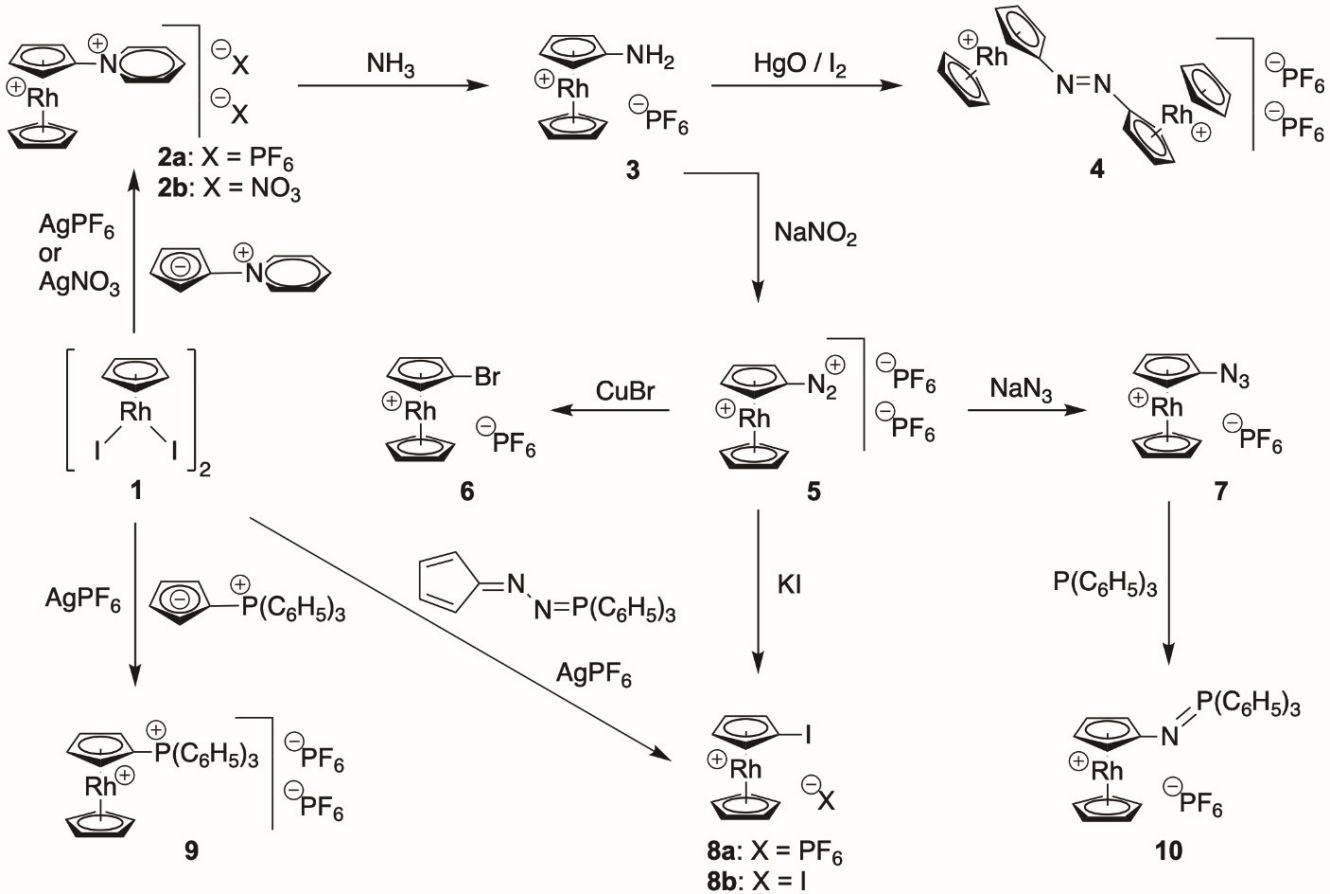
Synthesis of functionalized mono and dicationic rhodocenium salts **2**–**10**.

In comparison to cobalt organometallic chemistry, a distinctive feature of rhodium chemistry is the higher stability of half‐sandwich CpRhX_2_ compounds (X=halogen)^[3]^ that are obvious precursors for rhodocenium complexes by metathesis reactions with prefunctionalized Cp synthons (Scheme 1). To facilitate these transformations, the CpRhX_2_ synthons were first converted with AgPF_6_ to substitution‐labile [CpRh]^2+^‐solvato complexes, driven by the precipitation of insoluble AgX salts. Reaction of cyclopentadienyldiiodorhodium dimer (**1**)^[3]^ with two equivalents of silver hexafluoridophosphate followed by two equivalents of pyridinium cyclopentadienide^[4]^ afforded air‐stable pyridiniorhodocenium bis(hexafluoridophosphate (**2** **a**) in 82 % yield. In a similar manner, pyridiniorhodocenium dinitrate (**2** **b**) was generated using silver nitrate instead of silver hexafluoridophosphate. From this compound, aminorhodocenium hexafluoridophosphate (**3**) was prepared in 66 % yield by a Zincke‐type reaction with aqueous ammonia. This is an operationally simple but mechanistically remarkable synthetic transformation: In the classical Zincke reaction,^[5]^ N‐substituted pyridinium salts are generated from 1‐(2,4‐dinitrophenyl)pyridinium chloride with secondary amines (usually aniline derivatives) by a nucleophilic addition (A_N_), ring opening and ring closing (ANRORC) mechanism under formation of 2,4‐dinitroaniline as “waste” product. Essentially, an amino group is formally exchanged from the more electron‐rich aniline derivative to the electron‐poor dinitrophenyl group. Here we are not interested in the usual Zincke‐pyridinium product but in the amino side product. In an organometallic Zincke‐type reaction, we use the strongly electron‐withdrawing rhodocenium moiety as an electron‐acceptor of similar strength to the Zincke salt, thereby realizing an atom‐economic amino transfer from aqueous ammonia to rhodocenium with formation of unsubstituted pyridine and/or the Zincke aldehyde as the waste product(s). This synthetic protocol using pyridiniorhodocenium (**2** **b**) as an amino precursor was designed by us because unsubstituted CpNH_2_ as a potential reagent for half‐sandwich capping reactions does not exist, in contrast to disubstituted CpNR_2_ compounds.^[6]^ Potentially, this Zincke‐amination protocol might be also applicable in the future to other organometallic salts, likely candidates would be dicationic pyridinio‐substituted benzenetricarbonylmanganese or benzenecyclopentadienyliron complexes.

Aminorhodocenium hexafluoridophosphate (**3**) was selectively oxidized with the HgO/I_2_ reagent^[7]^ in 89 % yield to orange, water‐soluble, dicationic dirhodoceniumdiazene (**4**), an azorhodocenium compound analogous to azocobaltocenium that became recently available by our previous work.^[1g]^ Aminorhodocenium hexafluoridophosphate (**3**) is clearly a highly valuable compound due to the very rich chemistry of the amino functional group in organic chemistry. Diazoniation under standard conditions in water afforded beige‐colored diazoniorhodocenium bis(hexafluoridophosphate) (**5**) in 60 % yield, similar as the recently published diazoniation of aminocobaltocenium hexafluoridophosphate.^[1d]^ Diazonium salts in general are privileged synthons in organic chemistry allowing various nucleophilic and free‐radical substitution reactions,^[8]^ enabled by the superior leaving group N_2_. Dicationic **5** is a highly reactive, special diazonium salt with similar unique properties as its cobaltocenium congener:^[1d]^ Reactions in solution are more or less restricted to nitromethane as solvent, other more common solvents lead to decomposition. Therefore, small‐scale Sandmeyer reactions are preferably performed without solvent by ball‐milling, e. g. reaction of solid **5** with cuprous bromide afforded bromorhodocenium hexafluoridophosphate (**6**) in 86 % yield, or in nitromethane solution, e. g. reaction with sodium azide gave azidorhodocenium hexafluoridophosphate (**7**) in 62 % yield and with potassium iodide iodorhodocenium iodide (**8** **b**) in 84 % yield. Clearly many other synthetic applications of **5** may be envisaged, for example azo‐coupling with arenes would give access to redox‐responsive rhodocenium azo dyes. An alternative and shorter route to iodo‐substituted rhodocenium salts was developed by the reaction of starting material cyclopentadienyldiiodorhodium dimer (**1**) with one equivalent of silver hexafluoridophosphate per rhodium atom and with diazocyclopentadiene or its more stable and easier to handle triphenylphosphine‐adduct^[9]^ under insertion of the transient cyclopentadienylidene into the rhodium‐iodine bond, in analogy to such reactions with ruthenium^[10]^ or manganese and rhodium^[11]^ halide complexes, affording thereby iodorhodocenium hexafluoridophosphate (**8** **a**) in 55 % isolated yield. Furthermore and conceptionally similar to the preparation of pyridiniorhodocenium **2**, half‐sandwich capping reaction of **1** with triphenylphosphonium cyclopentadienide[^4a^, ^12^] gave slightly air‐sensitive, dicationic phosphonio‐substituted rhodocenium salt **9** in 90 % yield, potentially useful for Pd‐catalyzed cross‐coupling reactions.^[13]^ Finally, Staudinger reaction of azidorhodocenium hexafluoridophosphate (**7**) with triphenylphosphine gave access to monocationic iminophosphorane (**10**) in 91 % yield as an orange, air‐stable complex. In contrast to the classical Staudinger reaction, no hydrolysis to aminorhodocenium (**3**) was observed, and, synthetically much more important, no aza‐Wittig reaction^[14]^ with aldehydes proved possible. However, it is known that electron‐poor azides, e. g. perfluoroaryl azides, afford air and water‐stable iminophosphoranes that have current applications in bioorthogonal derivatization.^[15]^ Obviously, the cationic rhodocenium moiety is similarly electron‐withdrawing, as was also observed in the cobaltoceniumiminophosphorane congener.^[16]^

Overall, these synthetic methods allowed access to new valuable functionalized rhodocenium salts with prospects for further development of this class of compounds. However, a general problem with this chemistry is the difficulty to obtain pure compounds: chromatography is usually not possible and separation of various rhodocenium species, e. g. undesired unsubstituted rhodocenium from target substituted rhodocenium salts, by selective precipitation and/or crystallization is not easy to achieve and results in loss of material. Therefore, carefully optimized reaction conditions are mandatory. Moreover, rhodium chemicals are quite expensive, therefore mostly small‐scale reactions are feasible.

### Physical, Spectroscopic, and Structural Properties

Mono or dicationic rhodocenium derivatives **2**–**10** are highly polar, air‐stable salts (except slightly air‐sensitive **9**) of yellow to orange color, soluble in polar solvents like acetonitrile, nitromethane, methanol, acetone, dichloromethane and water.

Positive mode electrospray high‐resolution mass spectra gave signals of their most abundant monoisotopic peaks of the cationic moieties in excellent agreement with calculated values, except for azido derivative **7** where matrix‐induced degradation via the nitrene intermediate to the amino derivative **3** was evident.

ATR‐IR spectra are dominated by the very strong absorptions of the PF_6_ anions (≈820 and 550 cm^−1^) and show diagnostic bands for dipolar functional groups (compare Experimental Section), e. g. the diazonio substituent of **5** gave a weak absorption at 2289 cm^−1^ and the azido substituent of **7** showed a medium strong band at 2025 cm^−1^.

**Figure 1:**
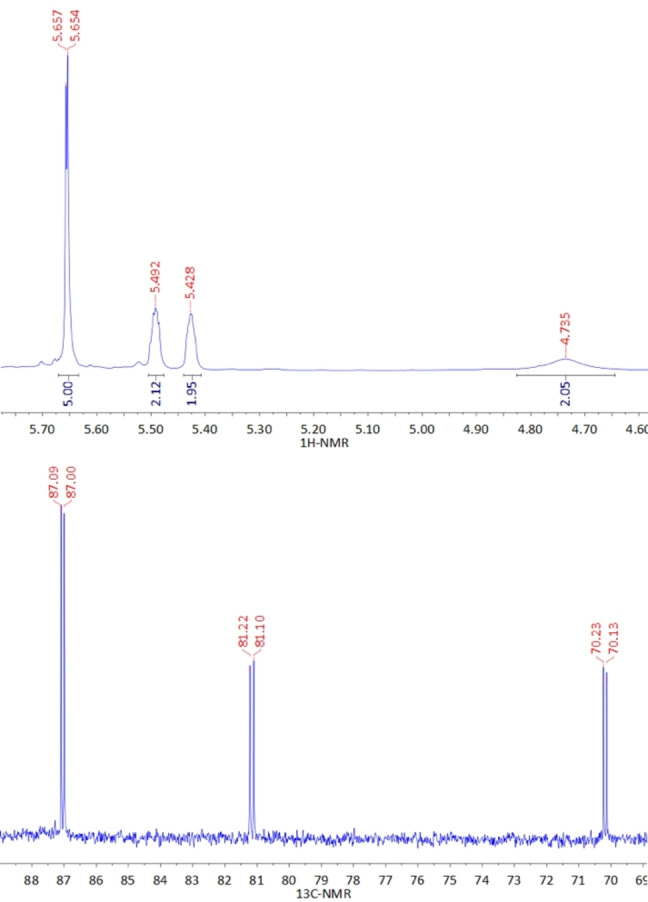
^1^H (top) and ^13^C (bottom) NMR spectra of aminorhodocenium hexafluoridophosphate (**3**) in CD_3_CN solution.

^1^H‐NMR spectra of these rhodocenium compounds display the common principal pattern of monosubstituted metallocenes (one singlet, 5 H, unsubstituted Cp and two pseudo‐triplets, 2×2H, substituted Cp) in the range of 5.4–7.4 ppm with additional ^2^ *J*(^1^H‐^103^Rh) couplings of approximately 1 Hz to the ^103^Rh nucleus (S=1/2 , 100 % natural abundancy), thereby each unsubstituted Cp is observed as a doublet and each hydrogen signal of the substituted Cp is observed as a poorly resolved multiplet (see Supporting Information). As a representative example, Figure 1 shows the ^1^H and ^13^C spectra of aminorhodocenium hexafluoridophosphate (**3**). In addition, hydrogen signals of the attached substituents are observed where applicable, e. g. amino resonances for **3** and pyridine or phenyl resonances for compounds **2**, **9** and **10**. As expected, these latter signals are shifted to lower field in comparison to those of “normal” (het)aryl substituents, due to the positive charge of these pyridinio or phosphonium substituents.

In the ^13^C‐NMR spectra of **2**–**10**, carbon resonances of the rhodocenium sandwich moieties are easily identified as doublets with ^1^ *J*(^13^C−^103^Rh) coupling constants of 6–9 Hz; see for example Figure 1 (bottom) for **3**. The chemical shift range of the rhodocenium signals is in the range from 70–100 ppm, a spectral area rarely covered by organic compounds. Furthermore, characteristic ^31^P‐NMR signals with corresponding couplings to ^13^C (see Supporting Information) are observed for compounds **9** and **10**.

**Figure 2:**
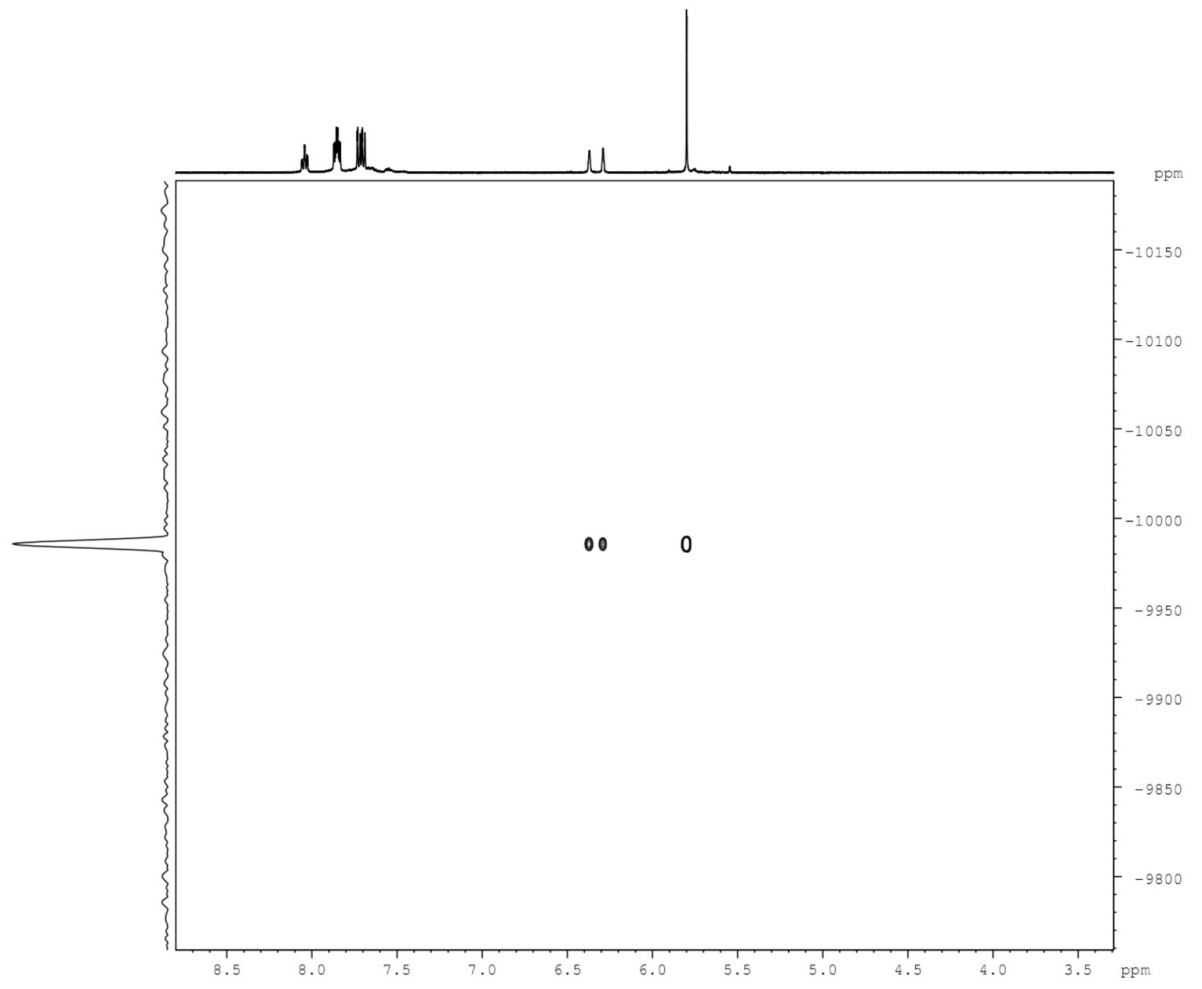
2D‐^1^H‐^103^Rh‐HMQC spectrum of triphenylphosphoniorhodocenium bis(hexafluoridophosphate) **9** in CD_3_CN solution.

^103^Rh‐NMR is a special case: On the one hand, ^103^Rh is a nucleus with “good” NMR properties (S=1/2 , 100 % natural abundance), but on the other hand its extremely low gyromagnetic ratio (λ=−0.8420×10^7^ radT^−1^s^−1^), poor receptivity (0.2 % of that of ^13^C) and huge chemical shift range (≈13000 ppm) makes it in practice very difficult to obtain NMR data. Moreover, most standard multinuclear NMR spectrometers are not equipped for detecting ^103^Rh signals. However, in diamagnetic Rh(I) or Rh(III) complexes with resolved scalar couplings to other nuclei of spin 1/2 , application of magnetization transfer pulse sequences allow considerable increase in sensitivity with concomitant shorter data acquisition periods.^[17]^ Therefore we set out to try this technique on our family of new monosubstituted rhodocenium compounds having ^2^ *J*(^1^H‐^103^Rh) couplings of approximately 1 Hz, aiming at a possible correlation of their ^103^Rh chemical shifts with inductive or resonance substituent parameters.^[18]^ In our first paper on rhodocenium chemistry,^[2]^ we experienced some difficulties obtaining reliable ^103^Rh‐NMR data, but here in a second attempt we succeeded using carefully optimized spectrometer settings on selected compounds (**3**, **4**, **7**, **8** **a**, **9**, see Experimental Section). For comparison, we include also data of some rhodocenium carboxylic acid derivatives obtained earlier^[2]^ (Table 1). Figure 2 shows the HMQC spectrum of **9** as a representative example with nicely resolved ^2^ *J*(^1^H‐^103^Rh) couplings to all hydrogens of both cyclopentadienyl ligands.

**Table 1:** ^103^Rh‐NMR data of monosubstituted rhodocenium salts.

Compound^[a]^	Formula^[b]^	[ppm] (^103^Rh)^[c]^
	[Rc−H]PF_6_	−10127
	[Rc−CH_3_]PF_6_	−10059
	[Rc−CO_2_H]PF_6_	−9997
	[Rc−CO_2_CH_3_]PF_6_	−9991
	[Rc−CO_2_NH_2_]PF_6_	−10000
**3**	[Rc−NH_2_]PF_6_	−9820
**4**	[Rc−N=N−Rc](PF_6_)_2_	−9921
**7**	[Rc−N_3_]PF_6_	−9949
**8** **a**	[Rc−I]PF_6_	−9976
**9**	[Rc−P(C_6_H_5)3_](PF_6_ )_2_	−9986

[a] Unnumbered rhodocenium carboxylic acid derivatives were synthesized according to reference 2. [b] Rc=rhodoceniumyl, [(C_5_H_5_)Rh(C_5_H_4_)]^+^. [c] Chemical shifts (ppm) are referenced against external Rh(acac)_3_.

Overall, the ^103^Rh chemical shifts of the parent unsubstituted rhodocenium and of these nine monosubstituted rhodocenium complexes span a range of approximately 300 ppm with aminorhodocenium hexafluoridophosphate (**3**) showing the most deshielded and unsubstituted rhodocenium hexafluoridophosphate the most shielded value, respectively. This is quite counterintuitive to simple donor/acceptor considerations, indicating that **3** is an unusual amine, as we will also see in its solid‐state structure discussed below. Neglecting the ^103^Rh‐NMR value of **3** and attempting to correlate the other data with Hammett substituent parameters^[18]^ gave no linear regression at all, reflecting that no simple resonance and inductive phenomena are operating in this family of compounds.

**Figure 3:**
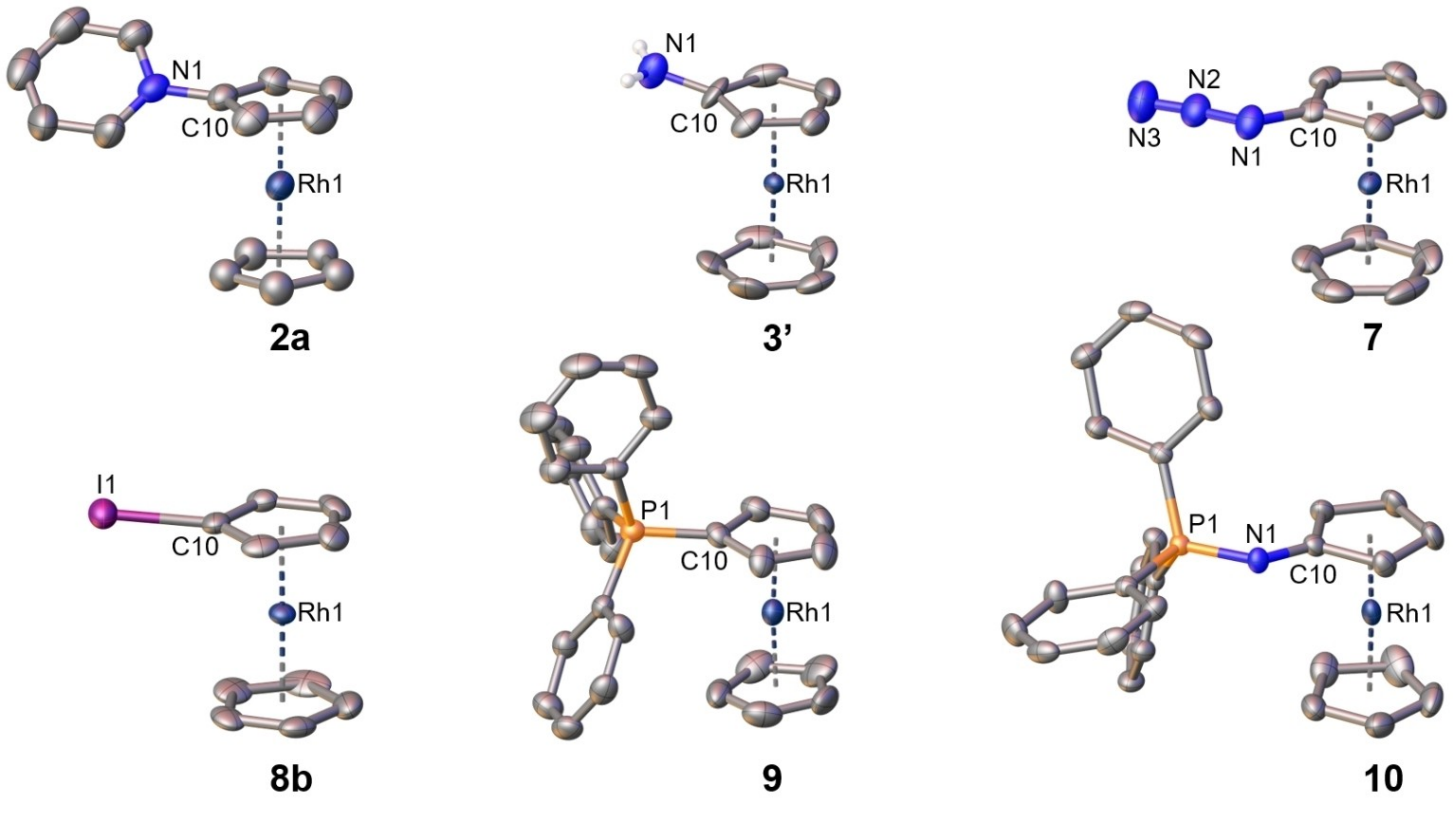
Molecular structures of rhodocenium salts with thermal ellipsoids at a 50 % probability, counterions PF_6_/Cl/I and solvent molecules omitted for clarity. Selected bond lengths [Å]: **2** **a**: C10‐N1=1.437(8); **3’**: C10‐N1=1.331(13); **7**: C10‐N1=1.398(3), N1‐N2=1.253(3), N2‐N3=1.124(3); **8** **b**: C10‐I1=2.095(7); **9**: C10‐P1=1.799(5); **10**: C10‐N1=1.351(3), N1‐P1=1.5983(18).

Single crystal X‐ray structure analyses are available for complexes **2**, **3’** (chloride instead of hexafluoridophosphate as counterion), **7**, **8** **b**, **9** and **10**. Molecular structures of the cations are depicted in Figure 3 and details of the refinement and other structural data are available in the Supporting Information. Overall, these simple rhodocenium salts show the expected regular structures with normal, undistorted sandwich moieties containing parallel cyclopentadienyl ligands. Rhodium‐carbon bond lengths are in the usual range of 2.15‐2.19 Å^[2]^ and the appended functional groups display common structural metrics. There are only two details that need further comment: First, the carbon‐nitrogen bond length in aminorhodocenium **3’** is shortened [C10‐N1=1.34 Å] in comparison to a standard C−N single bond, indicative of partial iminium/fulvene bonding, a structural motif generally observed in aminometallocenium salts.^[1d]^ This is in line with the unusual ^103^Rh‐NMR shift of **3** (vide supra) and with a reduced Brønsted‐basicity of the amino group of **3**. Second, whereas the cationic part of iodorhodocenium iodide **8** **b** is quite normal, the counterion iodide shows an interesting halogen‐bonding^[19]^ to a diiodine molecule with 50 % occupancy in the unit cell (see Supporting Information). All in all, these structures give further definite proof of the identity of these new rhodocenium compounds.

### Electrochemistry

**Figure 4:**
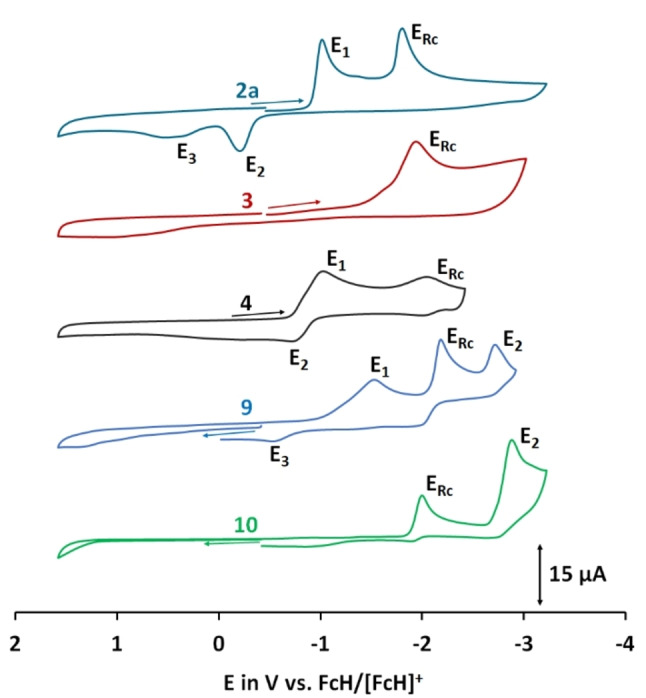
Cyclic voltammograms of complexes **2** **a**, **3**, **4**, **9** and **10** in acetonitrile. Measurements conditions: 0.2 M NBu_4_PF_6_ electrolyte and 0.001 M analyte; referenced vs. ferrocene/ferrocenium couple as an external standard; scan rate 100 mV s^−1^. E_Rh_ indicates rhodocenium centered redox processes, E_1_ to E_3_ indicate follow up redox events.

Due to the limited amount of material available for some compounds, only selected materials were studied by cyclic voltammetry. All rhodocenium salts investigated (**2** **a**, **3**, **4**, **9**,**10**) show irreversible rhodocenium centered redox‐processes E_Rc_ between −1.83 and −2.20 V vs. the ferrocene/ferrocenium couple (Figure 4, Table 2). Compared to our previous reported rhodocenium complexes,^[2]^ these potentials are slightly anodically shifted to lower potentials, which can be easily explained by the more electron donating character of the cyclopentadienyl substitution patterns. In addition to the expected rhodocene/rhodocenium redox event, the complexes **2** **a**, **4**, **9** and **10** show also further redox processes depending on their substitution pattern: Complex **2** **a** shows a reduction at −1.03 V (E1) which is coupled to two redox events E2 and E3 and is most likely centered on the pyridinium substituent. Complex **4** shows a broad redox feature at −1.04 V and −0.72 V due to the azo bridge between the two rhodocenium centers, less resolved in comparison to its cobaltocenium congener.^[1g]^ Complex **10** shows a second reduction at −2.89 V which is most likely a P^V^/P^IV^ redox couple. The irreversible redox processes E1 and E2 in the CV of **9** may be explained similarly, reducing the P(V) center to P(IV).^[20]^

**Table 2:** Overview of redox potential of selected rhodocenium salts.^[a]^

Complex	E_Rc_	E_1_	E_2_	E_3_
**2** **a**	−1.83 V	−1.03 V	−0.19 V	0.34 V
**3**	−1.96 V	–	–	–
**4**	−2.04 V	−1.04 V	−0.72 V	‐
**9**	−2.01 V	−2.89 V	–	–
**10**	−2.20 V	−1.54 V	−2.72 V	−0.54 V

[a] Potentials are referenced versus an external ferrocene/ferrocenium couple. E_Rc_ indicates rhodocenium centered redox processes, E_1_ to E_3_ indicate follow up redox events.

## Conclusion

The synthesis of a range of new functionalized rhodocenium salts was achieved by capping reactions of half‐sandwich CpRhX_2_ starting materials with zwitterionic pyridinium/phosphonium‐Cp synthons followed by an unusual application of the Zincke reaction, diazoniation, Sandmeyer reactions, and Staudinger reaction. The new mono‐functionalized rhodocenium complexes are air‐stable materials that were fully characterized by high‐resolution mass spectrometry, IR spectroscopy, ^1^H, ^13^C and‐most notably‐also by ^103^Rh NMR spectroscopy. Single crystal structure analyses of all target compounds show the expected regular sandwich structure of simple monofunctionalized metallocenes. Cyclic voltammetry of selected representatives show irreversible rhodocenium/rhodocene reductions and additional redox events dependent on the substitution pattern. Overall, with this research we give access to chemically useful new rhodocenium salts, thereby fostering the future development of this so far rarely studied class of compounds.

## Experimental Section

**General procedures**: Synthetic operations were performed by standard organometallic methods in Schlenk glassware under an inert atmosphere of Ar. Solvents were purified and dried in a solvent purification system with molecular sieves as water scavengers under an atmosphere of Ar. Chemicals were obtained commercially and used as received. Starting material cyclopentadienyldiiodorhodium dimer (**1**) was synthesized according to a published procedure.^[3]^^1^H, ^13^C, ^31^P NMR spectra were recorded on a Bruker DPX 300 NMR spectrometer and signals were referenced internally against ^1^H/^13^C residual solvents peaks or externally (^31^P) against 85 % aqueous phosphoric acid. ^1^H−^103^Rh‐HMQC NMR experiments were performed on a Bruker AVII+500 NMR spectrometer using 5 mm tubes with a 10 mm low‐gamma probe head operating at 500.13 (^1^H) and 15.80 MHz (^103^Rh) and following a two‐step strategy. First, the ^103^Rh resonance was located by monitoring the ^1^H NMR doublet of the unsubstituted Cp ligand and stepping the ^103^Rh decoupler through the anticipated ^103^Rh chemical shift range. Secondly, the exact ^103^Rh chemical shift was established from a ^1^H‐^103^Rh‐HMQC NMR experiment (Bruker pulse program hmqcph). For ^103^Rh NMR referencing the IUPAC reference standard with Ξ=3.186447 %^[21]^ in combination with the ^2^H resonance of the solvent has been used, corresponding to external Rh(acac)_3_. Samples for comparison for ^103^Rh‐NMR data, rhodocenium hexafluoridophosphate [(RcH)PF_6_], methylrhodocenium hexafluoridophosphate [(RcCH_3_)PF_6_], rhodoceniumcarbocylic acid hexafluoridophosphate [(RcCO_2_H)PF_6_], methoxycarbonylrhodocenium hexafluoridophosphate [(RcCO_2_CH_3_)PF_6_] and rhodoceniumcarboxylic acid amide hexafluoridophosphate [(RcCONH_2_)PF_6_] were obtained as recently published.^[2]^ Mass spectrometric data were measured on a Thermo Finnigan Q Exactive Orbitrap spectrometer, IR spectra were performed on a Bruker ALPHA IR spectrometer, single crystal X‐ray diffraction data were collected on a Bruker D8 Quest diffractometer with graphite‐monochromated Mo−Kα radiation (λ=0.71073 Å) and structures were solved by direct methods. Cyclovoltammetry measurements were performed inside an Ar‐filled glove‐box using a BioLogic SP 150 potentiostat with a three‐electrode setup: glassy carbon (working electrode), platinum (counter electrode), silver (pseudo reference).

**Pyridiniorhodocenium bis(hexafluoridophosphate) (2** **a)**: A Schlenk tube was charged under an atmosphere of Ar with 234.8 mg (0.5566 mmol, 1 equiv.) [CpRhI_2_]_2_ (**1**), 281.4 mg (1.113 mmol, 2 equiv.) AgPF_6_ and 25 mL of dry acetone. Under exclusion of light the mixture was stirred for half an hour, resulting in an orange solution and a slightly yellow precipitate of AgI. To this mixture, 79.7 mg (0.5566 mmol, 1 equiv.) pyridiniumcyclopentadienide^[4]^ was added under protection from air and stirring was continued at room temperature overnight. Workup under ambient conditions without protection from air: The solution was concentrated to half of its volume on a rotary evaporator, diethyl ether was added to precipitate the crude product, the mixture was filtered and the residue was dissolved in acetone, filtered again, the solution was evaporated on a rotary evaporator, and the oily residue was dried in vacuo, resulting in 275.7 mg (0.4586 mmol, 82.4 % yield) of yellow air‐stable **2** **a**. Compound **2** **a** is soluble in acetone, acetonitrile, nitromethane and nitroethane. ^1^H‐NMR (300 MHz, CD_3_CN, 25 °C): δ=6.06 (d, ^2^ *J*(^1^H‐^103^Rh)=0.95 Hz, 5 H, Cp), 6.08 (m, 2 H, Cp_subst._), 6.63 (m, 2 H, Cp_subst._), 8.17 (m, 2 H, py), 8.73 (m, 1 H, py), 8.96 (m, 2 H, py) ppm. ^13^C‐NMR (75 MHz, CD_3_CN, 25 °C): δ=85.05 (d, ^1^ *J*(^13^C‐^103^Rh)=5.88 Hz, 3,4‐Cp_subst._), 88.29 (d, ^1^ *J*(^13^C‐^103^Rh)=7.42 Hz, 2,5‐Cp_subst._), 91.02 (d, ^1^ *J*(^13^C‐^103^Rh)=7.26 Hz, Cp), 129.85, 146.44, 150.23 (py) ppm; not observed: C‐1 signal of Cp_subst._). IR (ATR): ν=3126 (ν_C‐H_), 2927, 1727, 1630, 1486, 1460, 1418, 1298, 1136, 1036, 817 (ν_P‐F_), 740, 673, 634, 555 (ν_P‐F_), 493 cm^−1^. HRMS (ESI pos): C_15_H_15_NRh, calcd. most abundant isotope peak 155.5085 ([M ‐ 2 PF_6_]^2+^), found m/z=155.5083. Single crystal structure XRD analysis: Suitable crystals were obtained by diffusion of diethyl ether into a nitroethane solution of **2** **a** at 8 °C (Figure 3, Supporting Information).

**Aminorhodocenium hexafluoridophosphate (3)**: Pyridiniorhodo‐cenium di(nitrate) **2** **b** was prepared in an analogous manner as pyridiniumrhodocenium bis(hexafluoridophosphate) **2** **a** from a methanol solution of [CpRhI_2_]_2_ (**1**), two equivalents of AgNO_3_, and two equivalents of pyridiniumcyclopentadienide.^[4]^ A round bottom flask was charged under ambient conditions without protection from air with 185.9 mg (0.4271 mmol) of pyridiniorhodocenium di(nitrate) and 10 mL of 10 % aqueous ammonia was added, resulting in a magenta solution which was stirred overnight. Workup: Solvents and volatile materials were removed on a rotary evaporator, the residue was washed with a 1 : 1‐mixture of acetone/dichloromethane followed by isopropanol, the remaining crude product was dissolved in ethanol, solid materials were filtered off through a paper filter, and solvent was removed on a rotary evaporator, resulting in a light orange oil which was dried in vacuo. The yield of this material is >100 % because of admixture with some NH_4_NO_3_. To obtain pure **3**, an aqueous solution of NaPF_6_ (71.1 mg, 0.4271 mmol) was added to a concentrated aqueous solution of aminorhodocenium nitrate cooled to 4 °C. The resulting precipitate was filtered off, washed with isopropanol and diethyl ether, and dried in vacuo, affording 110.6 mg (0.2815 mmol, 65.9 % yield) pure **3** as a yellow powder. ^1^H‐NMR (300 MHz, CD_3_CN, 25 °C): δ=4.74 (br s, 2 H, NH_2_), 5.43 (unresolved m, 2 H, Cp_subst._), 5.49 (unresolved m, 2 H, Cp_subst._), 5.65 (d, ^2^ *J*(^1^H‐^103^Rh)=1.0 Hz, 5 H, Cp) ppm. ^13^C‐NMR (75 MHz, CD_3_CN, 25 °C): δ=70.45 (d, ^1^ *J*(^13^C‐^103^Rh)=7.7 Hz, Cp_subst._), 81.52 (d, ^1^ *J*(^13^C‐^103^Rh)=9.2 Hz, Cp_subst._), 87.40 (d, ^1^ *J*(^13^C‐^103^Rh)=6.9 Hz, Cp) ppm; not observed: C‐1 signal of Cp_subst._). ^103^Rh‐NMR (15.8 MHz, CD_3_CN, 25 °C): δ=‐9820 ppm. IR (ATR): ν=3399 (ν_N‐H_), 3124 (ν_C‐H_), 1632, 1513, 1416, 1218, 1049, 819 (ν_P‐F_), 739, 630, 555 (ν_P‐F_) cm^−1^. HRMS (ESI pos): C_10_H_11_NRh, calcd. most abundant isotope peak 247.9941 ([M ‐ PF_6_]^+^), found m/z=247.9936. Single crystal structure XRD analysis: Single crystals of **3’** (chloride instead of hexafluoridophosphate) were obtained from an aqueous solution in diluted hydrochloric acid at 4 °C. (Figure 3, Supporting Information).

**Dirhodoceniumyldiazene (“Azorhodocenium”) bis(hexafluorido‐phosphate) (4)**: A round bottom flask was charged with 170.6 mg (0.4340 mmol, 1 equiv) aminorhodocenium hexafluoridophosphate (**3**), 235.0 mg (1.085 mmol, 2.5 equiv) HgO, 275.4 mg (1.085 mmol, 2.5 equiv) I_2_ and 50 mL of reagent grade dichloromethane. After stirring at ambient conditions for 24 hours, 60 mL of diethyl ether was added and the resulting dispersion was filtered through a paper filter. The solid material in the paper filter was eluted with acetonitrile, then the solvent was evaporated on a rotary evaporator and the resulting orange product is dried in vacuo, affording 150.5 mg (0.1925 mmol, 88.7 % yield) **4**. ^1^H‐NMR (300 MHz, CD_3_CN, 25 °C): δ=5.93 (d, ^2^ *J*(^1^H‐^103^Rh)=0.9 Hz, 5 H, Cp), 6.04 (unresolved m, 2 H, Cp_subst._), 6.44 (unresolved m, 2 H, Cp_subst._) ppm. ^13^C‐NMR (75 MHz, CD_3_CN, 25 °C): δ=83.51 (d, ^1^ *J*(^13^C‐^103^Rh)=6.7 Hz, Cp_subst._), 89.09 (d, ^1^ *J*(^13^C‐^103^Rh)=7.3 Hz, Cp_subst._), 89.82 (d, ^1^ *J*(^13^C‐^103^Rh)=7.2 Hz, Cp) ppm; not observed: C‐1 signal of Cp_subst._). ^103^Rh‐NMR (15.8 MHz, CD_3_CN, 25 °C): δ=‐9921 ppm. IR (ATR): ν=3127 (ν_C‐H_), 2928, 1723, 1460, 1417, 1269, 1120, 1072, 817 (ν_P‐F_), 740, 554 (ν_P‐F_), 540, 469 cm^−1^. HRMS (ESI pos): C_20_H_18_N_2_Rh_2_, calcd. most abundant isotope peak 245.9785 (M^2+^ ‐ 2 PF_6_), found m/z=245.9785. Note: Since laboratory work had to be ceased because of the Covid‐19 pandemic, workup could not be optimized and some small impurities still remain seen in the NMR spectra (compare Supporting Information).

**Diazoniorhodocenium bis(hexafluoridophosphate) (5)**: In a small round bottom flask, 112.9 mg (0.2872 mmol, 1 equiv) aminorhodocenium hexafluoridophosphate (**3**) was dissolved in 3 mL of 60 % aqueous HPF_6_. After cooling to 4 °C, 29.7 mg (0.4308 mmol, 1.5 equiv) NaNO_2_ was added in form of a saturated aqueous solution and the solution was stirred for 10 minutes before letting it warm up to room temperature. The cream‐colored precipitate was filtered off on a porcelain frit, washed with ice‐cold water, a small amount of ice‐cold ethanol and diethyl ether, transferred to a round bottom flask and dried in vacuo, resulting in 84.7 mg (0.1539 mmol, 53.6 % yield) **5**. ^1^H‐NMR (300 MHz, CD_3_CN, 25 °C): δ=6.545 (broad s, 5 H, Cp), 6.633 (unresolved m, 2 H, Cp_subst._), 7.362 (unresolved m, 2 H, Cp_subst._) ppm. IR (ATR): ν=3134 (ν_C‐H_), 2289 (ν_N=N_), 1525, 1416 (ν_C=C_), 1344, 1207, 825 (ν_P‐F_), 739, 556 (ν_P‐F_), 416 cm^−1^. Note: Due to the high reactivity and limited stability of **5**, samples were freshly prepared and used subsequently in the following reactions.

**Bromorhodocenium hexafluoridophosphate (6)**: 10 mg (18.2 μmol, 1 equiv) diazoniorhodocenium hexafluoridophosphate) (**5**), 5.2 mg (36.4 μmol, 2 equiv), 4.4 mg (36.4 μmol, 2 equiv) KBr and 6 zirconia balls were combined in a ball mill container and milled twice for 10 minutes at 200 rpm. Workup: The solids were extracted with acetone, the solution was filtered, solvent was removed on a rotary evaporator and the residue was dried in vacuo, affording 7.1 mg (15.7 μmol, 86 % yield) of beige‐colored **6**. Note: This product contains appr. 14 % unsubstituted rhodocenium salts (hexafluoridophosphate and/or bromide), a common impurity in ball mill reactions of metallocenium diazonium salts.^[1d]^ ^1^H‐NMR (300 MHz, CD_3_CN, 25 °C): δ=5.82 (unresolved m, 2 H, Cp_subst._), 5.91 (d, ^2^ *J*(^1^H‐^103^Rh)=1.0 Hz, Cp), 6.22 (unresolved m, 2 H, Cp_subst._) ppm. Note: A ^13^C‐NMR spectrum could not be obtained due to the very limited amount of material available. HRMS (ESI pos): C_10_H_9_BrRh, calcd. most abundant isotope peak 310.8937 ([M‐PF_6_]^+^), found m/z=310.8937.

**Azidorhodocenium hexafluoridophosphate (7)**: A small Schlenk tube was charged with 17.3 (0.0315 mmol, 1 equiv) diazonio‐rhodocenium hexafluoridophosphate) (**5**) and 3 mL of dry nitromethane. The resulting solution was cooled to 4 °C and 5.7 mg (0.0944 mmol, 3 equiv) NaN_3_ was added. After stirring at 4 °C for 20 minutes, the mixture was allowed to warm to room temperature. Workup under ambient conditions: Solvent was removed on a rotary evaporator, the residue was dissolved in dichloromethane, the solution was filtered through a paper filter, solvent was removed on a rotary evaporator and the resulting product was dried in vacuo, affording 8.2 mg (0.0194 mmol, 61.6 % yield of dark yellow **7**. ^1^H‐NMR (300 MHz, acetone‐d_6_, 25 °C): δ=5.98 (unresolved m, 2 H, Cp_subst._), 6.17 (d, ^2^ *J*(^1^H‐^103^Rh)=1.0 Hz, Cp), 6.28 (unresolved m, 2 H, Cp_subst._) ppm. Note: A ^13^C‐NMR spectrum could not be obtained due to the very limited amount of material available. ^103^Rh‐NMR (15.8 MHz, CD_3_CN, 25 °C): δ=−9949 ppm. IR (ATR): ν=3116 (ν_C‐H_), 2956, 2025 (ν_N=N_), 1730, 1674, 1636, 1510, 1429, 1417, 1383, 1309, 1271, 1206, 1146, 1121, 1051, 1002, 822 (ν_P‐F_), 639, 557 (ν_P‐F_), 495 cm^−1^. HRMS (ESI pos): C_10_H_9_BrRh, calcd. most abundant isotope peak 245.9785 ([M ‐ N_2_ ‐ PF_6_]^+^), found m/z=247.9936 ([M‐N_2_‐PF_6_+2 H]^+^). Single crystal structure XRD analysis: Suitable crystals were obtained by slow evaporation of a methanol solution of **7** at room temperature. (Figure 3, Supporting Information).

**Iodorhodocenium hexafluoridophosphate (8** **a)**: A Schlenk vessel was charged under an atmosphere of Ar with 55.0 mg (0.130 mmol, 1 equiv) [CpRhI_2_]_2_ (**1**), 33 mg (0.130 mmol, 1 equiv) AgPF_6_ and 20 mL of dry acetonitrile. On stirring at room temperature, a dark orange suspension was obtained and 116 mg (0.326 mmol, 2.5 equiv) diazocyclopentadiene‐triphenylphosphane‐adduct^[9]^ was added. The mixture was heated to reflux overnight. Workup under ambient conditions: After cooling to room temperature, 20 mL of diethylether was added, the precipitate was filtered off on a paper filter, and the product was extracted with acetonitrile from the solids in the paper filter. Acetonitrile was removed on a rotary evaporator and after drying in vacuo 36 mg (0.0715 mmol, 55 % yield) **8** **a** was obtained. ^1^H‐NMR (300 MHz, CD_3_CN, 25 °C): δ=5.79 (unresolved m, 2 H, Cp_subst._), 5.86 (d, ^2^ *J*(^1^H‐^103^Rh)=1.0 Hz, Cp), 6.20 (unresolved m, 2 H, Cp_subst._) ppm. Note: A ^13^C‐NMR spectrum could not be obtained due to the limited amount of material available. ^103^Rh‐NMR (15.8 MHz, CD_3_CN, 25 °C): δ=‐9949 ppm. HRMS (ESI pos): C_10_H_9_IRh, calcd. most abundant isotope peak 358.8798 ([M ‐ PF_6_]^+^), found m/z=358.878.

**Iodorhodocenium iodide (8** **b)**: In a small Schlenk tube, 6.5 mg (11.8 μmol, 1 eq) diazoniorhodocenium bis(hexafluoridophosphate) (**5**) was dissolved in 3 mL of absolute nitromethane at 4 °C, then 7.9 mg (47.3 μmol, 4 eq) KI was added and the solution was stirred for 10 min. After letting it warm to room temperature, the mixture was stirred further overnight. Workup under ambient conditions: Solvent was evaporated nearly to dryness, then the same volume of diethyl ether was added and the precipitate was filtered off. The product was eluted from the filter with dichloromethane, the solvent was evaporated on a rotary evaporator and the residue was dried in vacuo. Yield: 5 mg (9.9 μmol, 84 %) **8** **b**. ^1^H‐NMR (300 MHz, acetone‐d_6_, 25 °C): δ=6.07 (unresolved m, 2 H, Cp_subst._), 6.13 (d, ^2^ *J*(^1^H‐^103^Rh)=1.0 Hz, Cp), 6.42 (unresolved m, 2 H, Cp_subst._) ppm. IR (ATR): ν=3084, 2957, 2926, 2857, 1724, 1601, 1460, 1405, 1377, 1268, 1120, 1072, 1040, 1014, 998, 839, 741, 704, 570, 412 cm^−1^. Single crystal structure XRD analysis: Suitable crystals were obtained by slow evaporation of a dichloromethane solution of **8** **b** at room temperature. (Figure 3, Supporting Information).

**Triphenylphosphoniorhodocenium bis(hexafluoridophosphate) (9)**: A Schlenk tube was charged under an atmosphere of Ar with 51 mg (0.1209 mmol, 1 equiv) [CpRhI_2_]_2_ (**1**), 61.1 mg (0.2418 mmol, 2 equiv) AgPF_6_ and 20 mL of dry acetonitrile. After stirring at room temperature for 30 minutes, 39.5 mg (0.1209 mmol, 1 equiv) of triphenylphosphoniocyclopentadienide^[12]^ was added and stirring was continued overnight. Workup under ambient conditions: The mixture was filtered, the solution was evaporated to dryness and the beige crystalline solid was dried in vacuo, affording 85.6 mg (0.1092 mmol, 90.3 % yield) **9**. The product is slightly hygroscopic and reacts with air over time to yield unsubstituted rhodocenium and other decomposition products and should be stored under inert atmosphere. ^1^H‐NMR (300 MHz, CD_3_CN, 25 °C): δ=5.77 (d, ^2^ *J*(^1^H‐^103^Rh)=0.99 Hz, 5H, Cp), 6.28 (unresolved m, 2 H, Cp_subst._), 6.35 (unresolved m, 2 H, Cp_subst._), 7.66 (m, 6 H, Ph), 7.81 (m, 6 H, Ph), 8.00 (m, 3 H, Ph) ppm. ^13^C‐NMR (75 MHz, CD_3_CN, 25 °C): δ=91.03 (d, ^1^ *J*(^13^C‐^103^Rh)=7.3 Hz, Cp), 93.30 (d×d, ^2^ *J*(^13^C‐^31^P)=8.29 Hz, ^1^ *J*(^13^C‐^103^Rh)=6.5 Hz, Cp_subst._), 94.57 (d × d, ^2^ *J*(^13^C‐^31^P)=11.4 Hz, ^1^ *J*(^13^C‐^103^Rh)=6.6 Hz, Cp_subst._), 131.86 (d, ^2^ *J*(^13^C‐^31^P)=13.6 Hz, o‐Ph), 135.31 (d, ^3^ *J*(^13^C‐^31^P)=11.3 Hz, m‐Ph), 137.57 (d, ^4^ *J*(^13^C‐^31^P)=3.1 Hz, p‐Ph) ppm. ^31^P‐NMR (40.5 MHz, CD_3_CN, 25 °C): δ=19.90 (d, ^1^ *J*(^31^P‐^103^Rh)=1.85 Hz, PPh_3_) ppm; (signal of PF_6_ not observed because out of spectral window). ^103^Rh‐NMR (15.8 MHz, CD_3_CN, 25 °C): δ=−9986 ppm. IR (ATR): ν=3119 (ν_C‐H_), 2924, 1710, 1587, 1485, 1439, 1416, 1396, 1300, 1178, 1136, 1111, 1073, 1040, 998, 902, 828 (ν_P‐F_), 751, 728, 692, 630, 616, 554 (ν_P‐F_), 528, 510, 453, 421, 410 cm^−1^. HRMS (ESI pos): C_28_H_24_PRh, calcd. most abundant isotope peak 247.0330 ([M ‐ 2 PF_6_]^2+^), found m/z=247.0324. Single crystal structure XRD analysis: Suitable crystals were obtained by slow evaporation of a dichloromethane solution of **9** at room temperature. (Figure 3, Supporting Information).

**Triphenylphosphazenerhodocenium hexafluoridophosphate (10)**: A small round bottom flask was charged with 13.4 mg (32.0 μmol, 1 equiv) azidorhodocenium hexafluoridophosphate (7), 8.4 mg (32.0 μmol, 1 equiv) triphenylphosphine and 3 mL of dry acetonitrile. The mixture was stirred a t room temperature for 30 minutes. Workup: Solvent was removed on a rotary evaporator, then the residue was dissolved in dichloromethane and the product was precipitated with diethyl ether. After filtration through a paper filter and washing with diethyl ether, the product was eluted from the filter with dichloromethane. Solvent was removed on a rotary evaporator and the remaining solid was dried in vacuo, affording 19.0 mg (29.1μmol, 90.9 % yield) of **10** as an orange, crystalline solid. ^1^H‐NMR (300 MHz, CD_3_CN, 25 °C): δ=5.10 (unresolved m, 2 H, Cp_subst._), 5.20 (d, 5 H, ^2^ *J*(^1^H‐^103^Rh)=1.0 Hz, Cp), 5.35 (unresolved m, 2 H, Cp_subst._), 7.63 (m, 6 H, o‐Ph), 7.76 (m, 9 H, m‐Ph+p‐Ph) ppm. ^13^C‐NMR (75 MHz, CD_3_CN, 25 °C): δ=75.90 (d × d, ^3^ *J*(^13^C‐^31^P)=18.0 Hz, ^1^ *J*(^13^C‐^103^Rh)=7.78 Hz, 2,5‐Cp_subst._), 81.37 (d, ^1^ *J*(^13^C‐^103^Rh)=9.05 Hz, 3,4‐Cp_subst._), 86.60 (d, ^1^ *J*(^13^C‐^103^Rh)=6.84 Hz, Cp), 127.59 (d, ^1^ *J*(^13^C‐^31^P)=101 Hz, ipso‐Ph), 130.83 (d, ^2^ *J*(^13^C‐^31^P)=12.4 Hz, o‐Ph), 133.97 (d, ^3^ *J*(^13^C‐^31^P)=10.2 Hz, m‐Ph), 134.70 (d, ^4^ *J*(^13^C‐^31^P)=2.79 Hz, p‐Ph) ppm. ^31^P‐NMR (40.5 MHz, CD_3_CN, 25 °C): δ=14.86 (s, NPPh_3_) ppm; (signal of PF_6_ not observed because out of spectral window). IR (ATR): ν=2959, 2927, 1721, 1483, 1437, 1410, 1384, 1268, 1107, 1067, 1019, 990, 857, 825, 754, 727, 693, 651, 631, 555, 542, 525, 507, 460, 441, 414 cm^−1^. HRMS (ESI pos): C_28_H_24_NPRh, calcd. most abundant isotope peak 508.0696 ([M ‐ PF_6_]^+^), found m/z=508.0688. Single crystal structure XRD analysis: Suitable crystals were obtained by slow evaporation of a dichloromethane solution of **10** at room temperature. (Figure 3, Supporting Information).

Supporting Information contains ^1^H/^13^C/^31^P/^103^Rh‐NMR, HR‐MS, IR spectra and cyclovoltammetry data for compounds **2** **a**–**10**.

Deposition Numbers 2087342 (for **2** **a**), 2087343 (for **3’**), 2087344 (for **7**), 2087345 (for **8** **b**), 2087346 (for **9**), and 2087347 (for **10**) contain the supplementary crystallographic data for this paper. These data are provided free of charge by the joint Cambridge Crystallographic Data Centre and Fachinformationszentrum Karlsruhe Access Structures service www.ccdc.cam.ac.uk/structures.

## Conflict of interest

The authors declare no conflict of interest.

## Supporting information

As a service to our authors and readers, this journal provides supporting information supplied by the authors. Such materials are peer reviewed and may be re‐organized for online delivery, but are not copy‐edited or typeset. Technical support issues arising from supporting information (other than missing files) should be addressed to the authors.

Supporting Information
