# Supplementary material for: Rhodocenium Functionalization Enabled by Half‐Sandwich Capping, Zincke Reaction, Diazoniation and Sandmeyer Chemistry
Source: Eur J Inorg Chem. 2021 Jul 16;2021(32):3305–13. doi: 10.1002/ejic.202100525 (PMC8456843; doi:10.1002/ejic.202100525)
Supplement: Supplementary file 1 — Supporting Information [file EJIC-2021-3305-s001.pdf]

European Journal of Inorganic Chemistry

Supporting Information

Rhodocenium Functionalization Enabled by Half-Sandwich Capping, Zincke Reaction, Diazoniation and Sandmeyer Chemistry

Markus Wiedemair, Holger Kopacka, Klaus Wurst, Thomas Müller, Klaus Eichele, Stefan Vanicek, Stephan Hohloch, and Benno Bildstein*

Table of Contents

1. NMR spectra, mass spectra and IR spectra 2

1.1 Pyridiniorhodocenium bis(hexafluoridophosphate) (2) 2

1.2 Aminorhodocenium hexafluoridophosphate (3) 4

1.3 Dirhodoceniumdiazene bis(hexafluoridophosphate) (4) 7

1.4 Diazoniorhodocenium bis(hexafluoridophosphate) (5) 10

1.5 Bromorhodocenium hexafluoridophosphate (6) 11

1.6 Azidorhodocenium hexafluoridophosphate (7) 12

1.6 Iodorhodocenium hexafluoridophosphate (8a) 15

1.6 Iodorhodocenium iodide (8b) 17

1.7 Triphenylphosphoniorhodocenium bis(hexafluoridophosphate) (9) 18

1.8 (Triphenyl)phosphazenerhodocenium hexafluoridophosphate (10) 22

2. Additional ^{103}Rh-NMR spectra related to previous compounds 25

2.1 Rhodocenium hexafluoridophosphate (11) 25

2.2 Methylrhodocenium hexafluoridophosphate (12) 26

2.3 Rhodococenium carboxylic acid hexafluoridophosphate (13) 27

2.4 Methyl rhodocenium carboxylate hexafluoridophosphate (14) 28

2.5 Rhodocenium carboxylic acid amide hexafluoridophosphate (15) 29

4. Literature 30

1. NMR spectra, mass spectra and IR spectra

1.1 Pyridiniorhodocenium bis(hexafluoridophosphate) (2a)

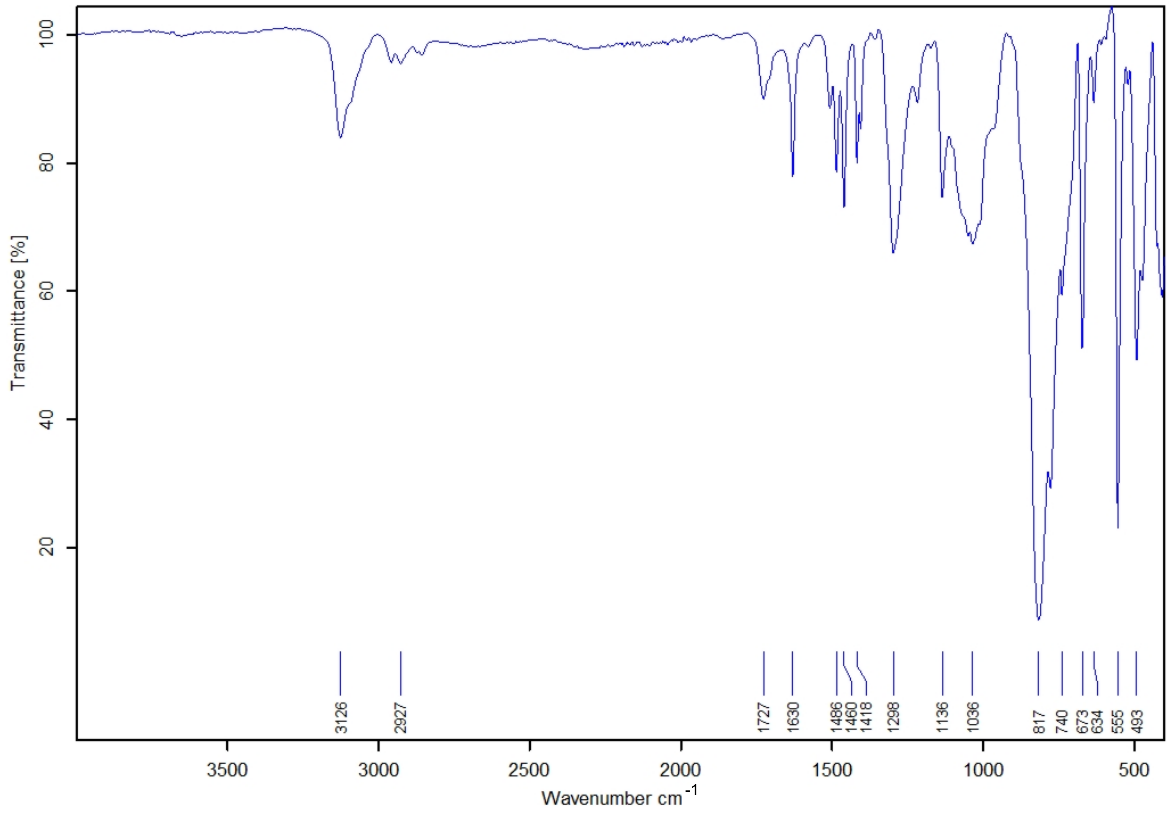

*Figure S1: ATR-IR spectrum of **2a**.*

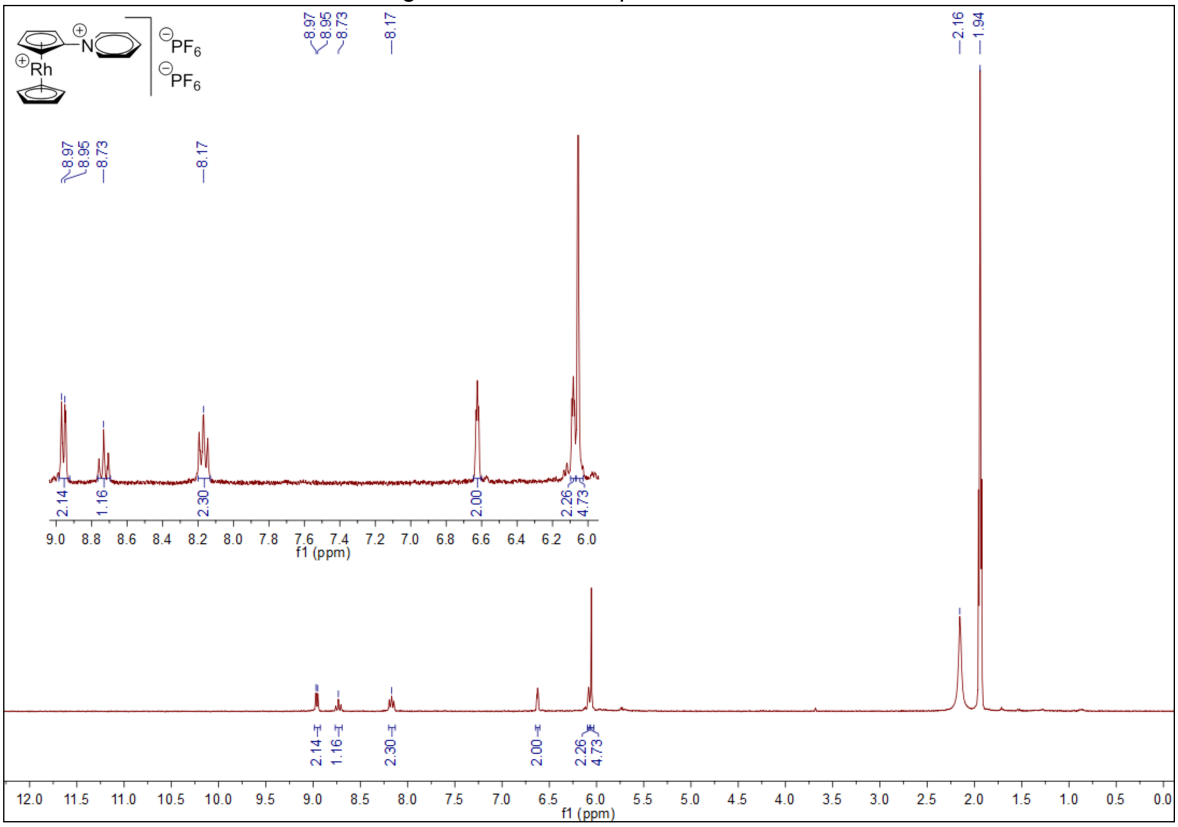

*Figure S2: ^{1}H-NMR of **2a** in CD_3CN.*

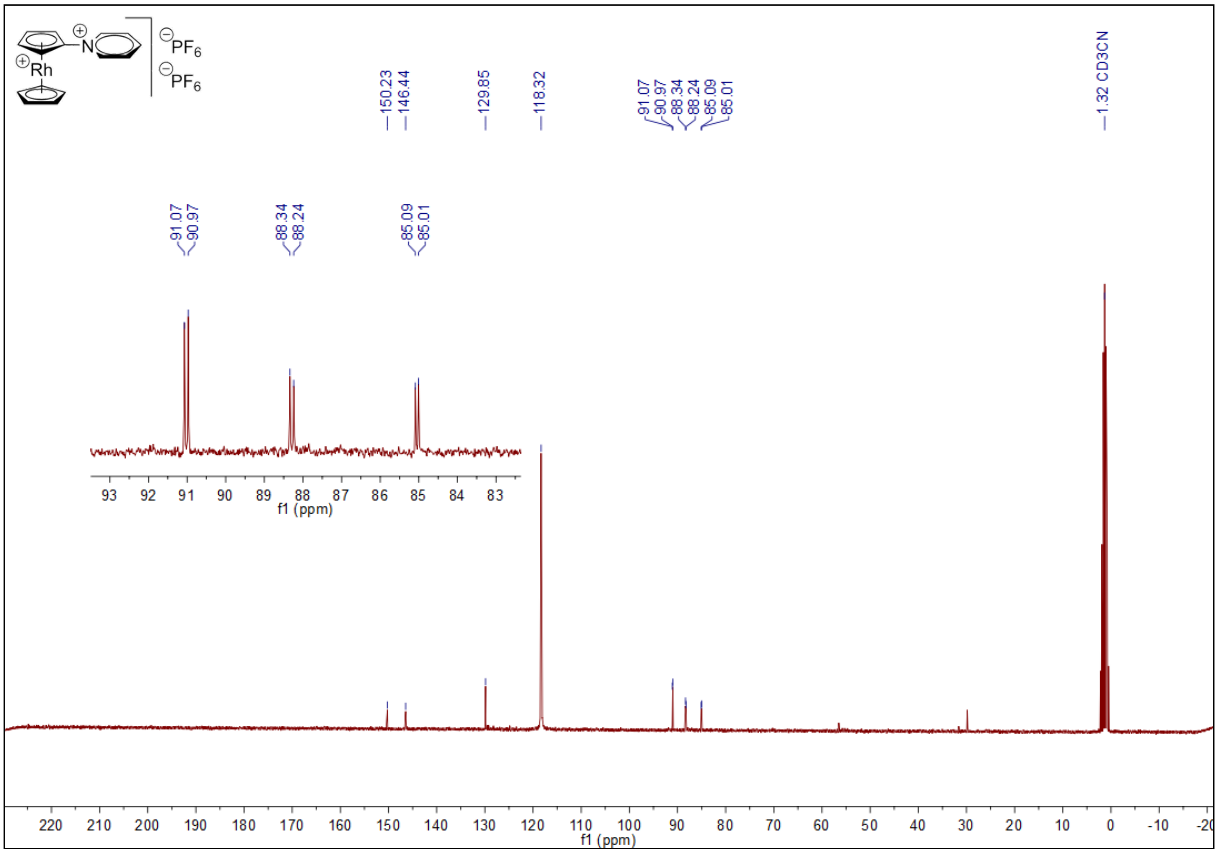

*Figure S3: ^{13}C-NMR of **2a** in CD_3CN.*

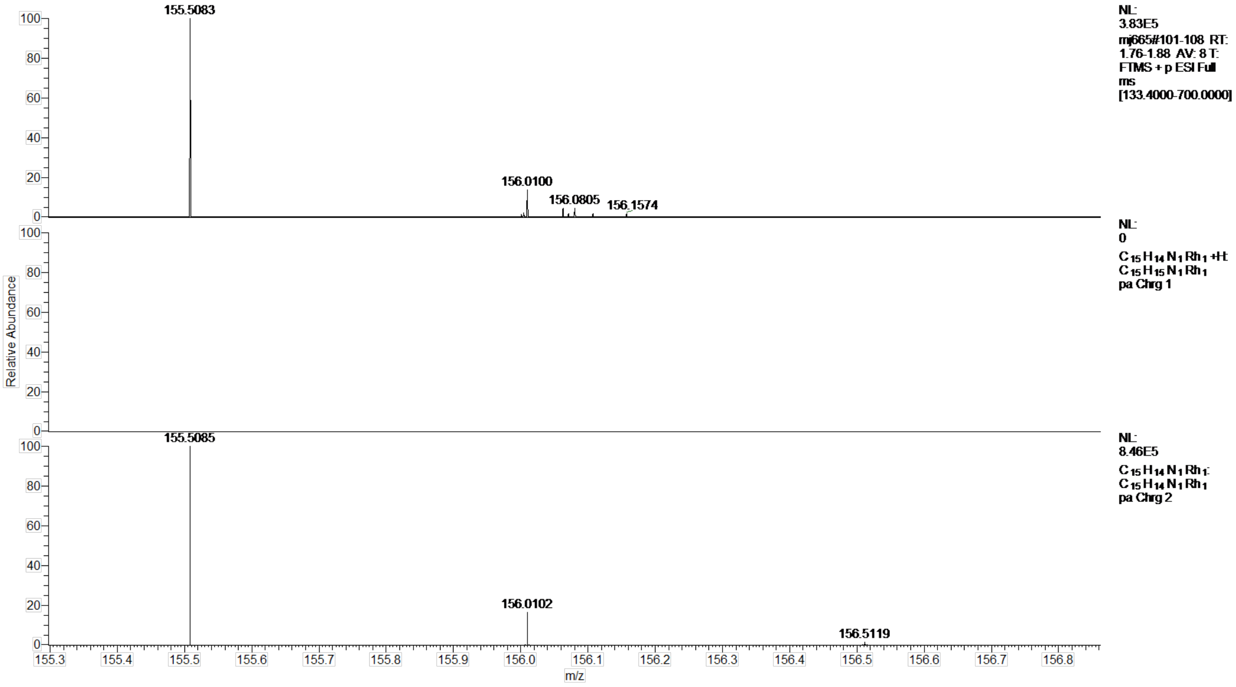

*Figure S4: MS (ESI pos) of **2a**.*

1.2 Aminorhodocenium hexafluoridophosphate (3)

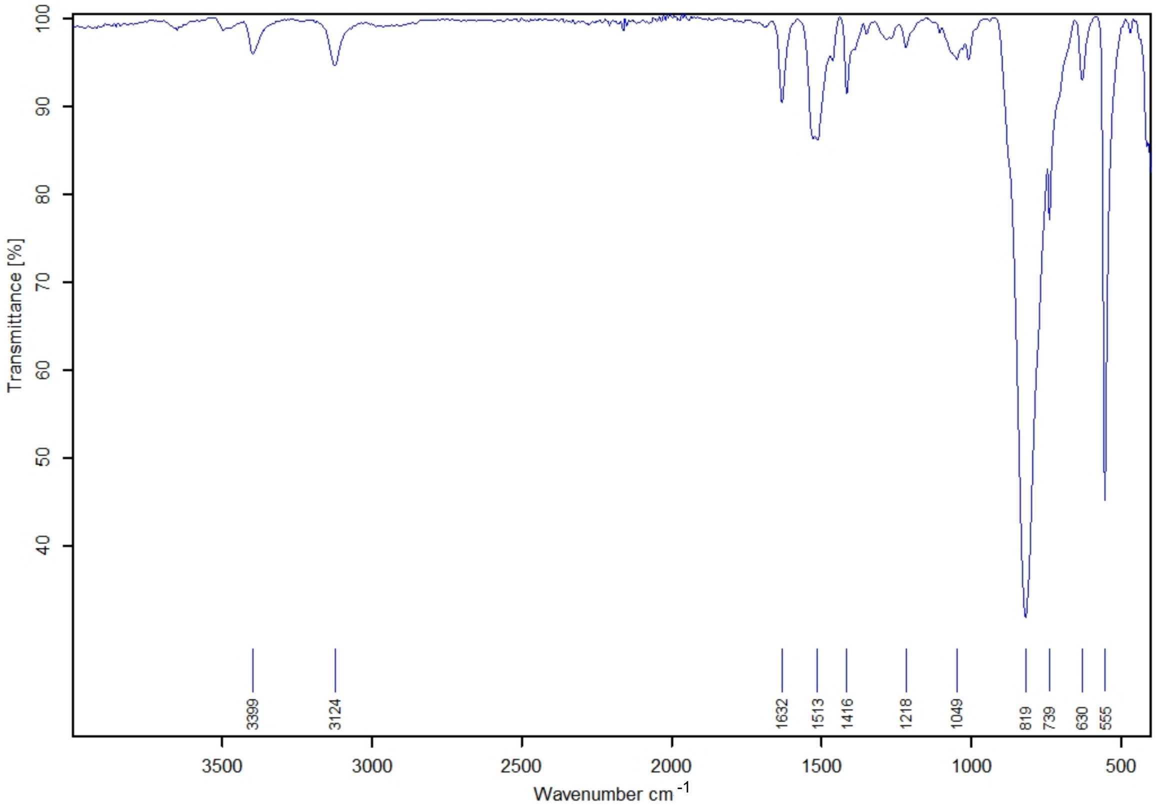

*Figure S5: ATR-IR spectrum of **3**.*

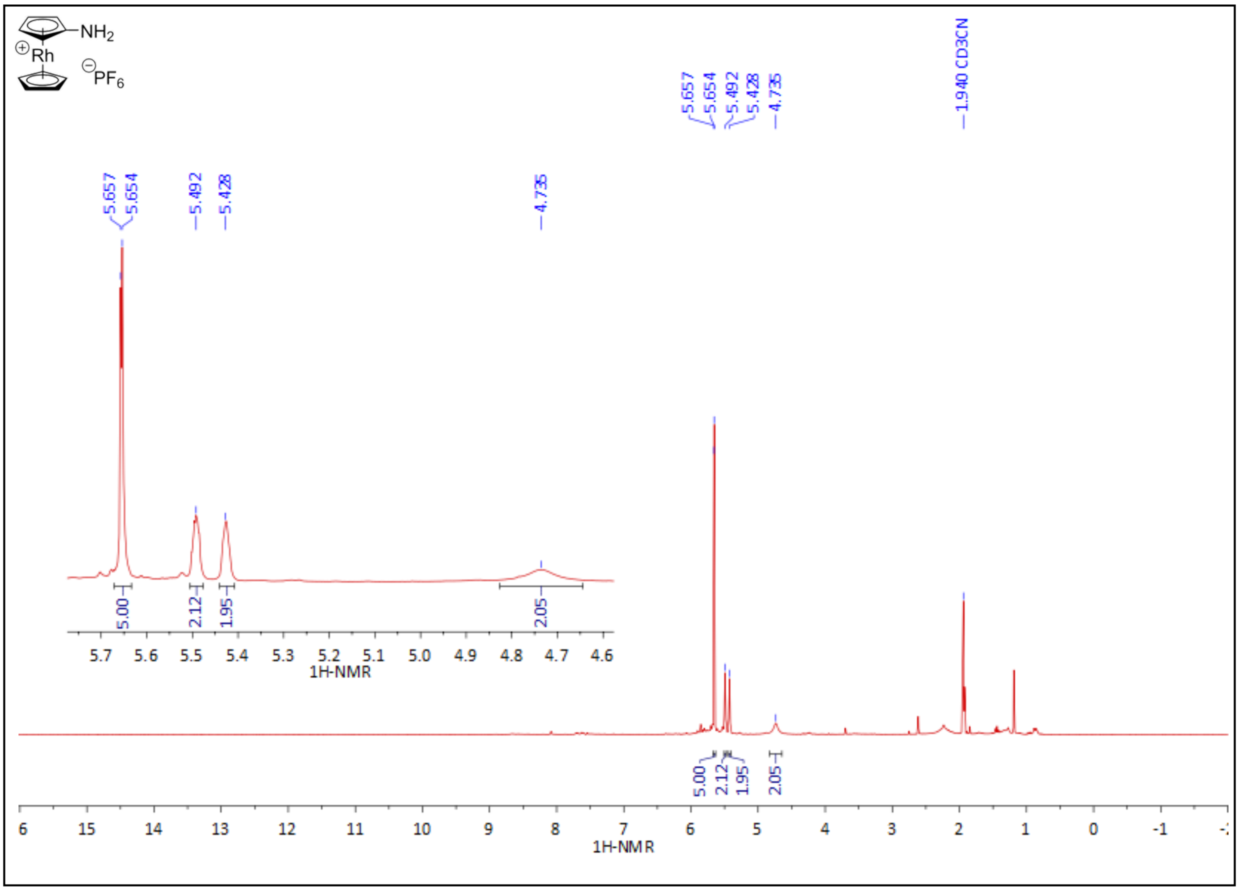

*Figure S6: ^{1}H-NMR of **3** in CD_3CN.*

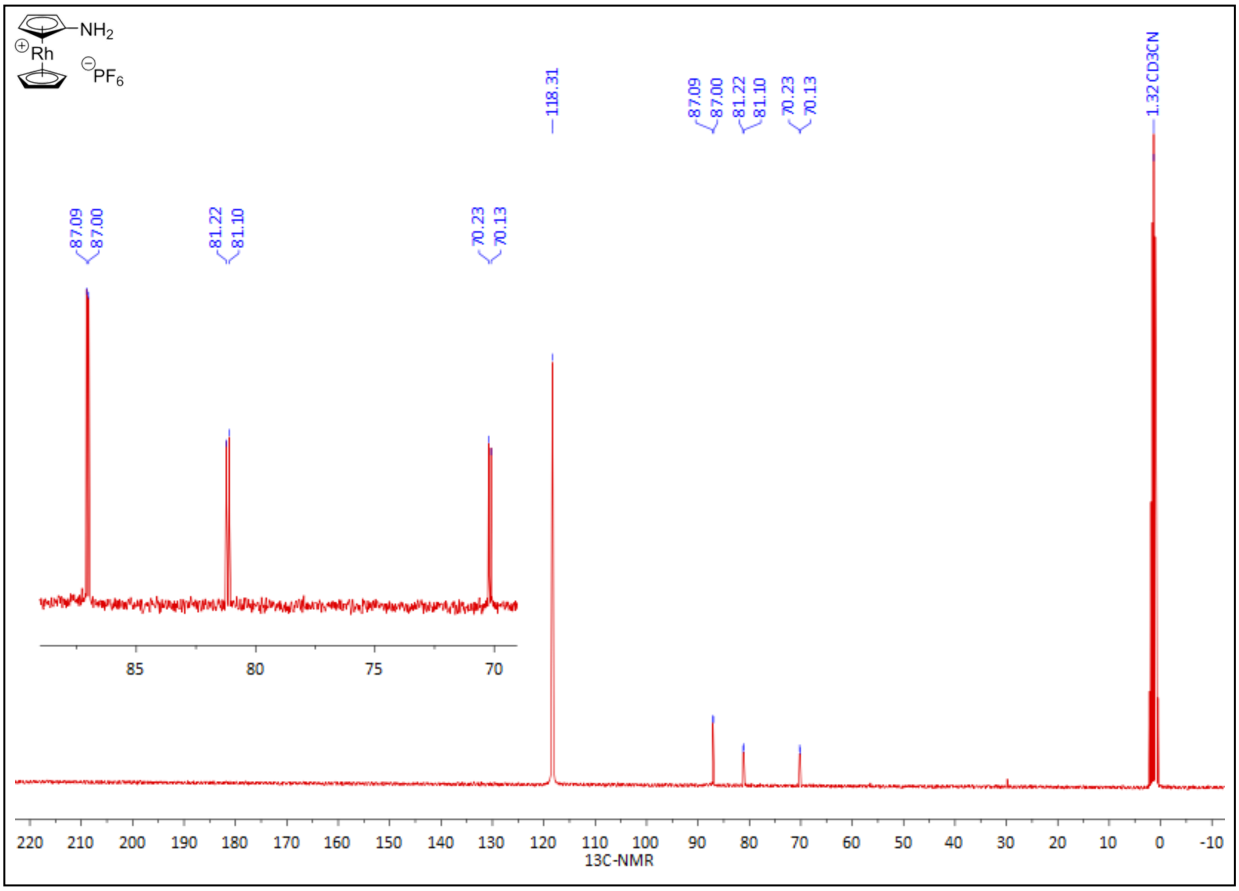

Figure S7: ^{13}C-NMR of **3** in CD_3CN.

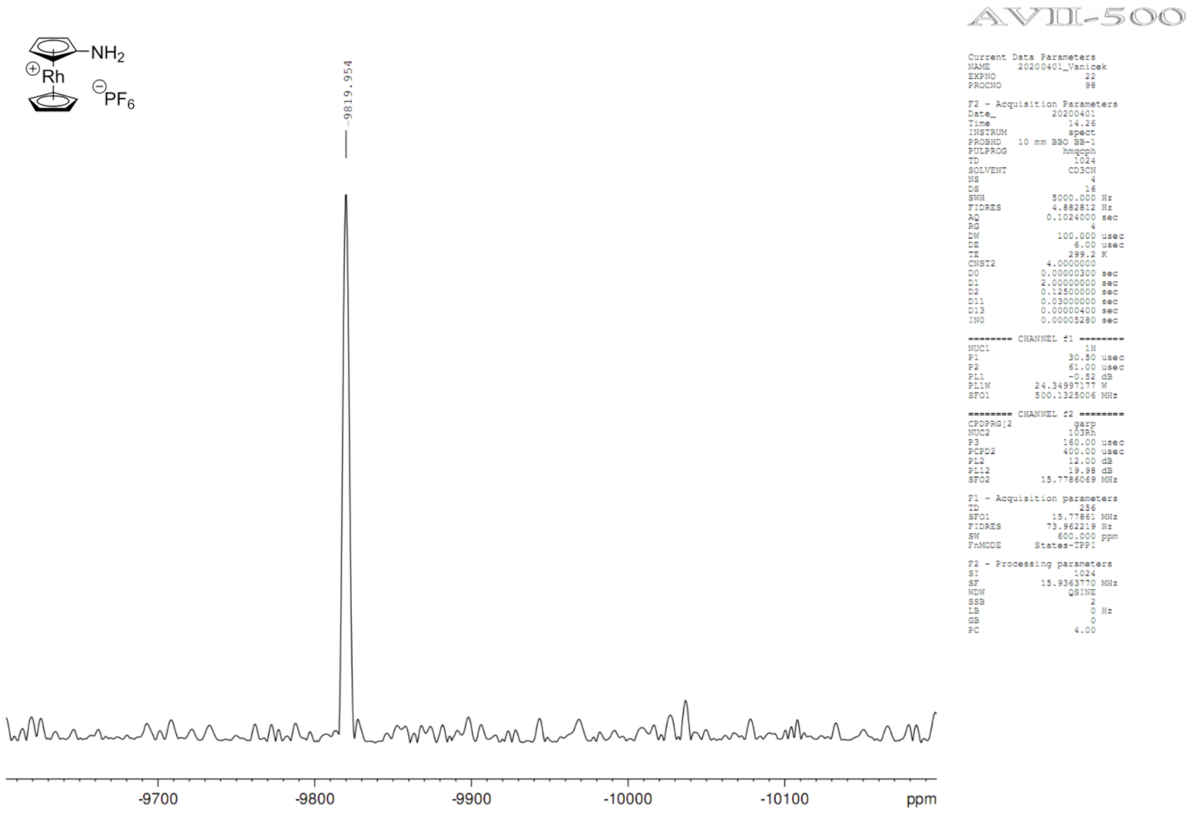

Figure S8: 1D ^{1}H,^{103}Rh-HMQC-NMR of **3** in CD_3CN.

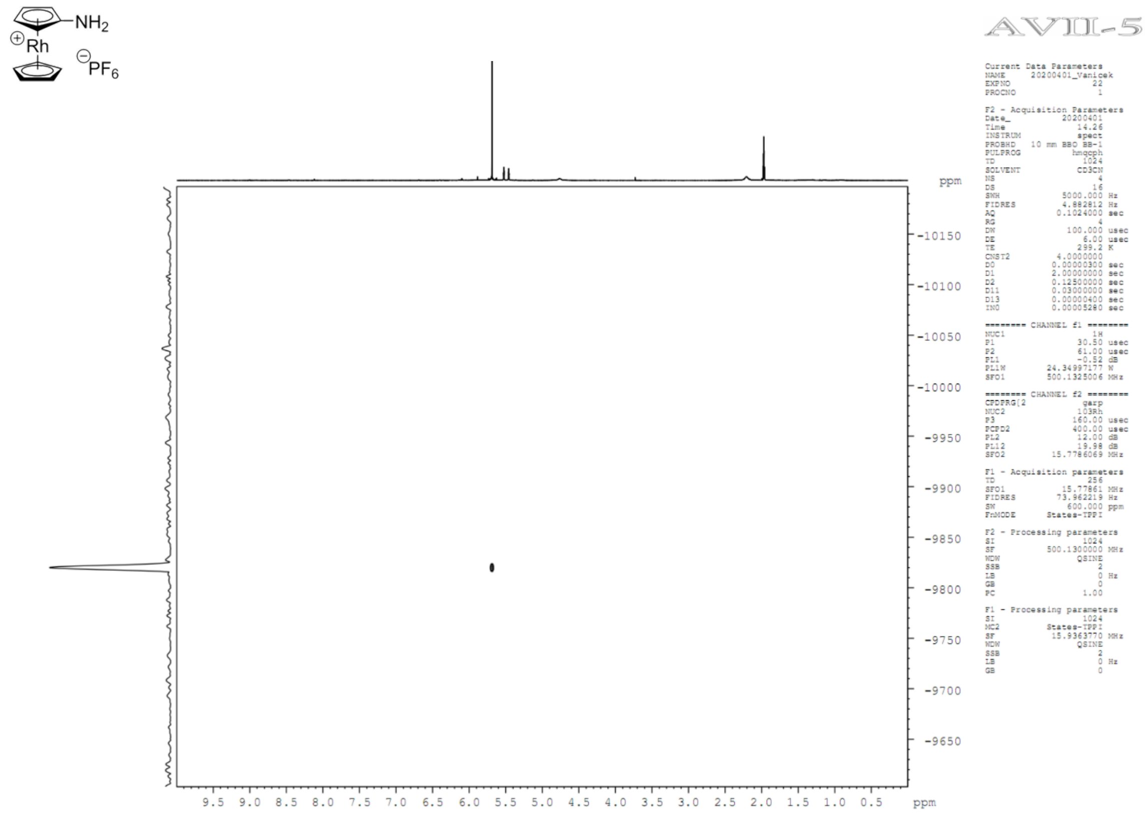

*Figure S9: 2D $^{1}H,^{103}Rh$-HMQC-NMR of **3** in CD_3CN.*

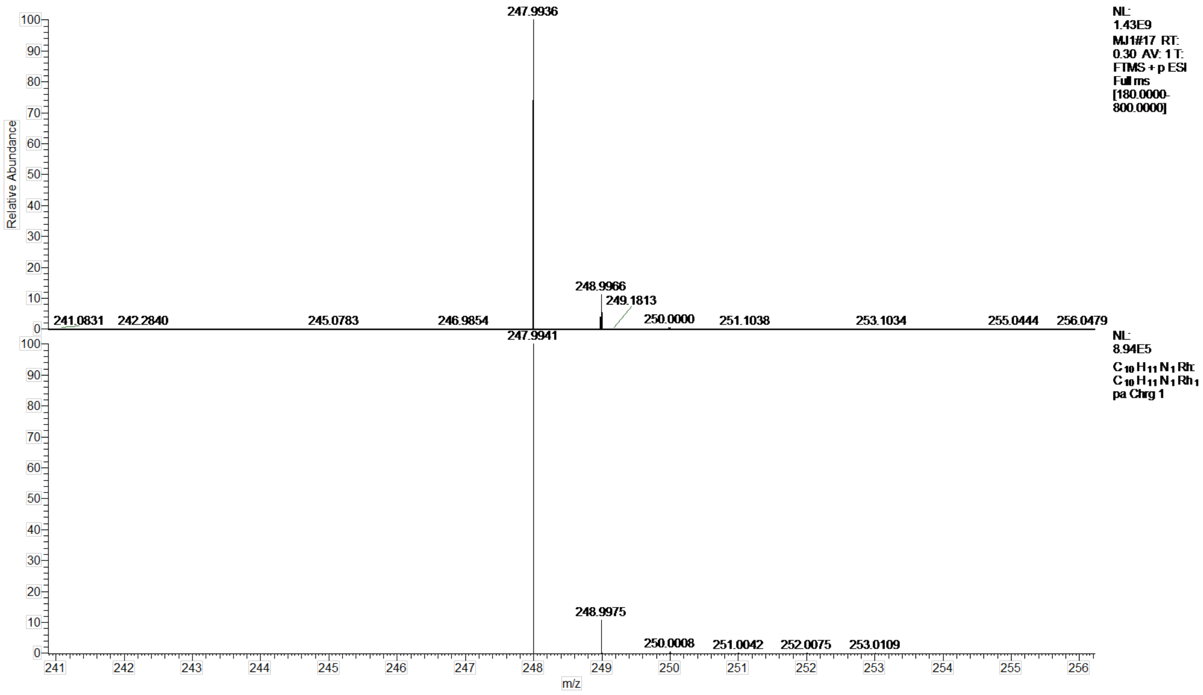

*Figure S10: MS (ESI pos) of **3**.*

1.3 Dirhodoceniumdiazene bis(hexafluoridophosphate) (4)

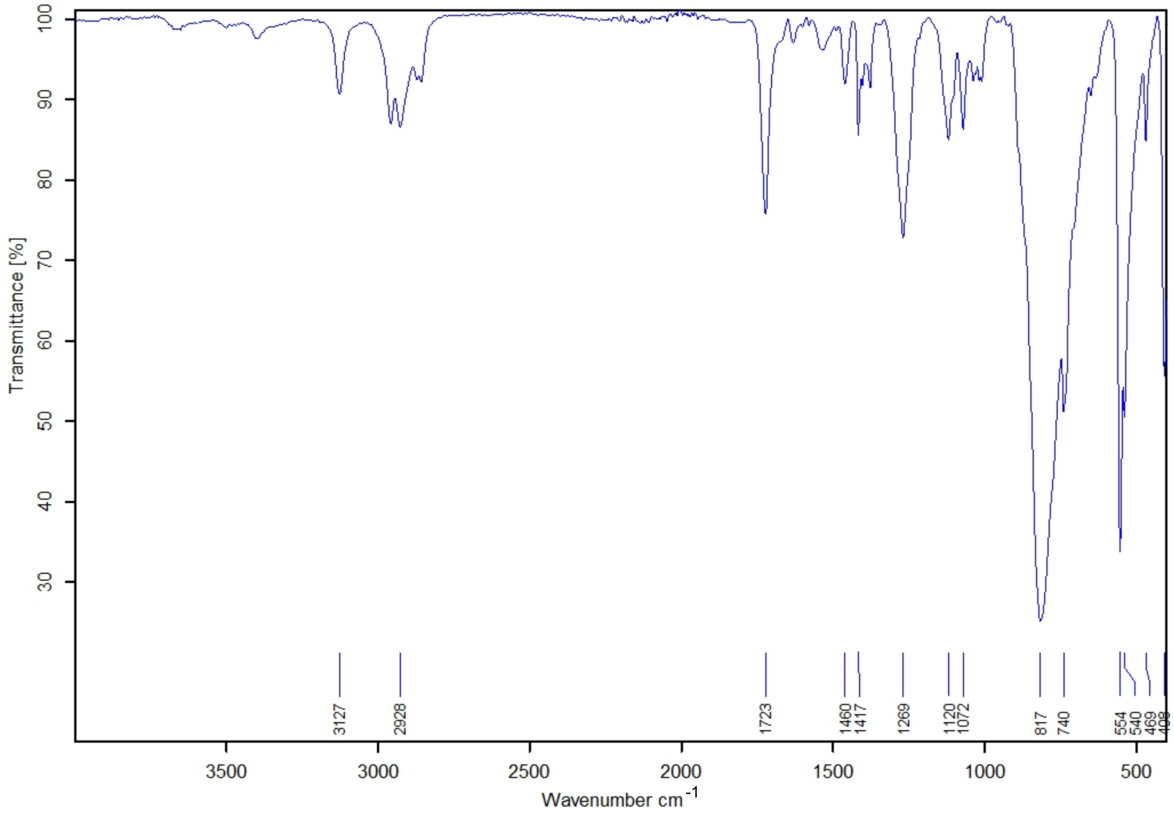

*Figure S11: ATR-IR spectrum of **4**.*

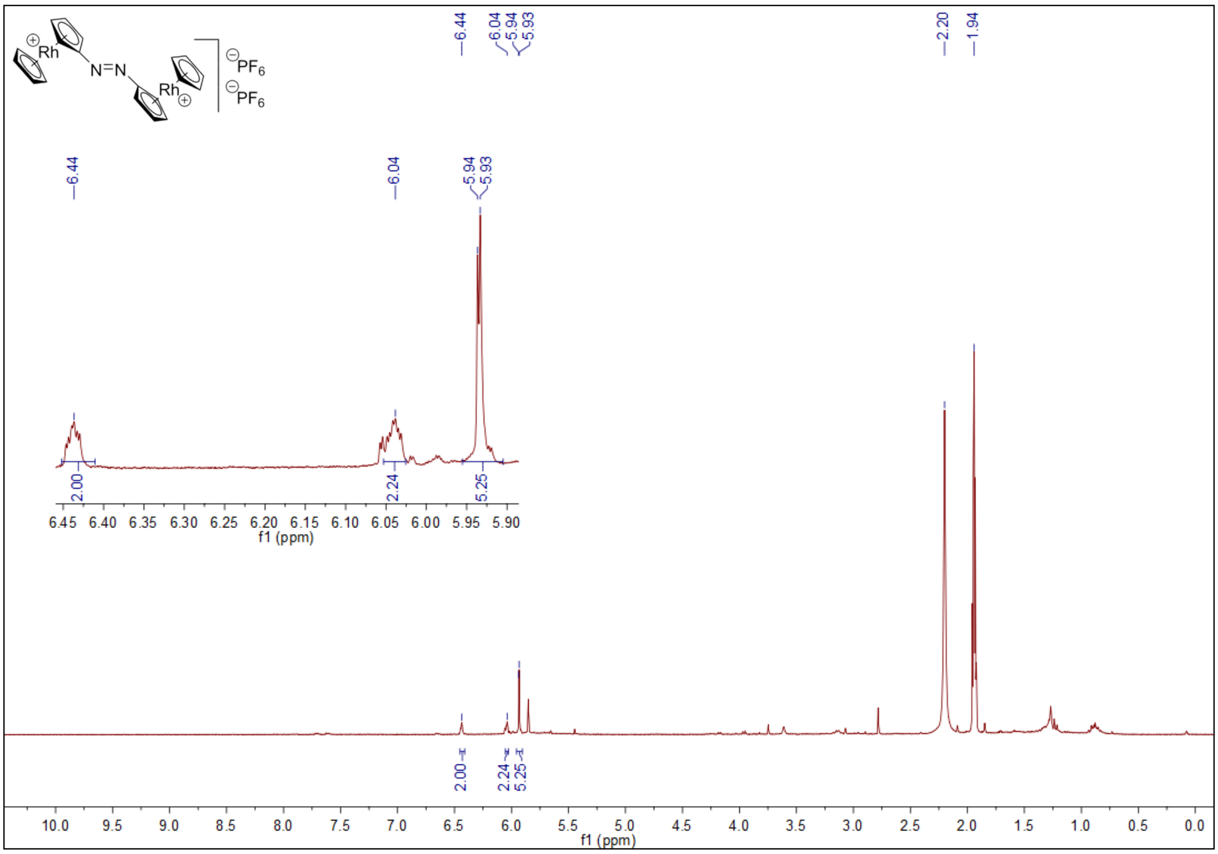

*Figure S12: ^{1}H-NMR of **4** in CD_3CN.*

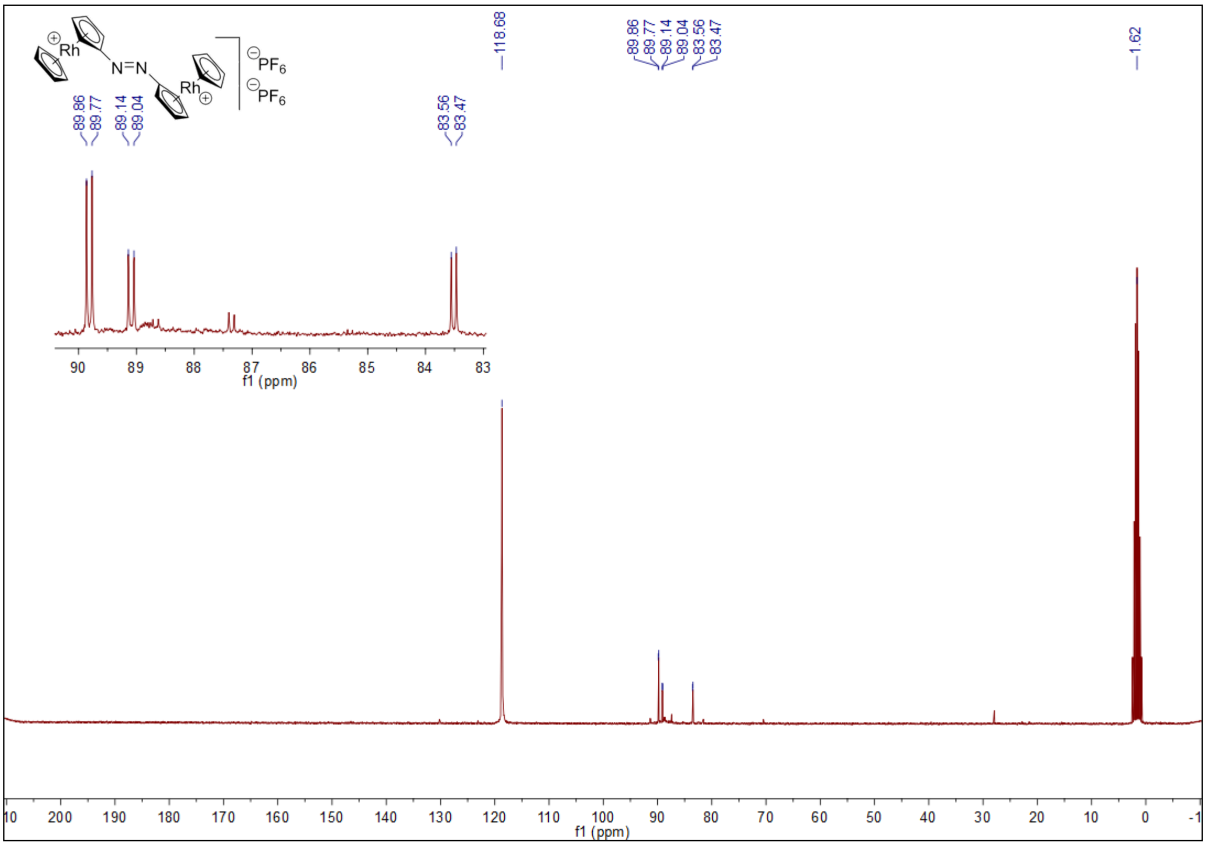

*Figure S13: ^{13}C-NMR of **4** in CD_3CN.*

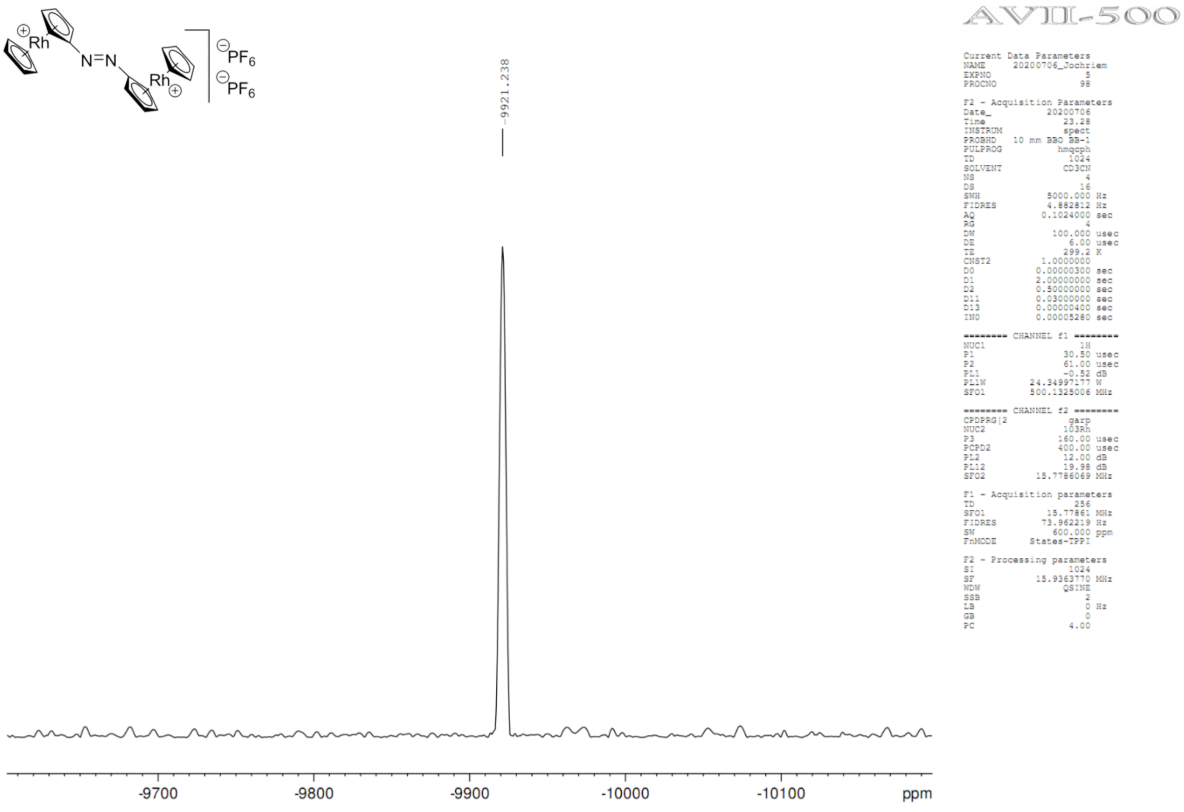

*Figure S14: 1D ^{1}H,[103]Rh-HMQC-NMR of **4** in CD_3CN.*

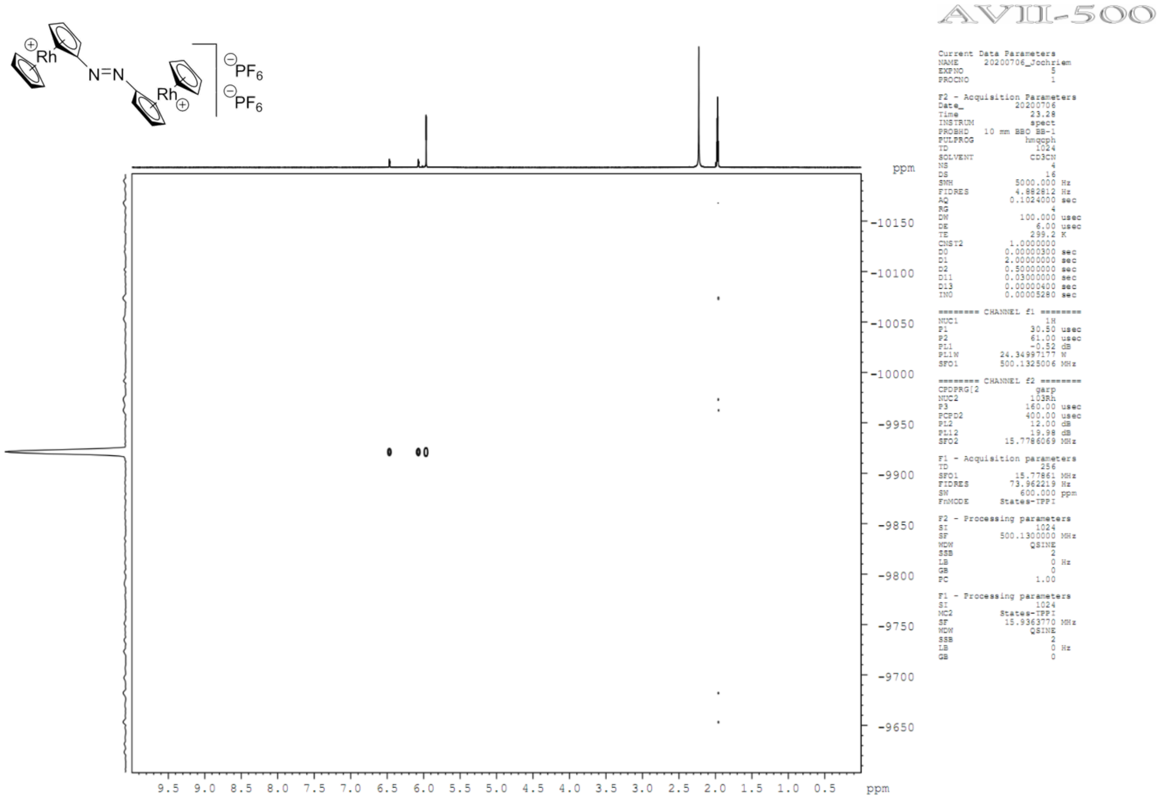

*Figure S15: 2D $^{1}H,^{103}Rh$-HMQC-NMR of **4** in CD_3CN.*

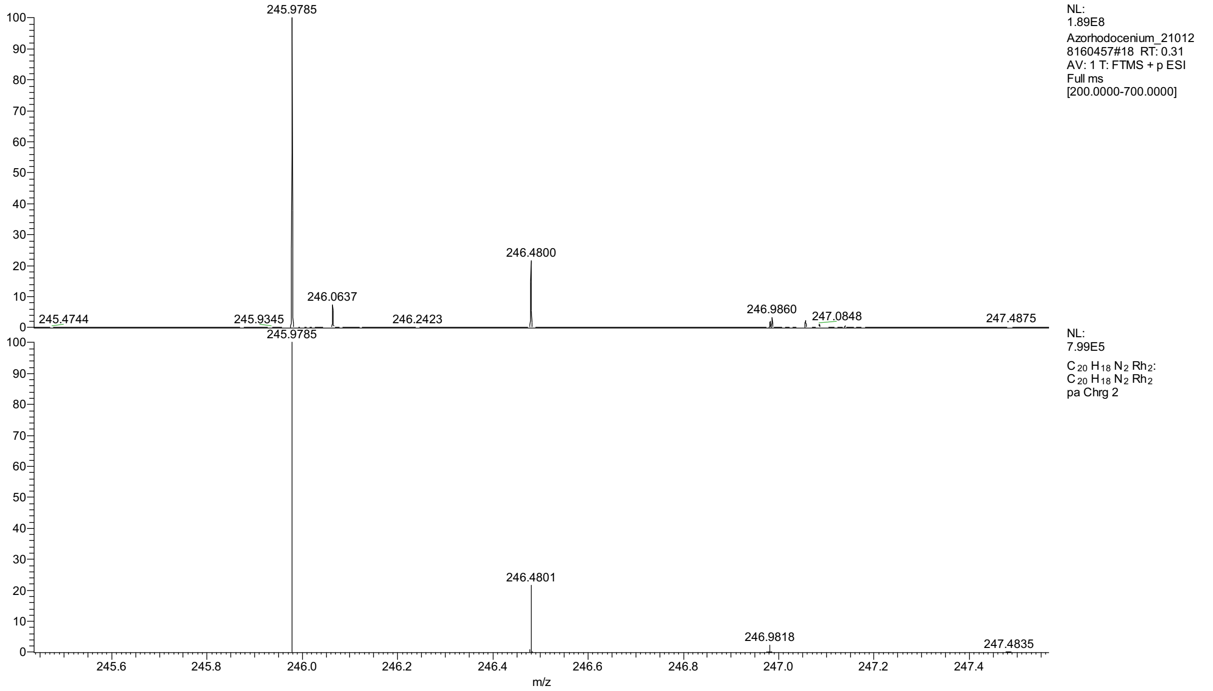

*Figure S16: MS (ESI pos) of **4**.*

1.4 Diazoniorhodocenium bis(hexafluoridophosphate) (5)

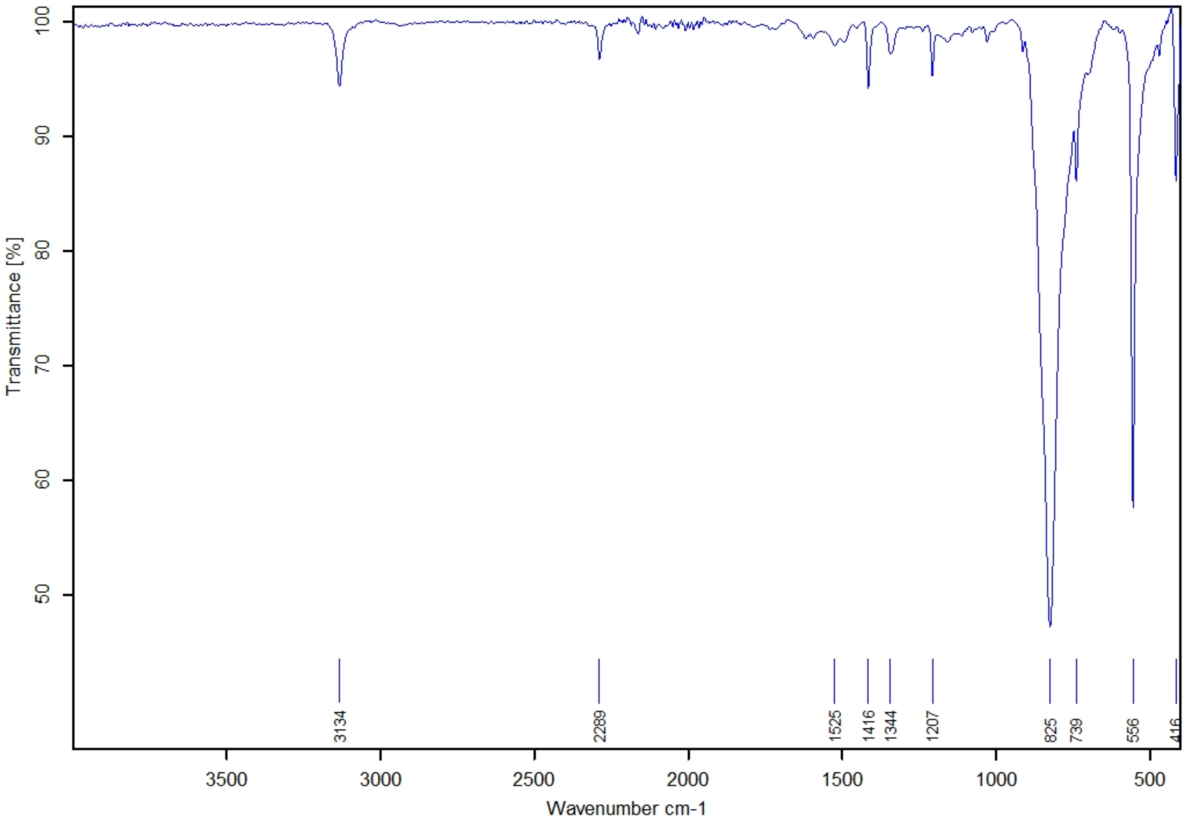

*Figure S17: ATR-IR spectrum of **5**.*

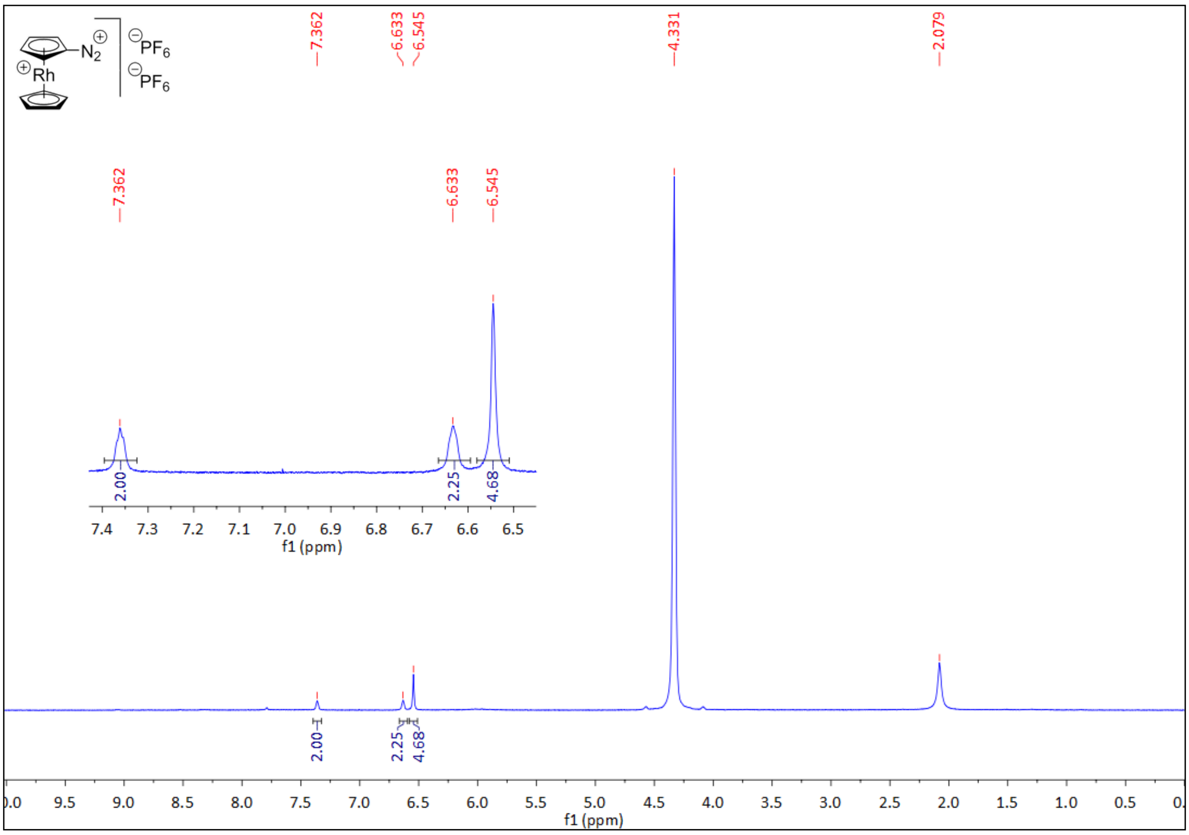

*Figure S8: ^{1}H-NMR of **5** in CD_3NO_2.*

1.5 Bromorhodocenium hexafluoridophosphate (6)

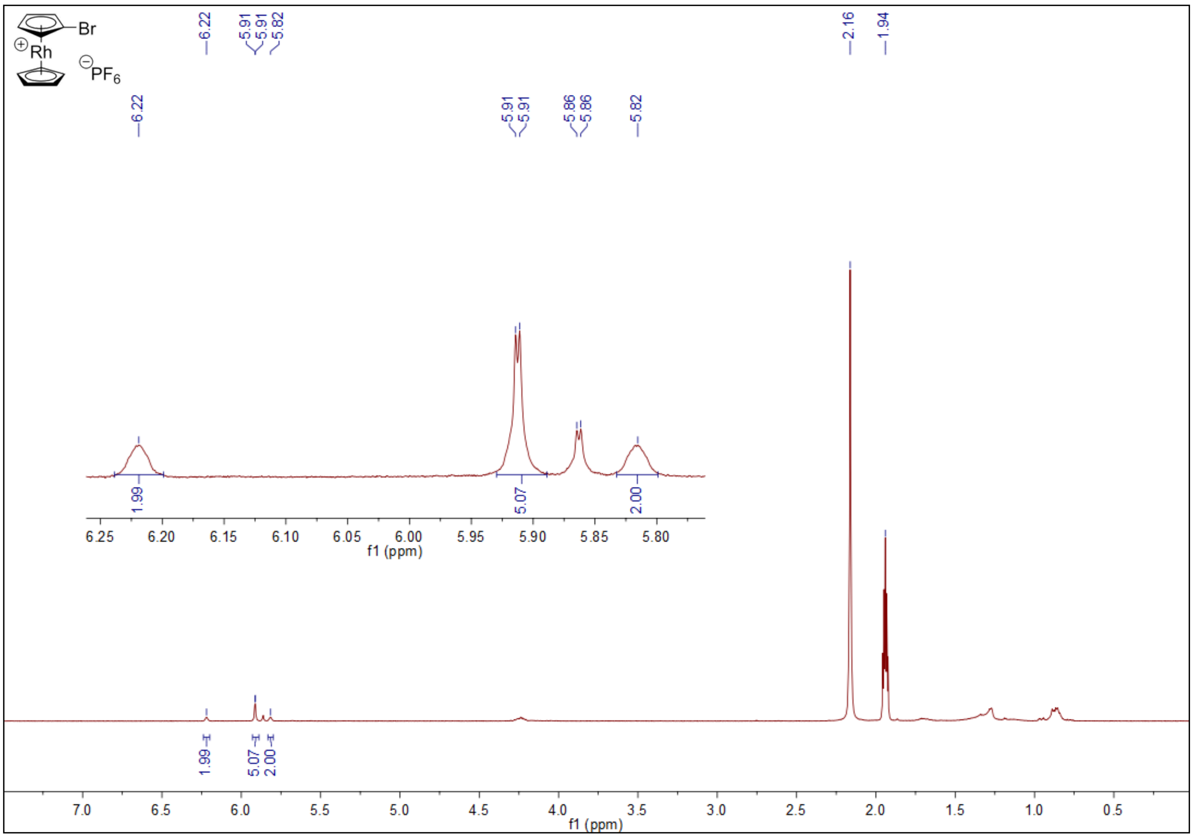

*Figure S19: ^{1}H-NMR of **6** in CD_3CN*

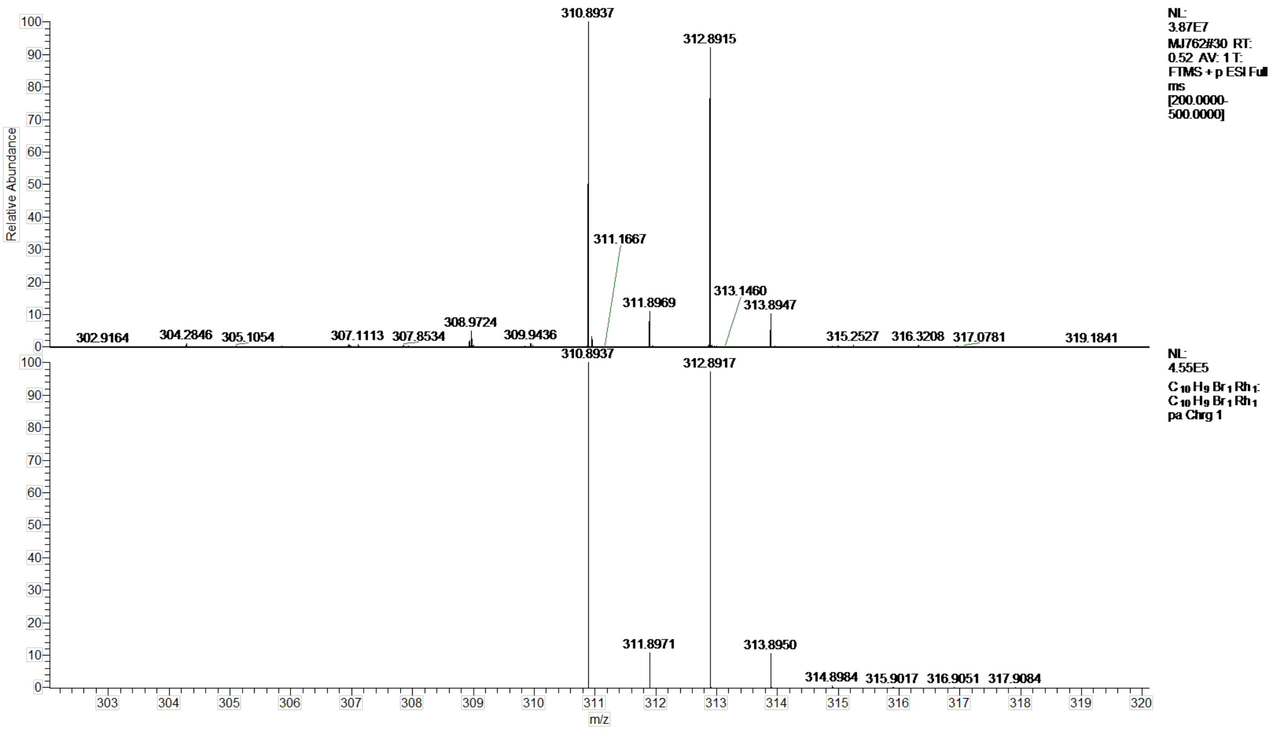

*Figure S20: MS (ESI pos) of **6**.*

1.6 Azidorhodocenium hexafluoridophosphate (7)

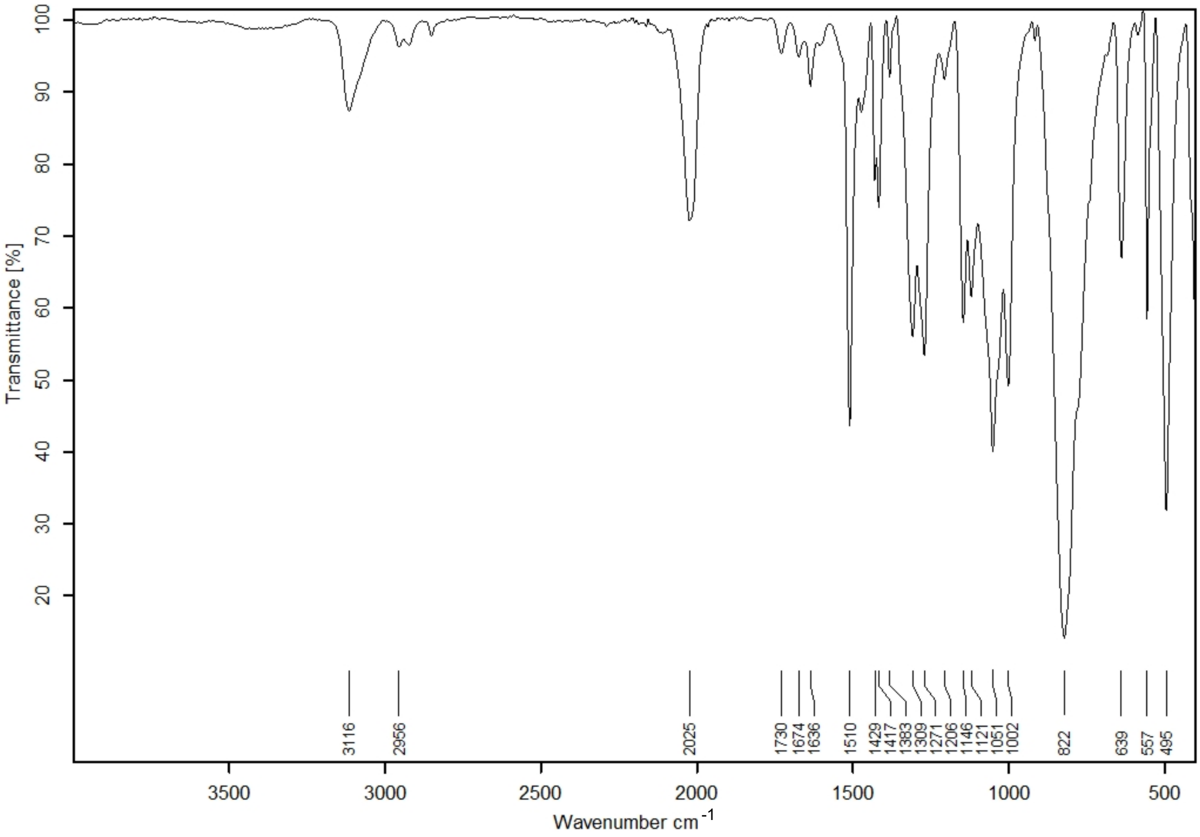

*Figure S21: ATR-IR spectrum of **7**.*

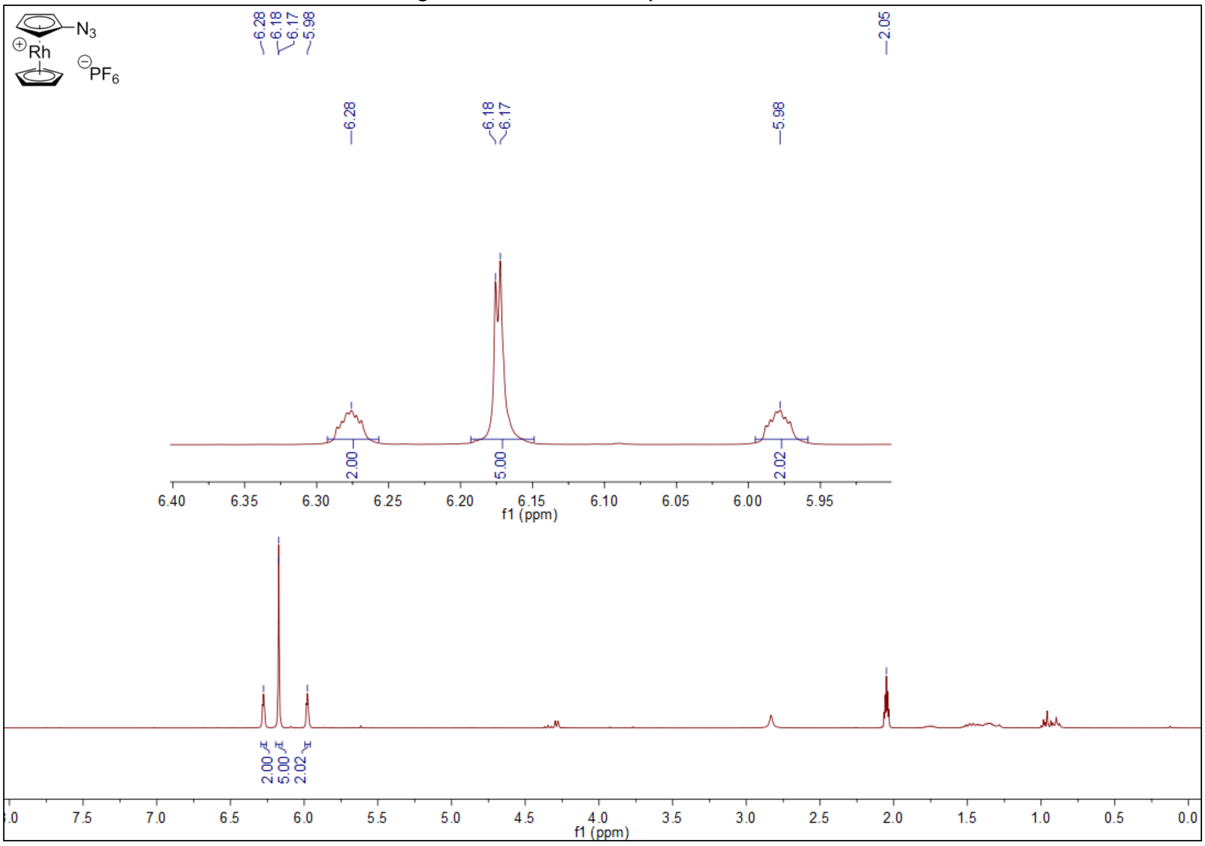

*Figure S22: ^{1}H-NMR of **7** in acetone-d6.*

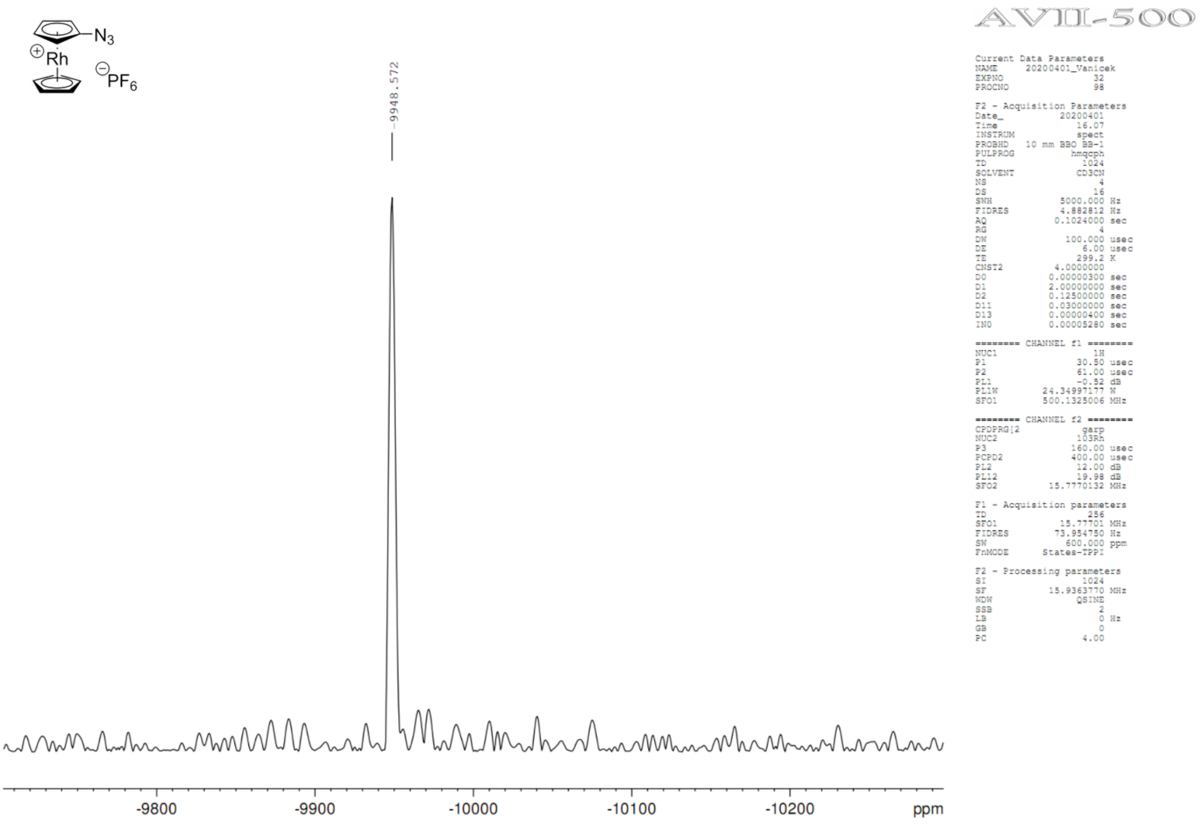

*Figure S23: 1D $^{1}H,^{103}Rh$-HMQC-NMR of **7** in CD_3CN.*

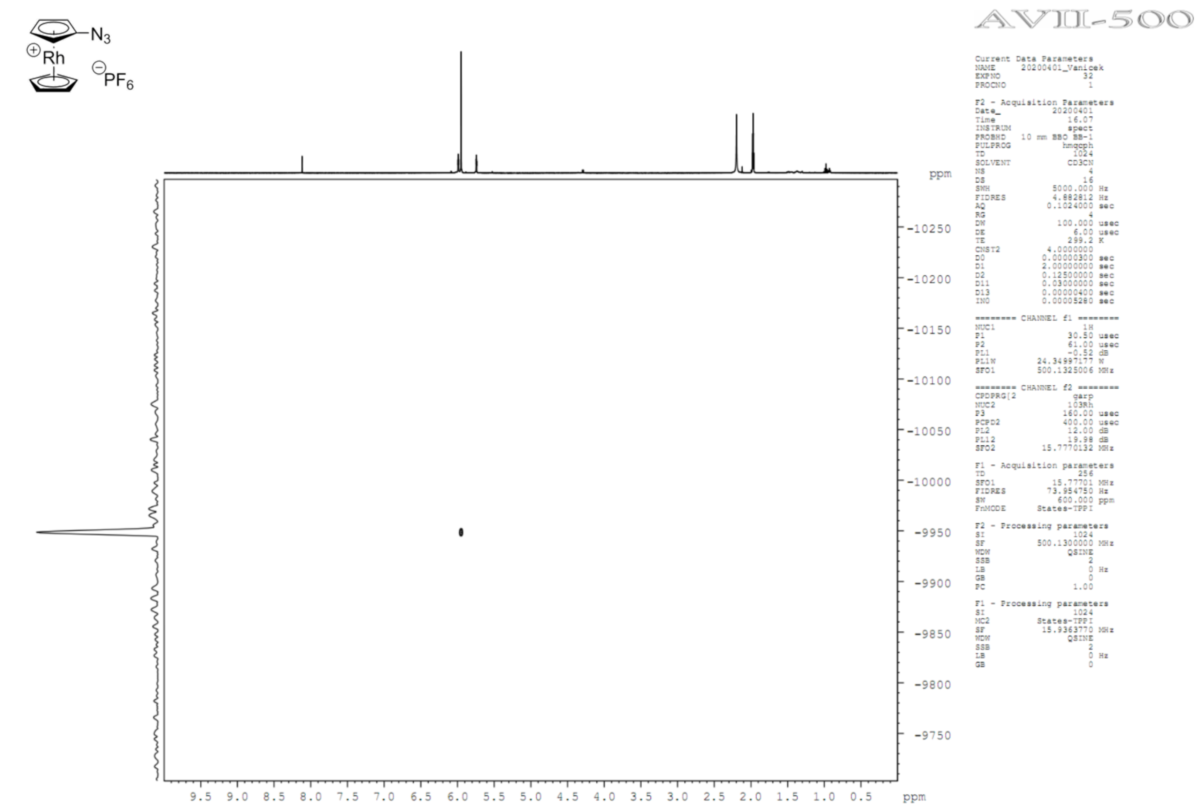

*Figure S24: 2D $^{1}H,^{103}Rh$-HMQC-NMR of **7** in CD_3CN.*

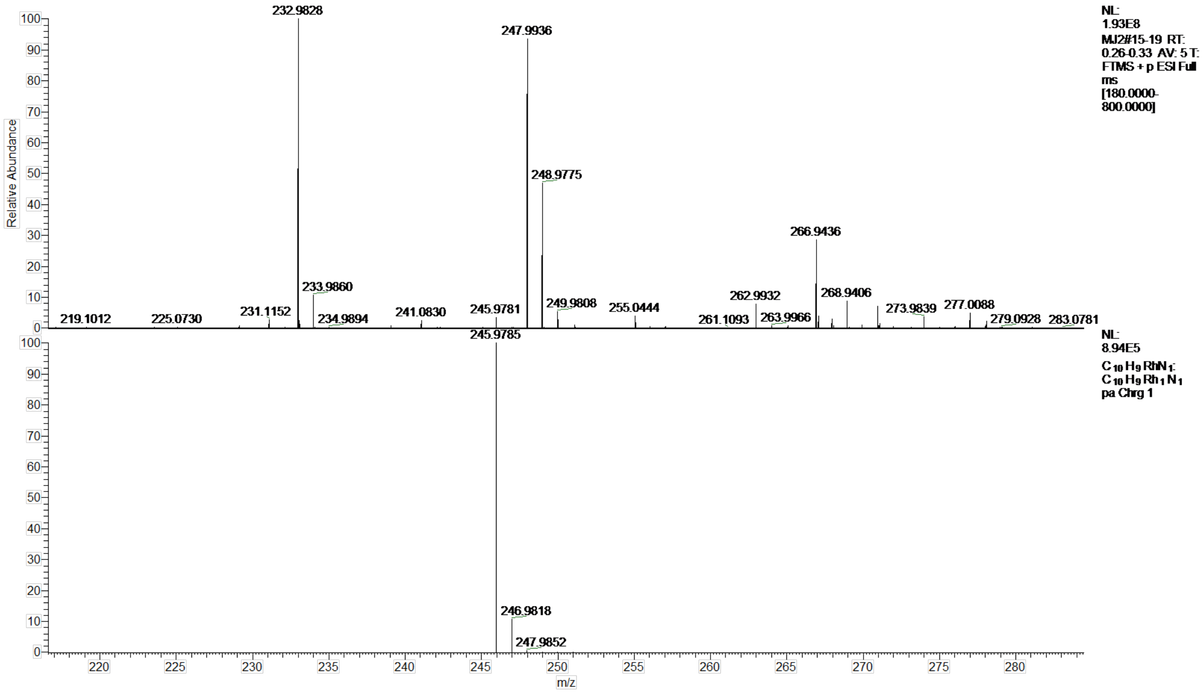

Figure S25: MS (ESI pos) of **7**.

1.6 Iodorhodocenium hexafluoridophosphate (8a)

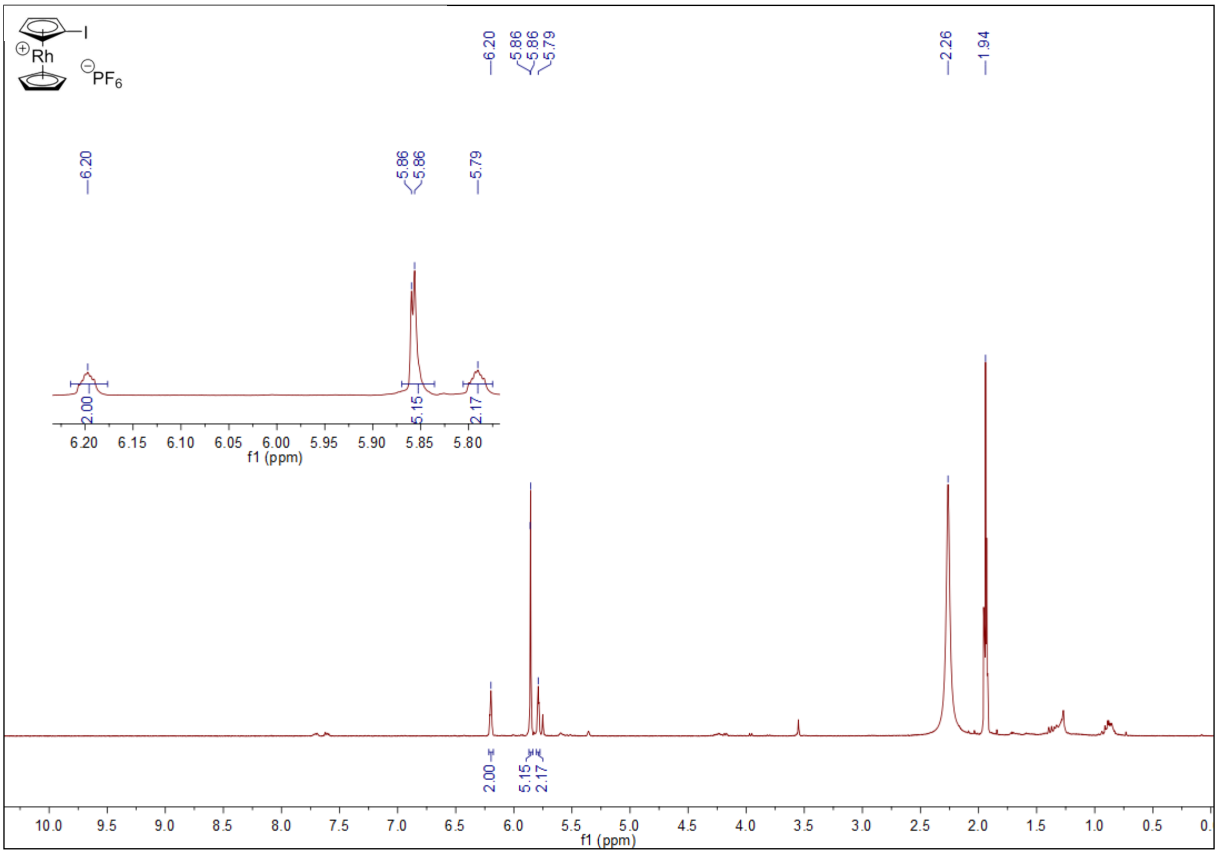

*Figure S26: ^{1}H-NMR of **8a** in CD_3CN.*

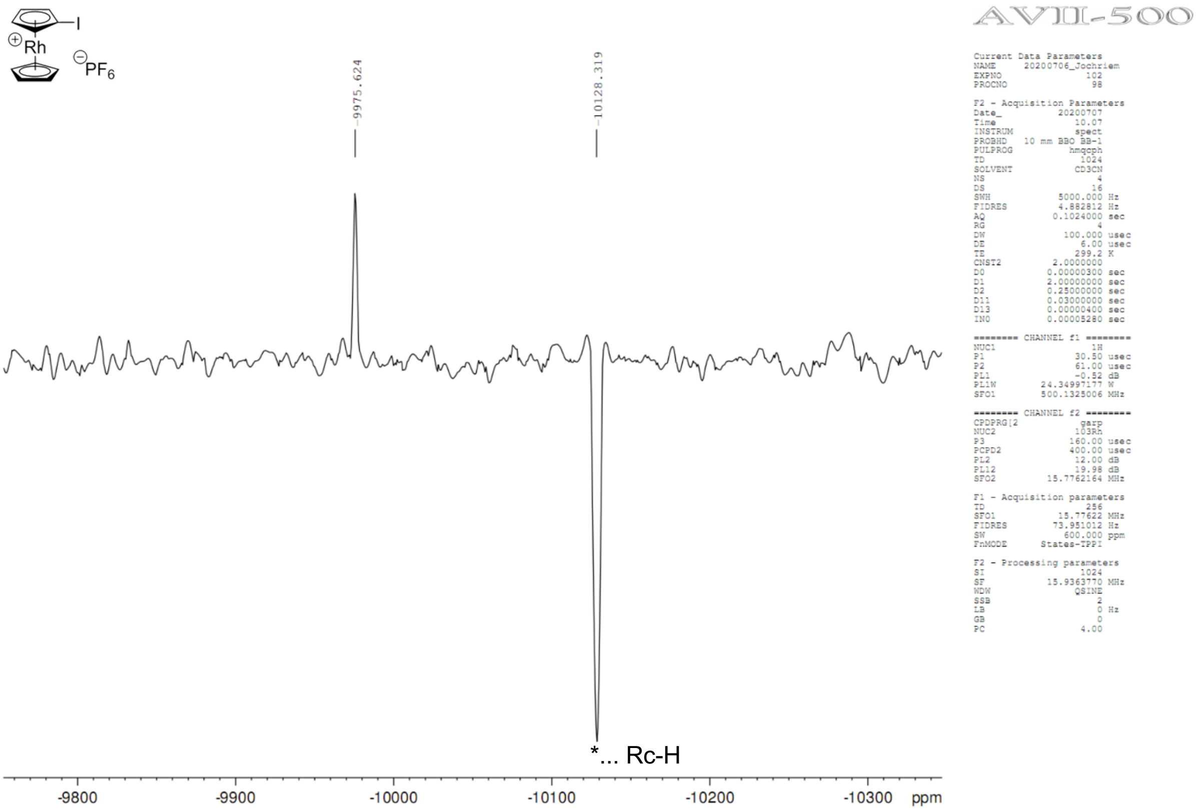

*Figure S27: 1D ^{1}H,^{103}Rh-HMQC-NMR of **8a** in CD_3CN.*

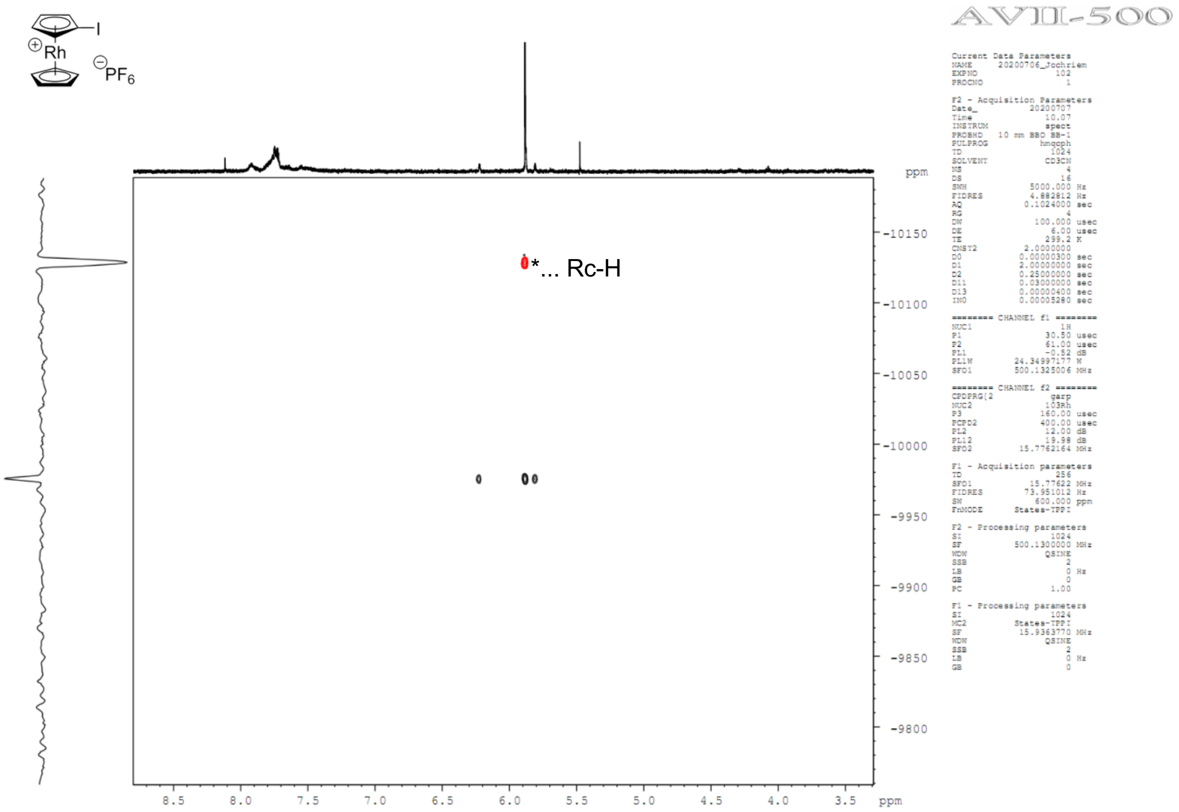

Figure S28: 2D $^{1}H,^{103}Rh$-HMQC-NMR of **8a** in CD_3CN.

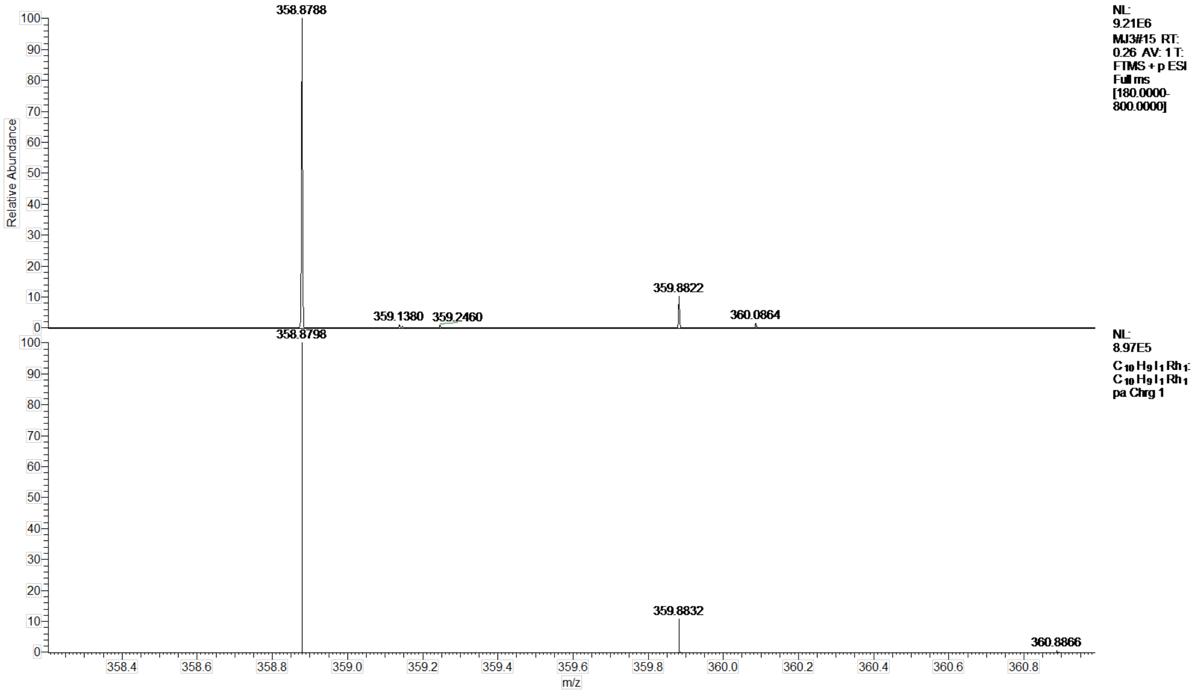

Figure S29: MS (ESI pos) of **8a**.

1.6 Iodorhodocenium iodide (8b)

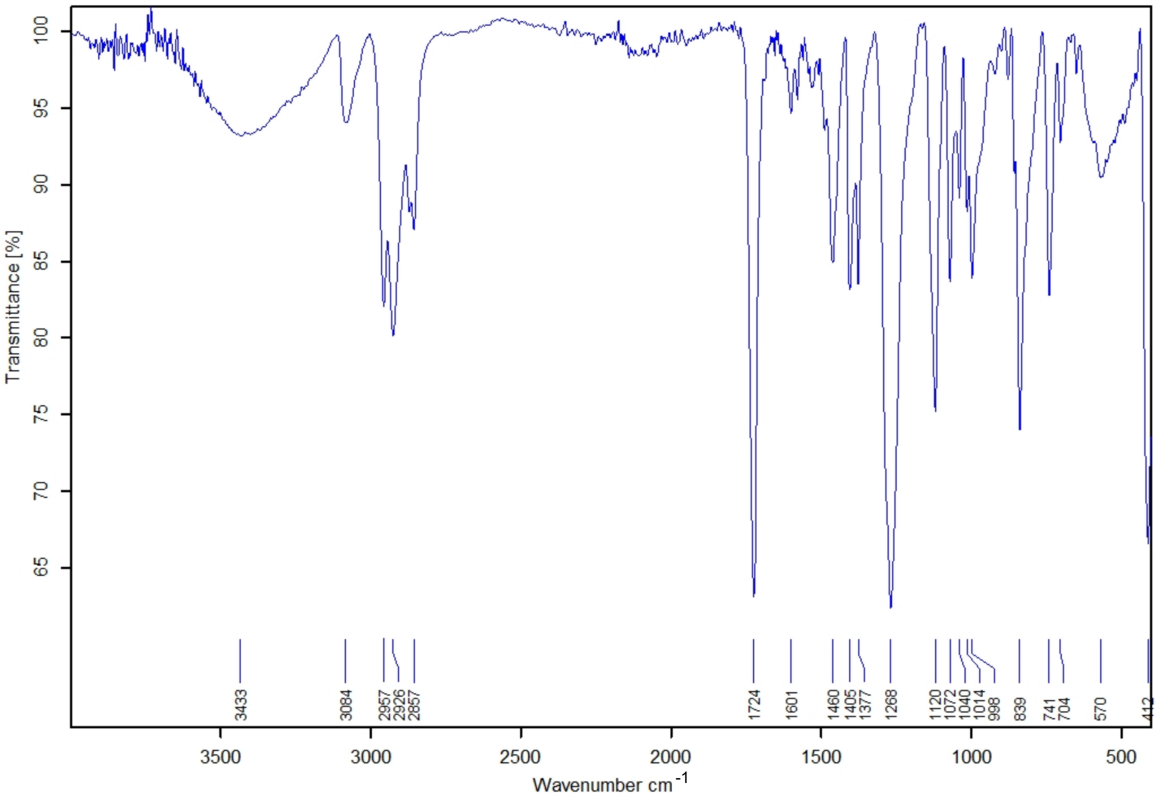

Figure S30: ATR-IR spectrum of ***8b****.*

1.7 Triphenylphosphoniorhodocenium bis(hexafluoridophosphate) (9)

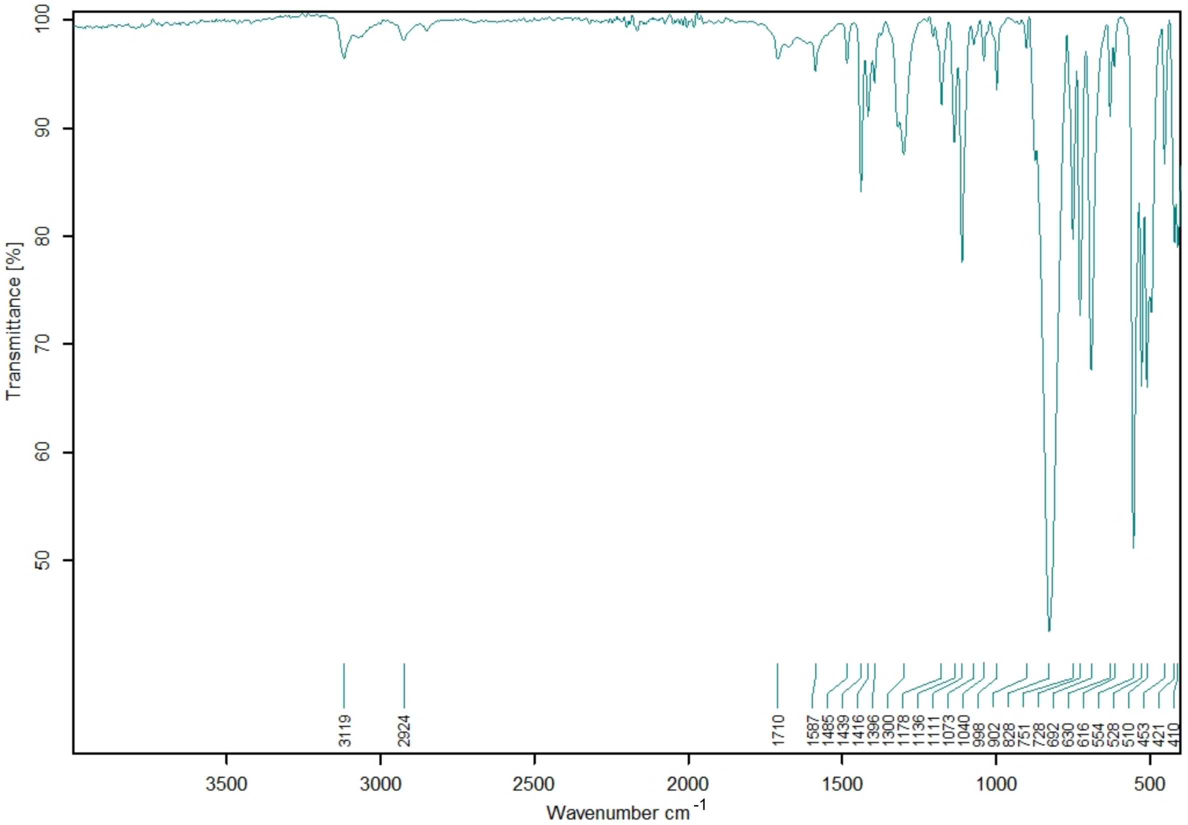

*Figure S31: ATR-IR Spectrum of **9**.*

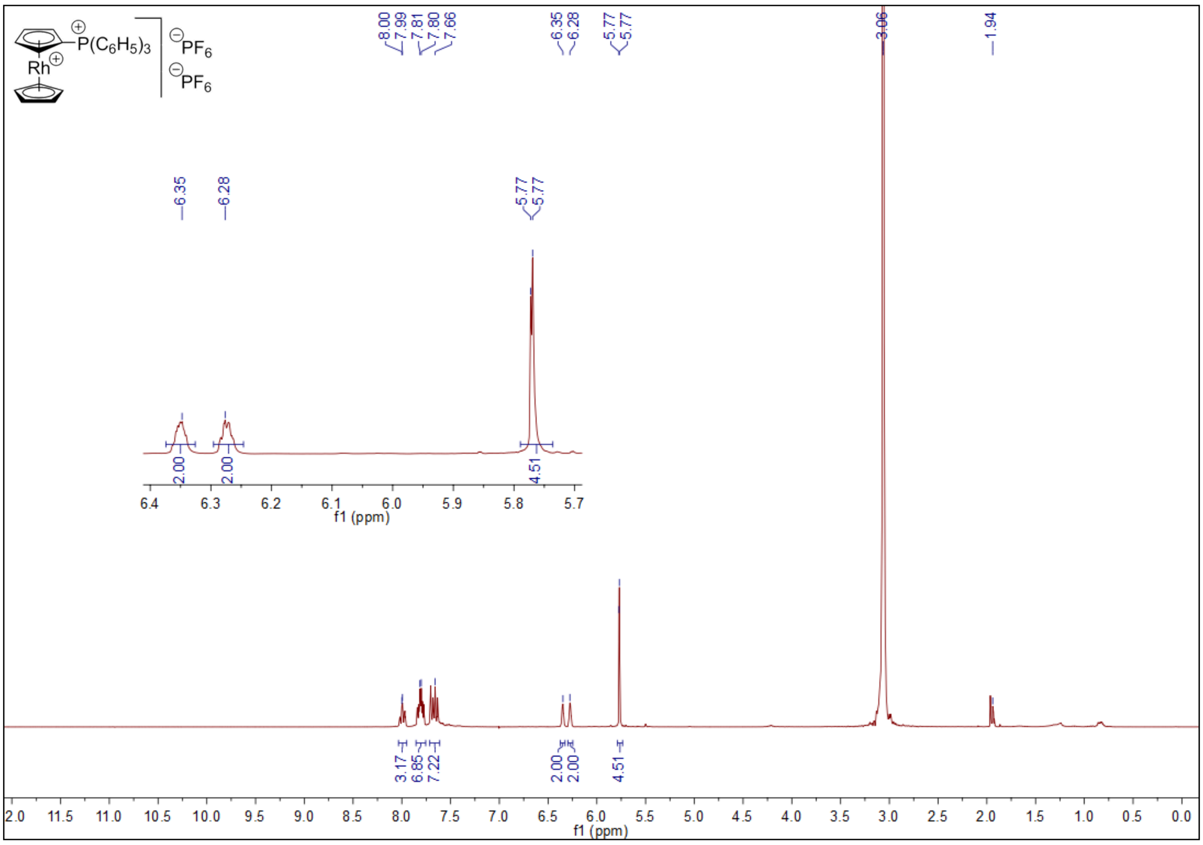

*Figure S32: ^{1}H-NMR of **9** in CD_3CN.*

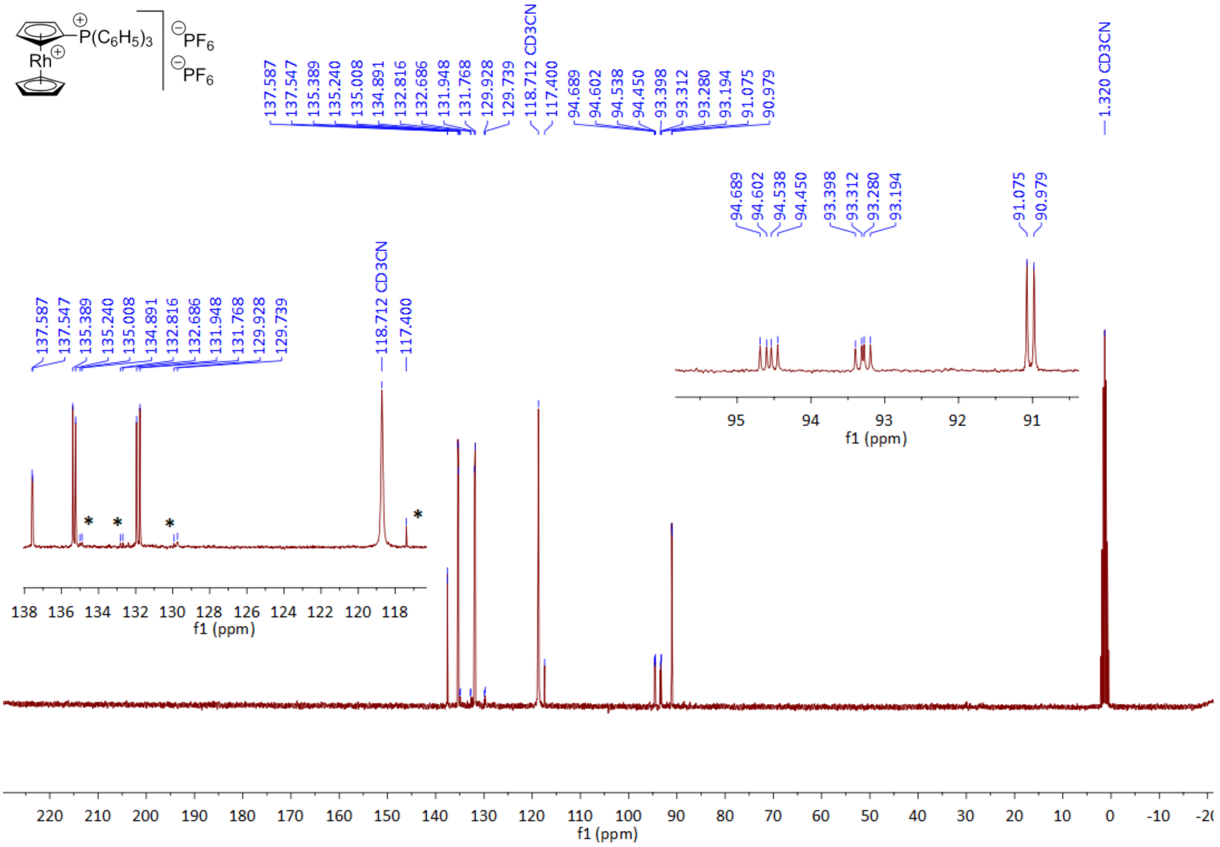

*Figure S33: ^{13}C-NMR of **9** in CD_3CN, * denotes signals of triphenylphosphine oxide impurity.*

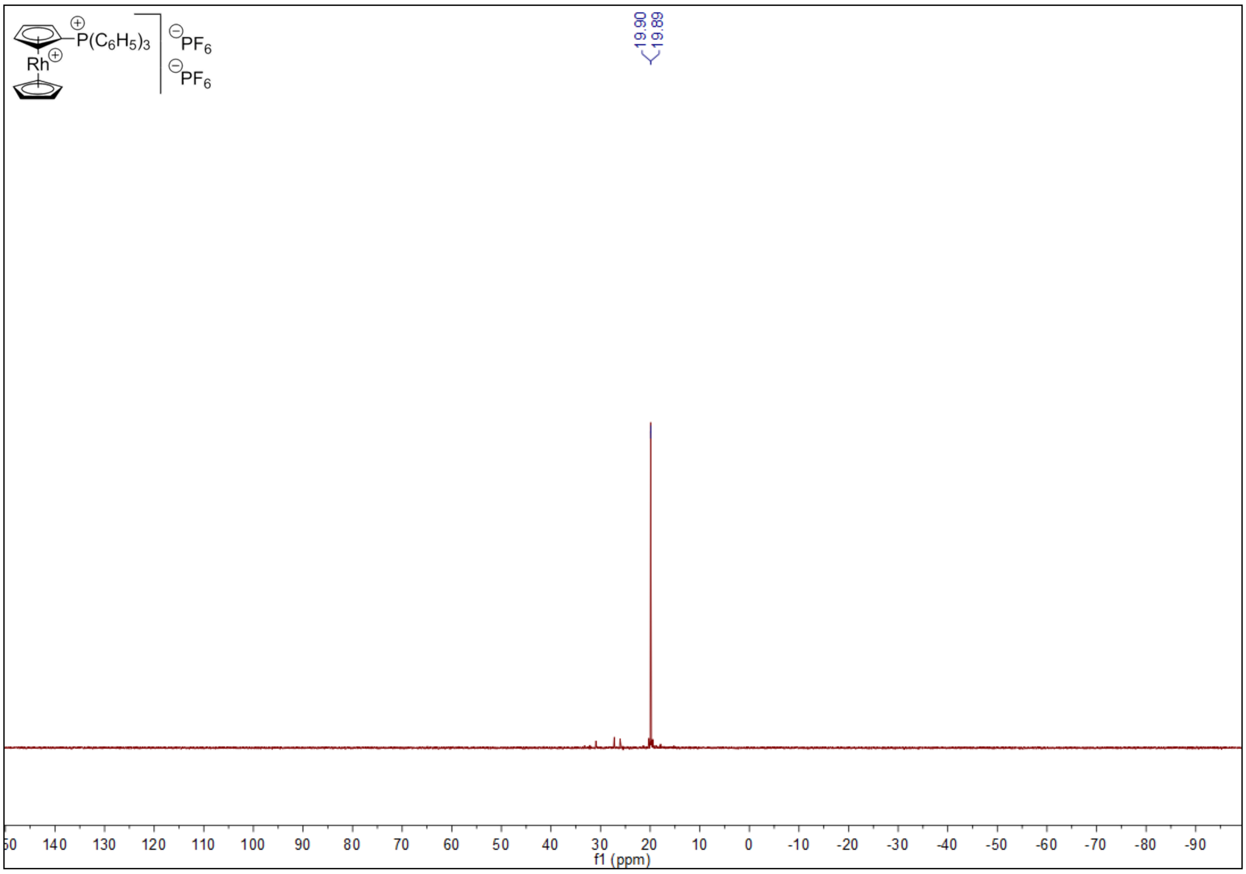

*Figure S34: ^{31}P-NMR of **9** in CD_3CN.*

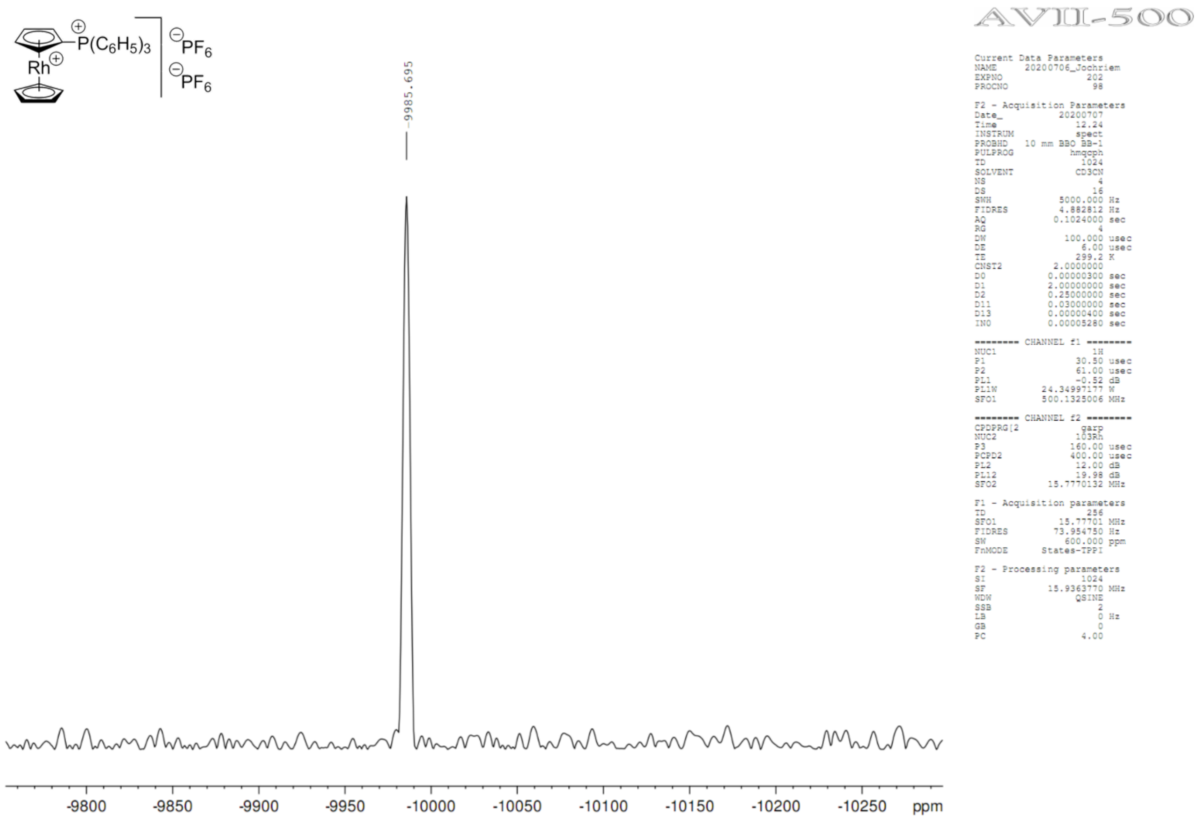

*Figure S35: 1D $^{1}H,^{103}Rh$-HMQC-NMR of **9** in CD_3CN.*

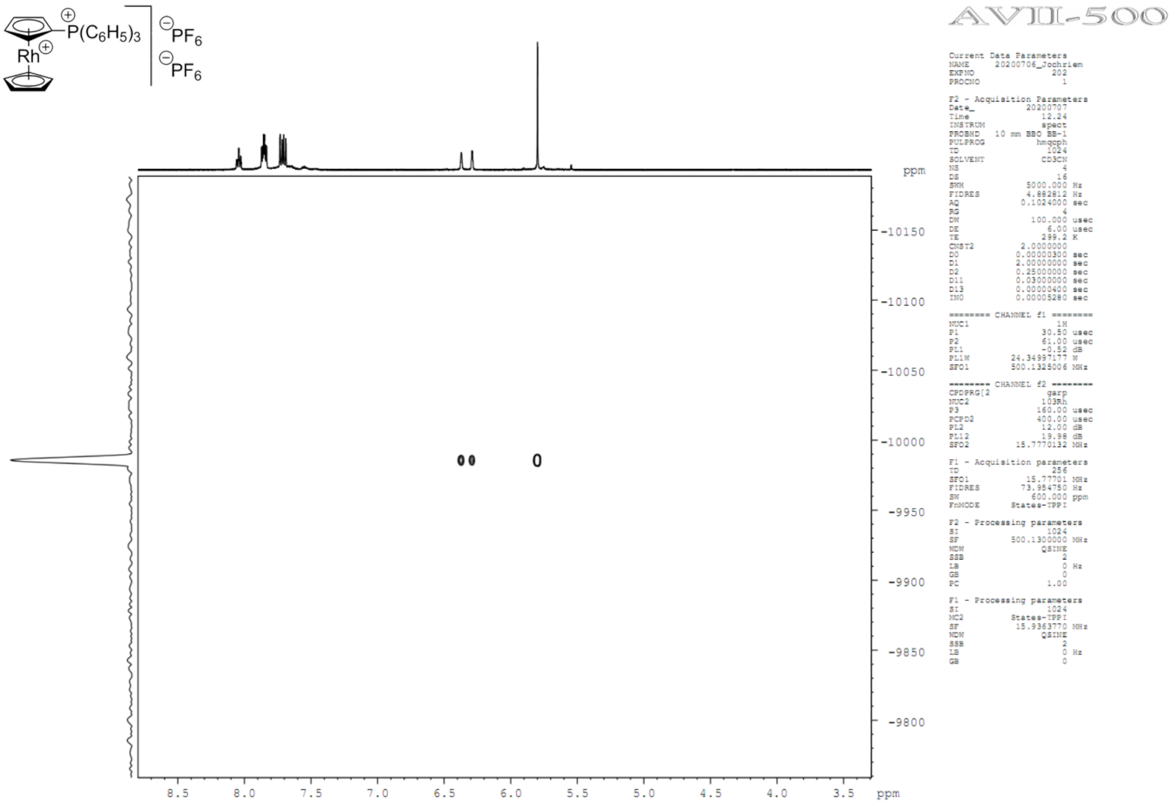

*Figure S36: 2D $^{1}H,^{103}Rh$-HMQC-NMR of **9** in CD_3CN.*

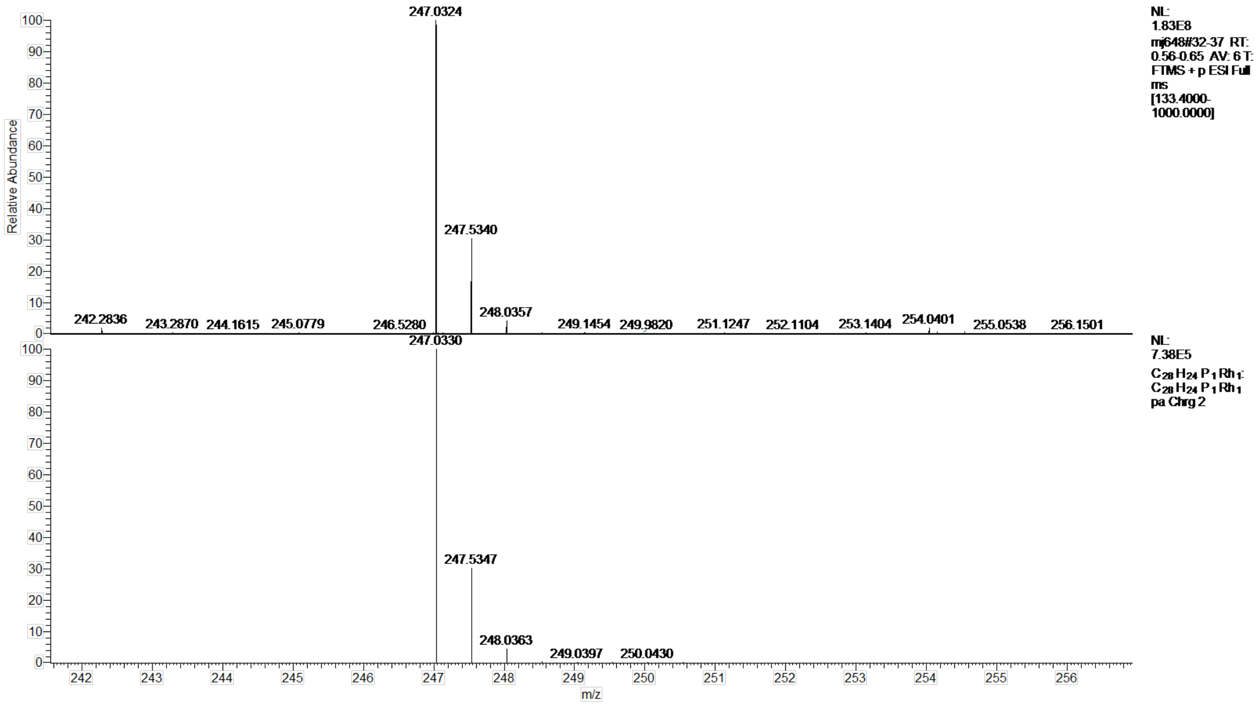

Figure S37: MS (ESI pos) of **9**.

1.8 (Triphenyl)phosphazenerhodocenium hexafluoridophosphate (10)

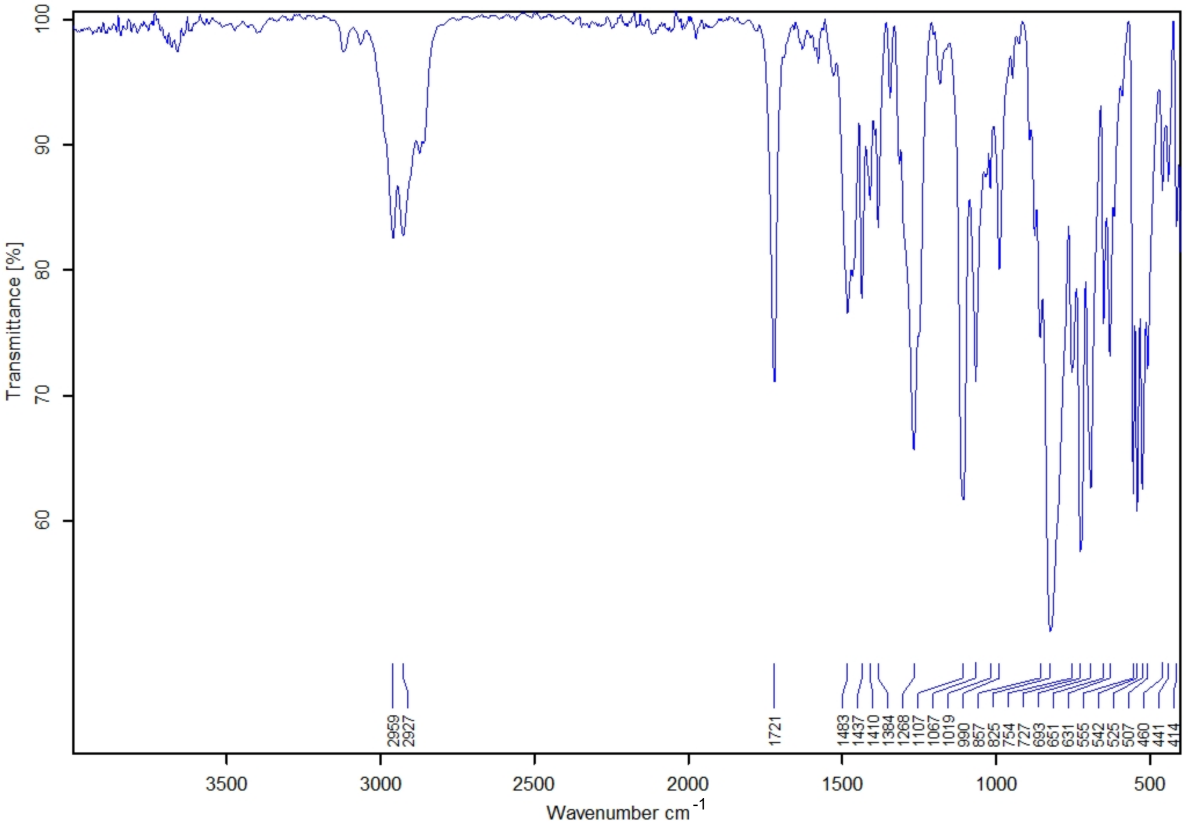

*Figure S38: ATR-IR spectrum of **10**.*

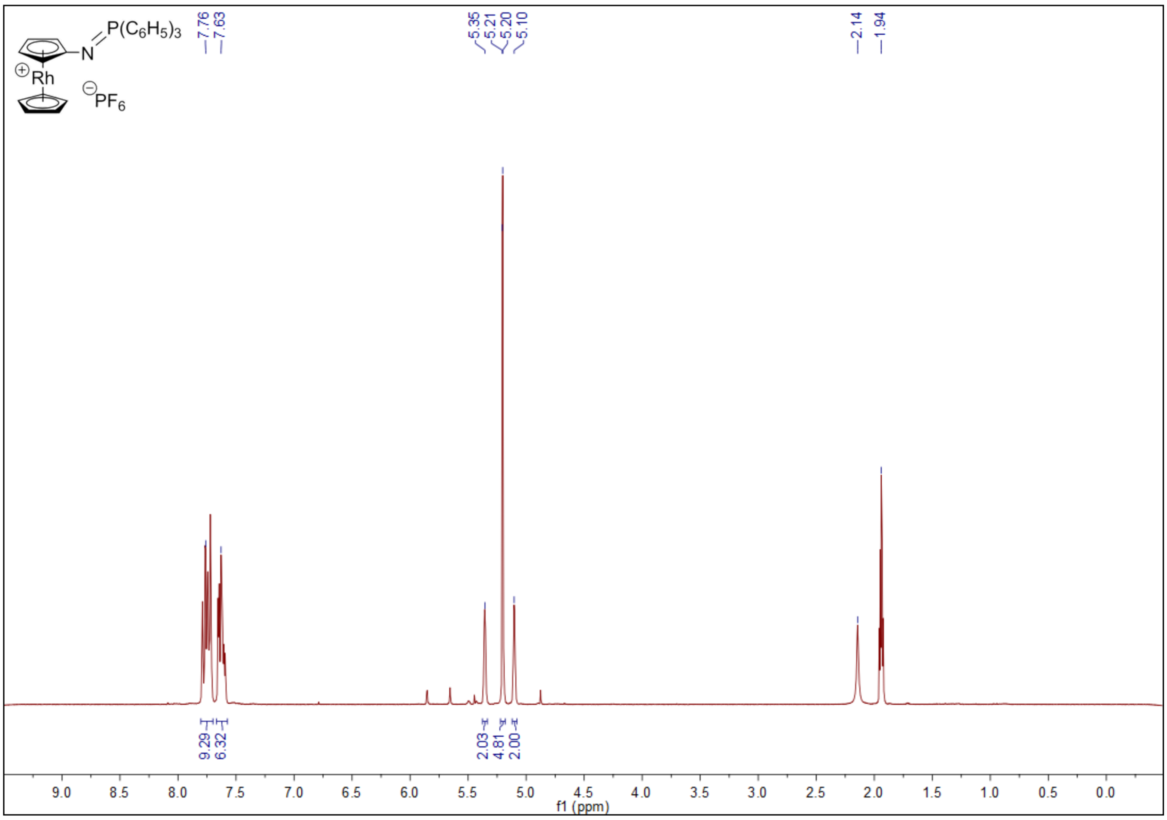

*Figure S39: ^{1}H-NMR of **10** in CD_3CN.*

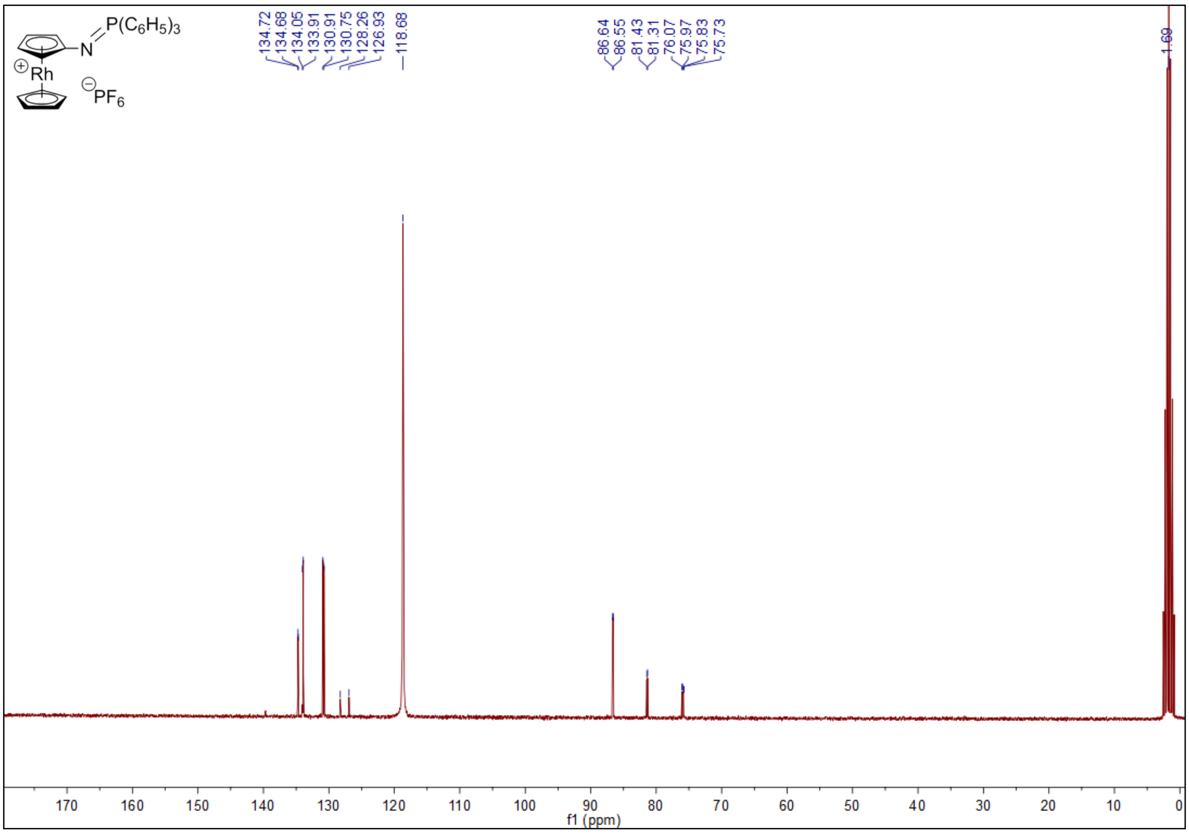

*Figure S40: ^{13}C-NMR of **10** in CD_3CN.*

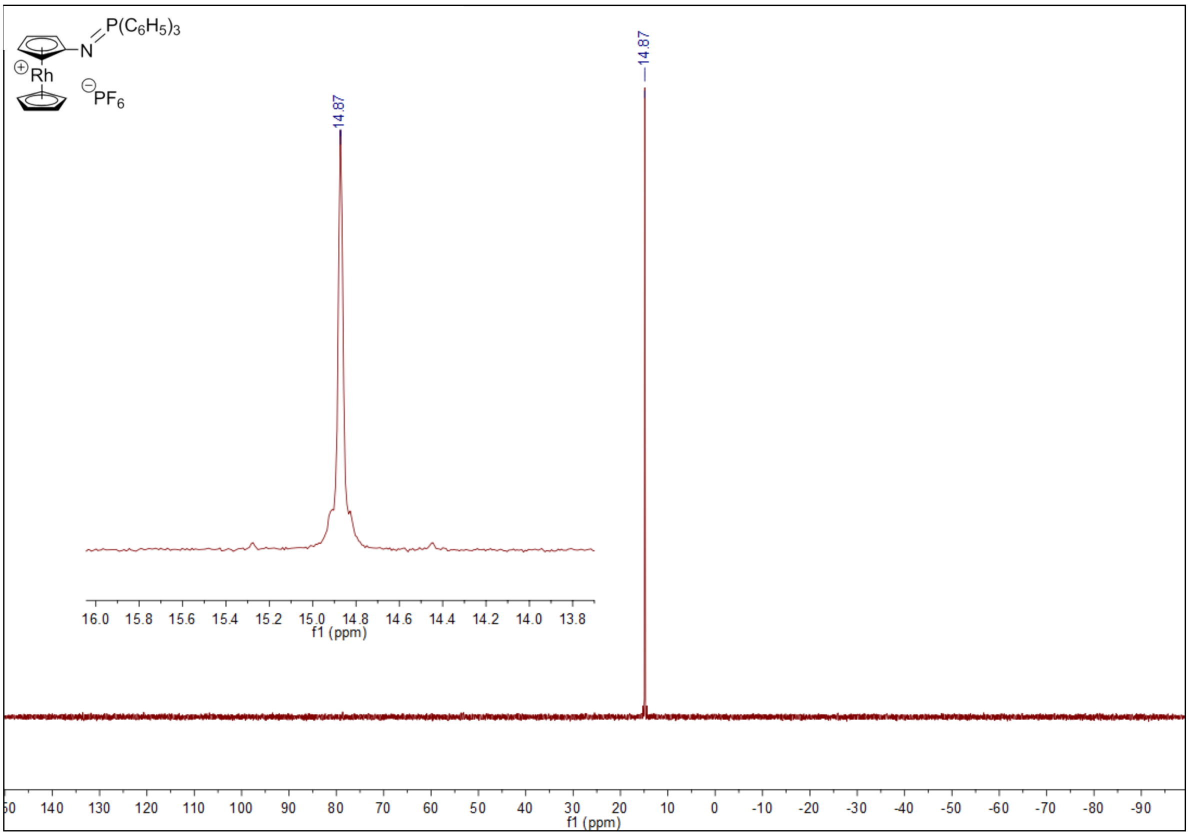

*Figure S41: ^{31}P-NMR of **10** in CD_3CN*

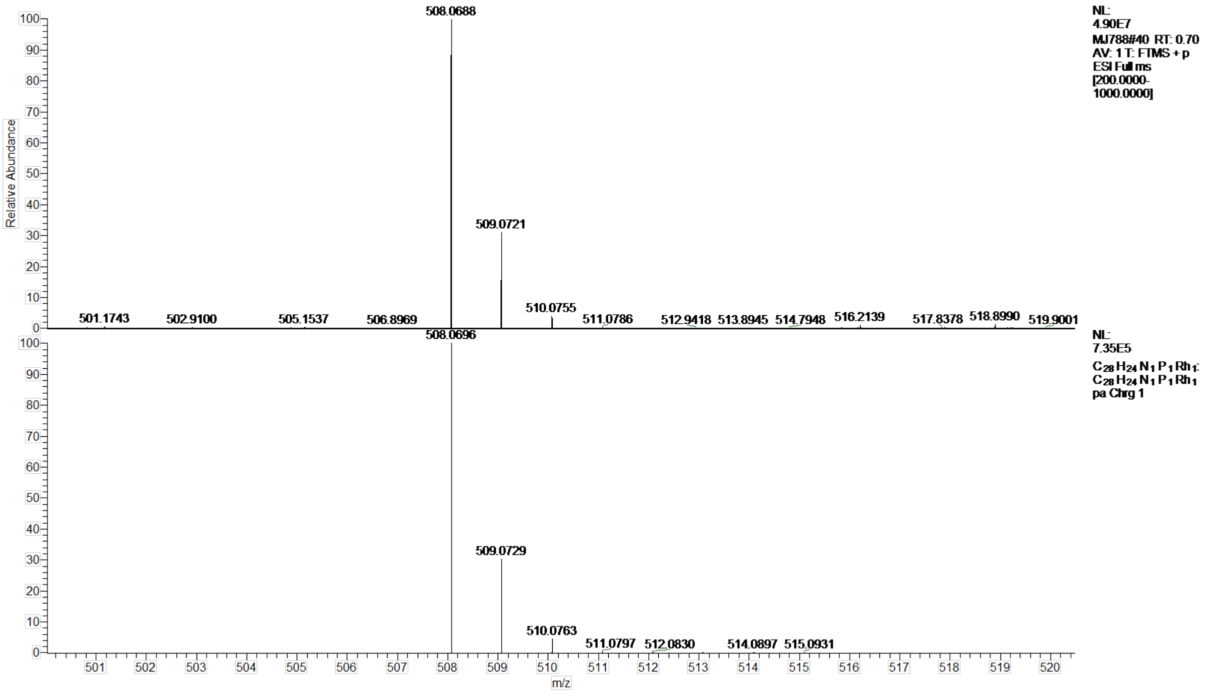

*Figure S42: MS (ESI pos) of **10**.*

2. Additional ^{103}Rh-NMR spectra related to previous compounds[1]

2.1 Rhodocenium hexafluoridophosphate (11)

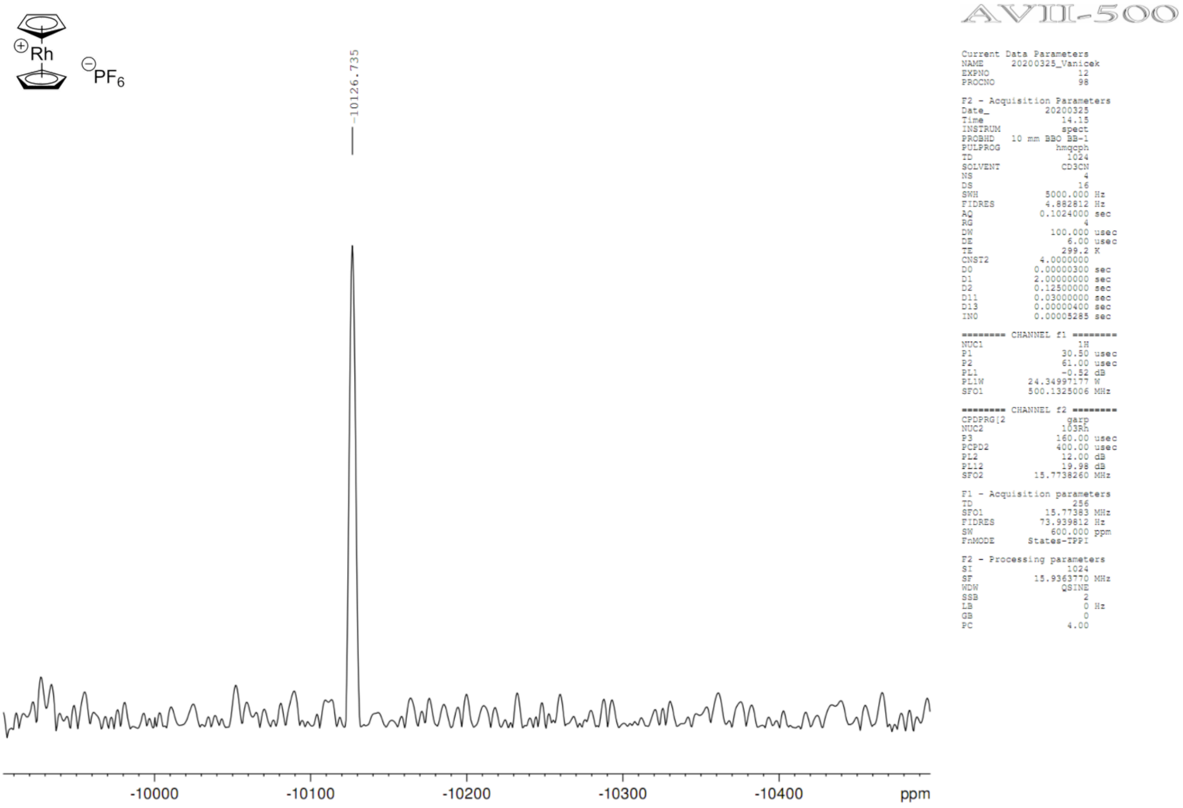

*Figure S43: 1D ^{1}H,^{103}Rh-HMQC-NMR of **11** in CD_3CN.*

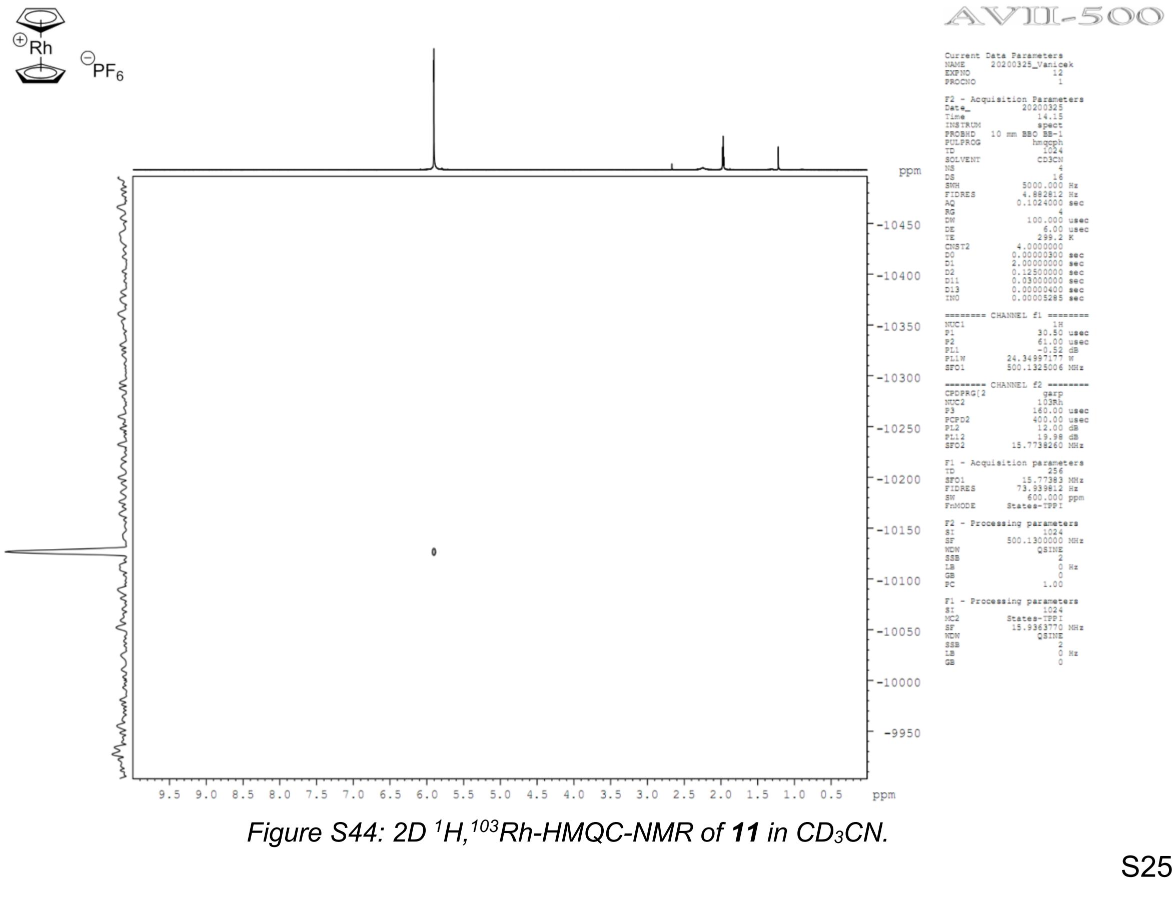

*Figure S44: 2D ^{1}H,^{103}Rh-HMQC-NMR of **11** in CD_3CN.*

2.2 Methylrhodocenium hexafluoridophosphate (12)

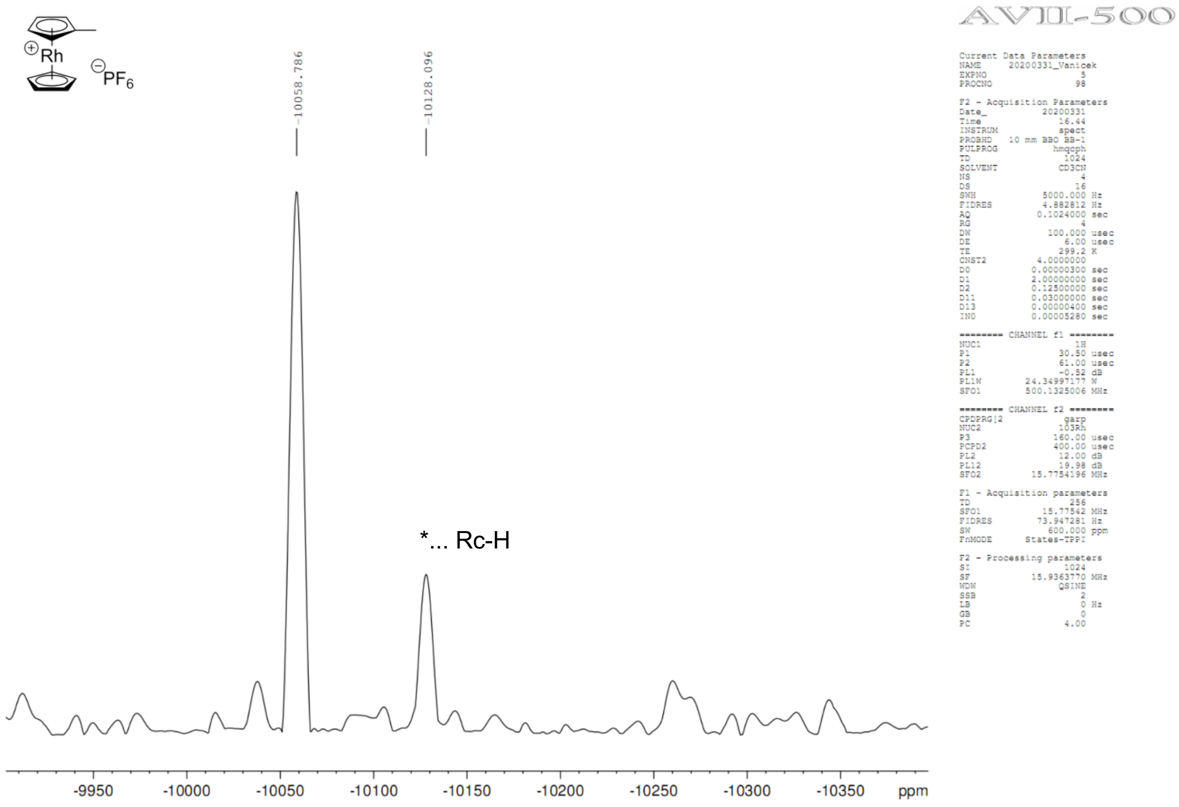

*Figure S45: 1D $^{1}H,^{103}Rh$-HMQC-NMR of **12** in CD_3CN.*

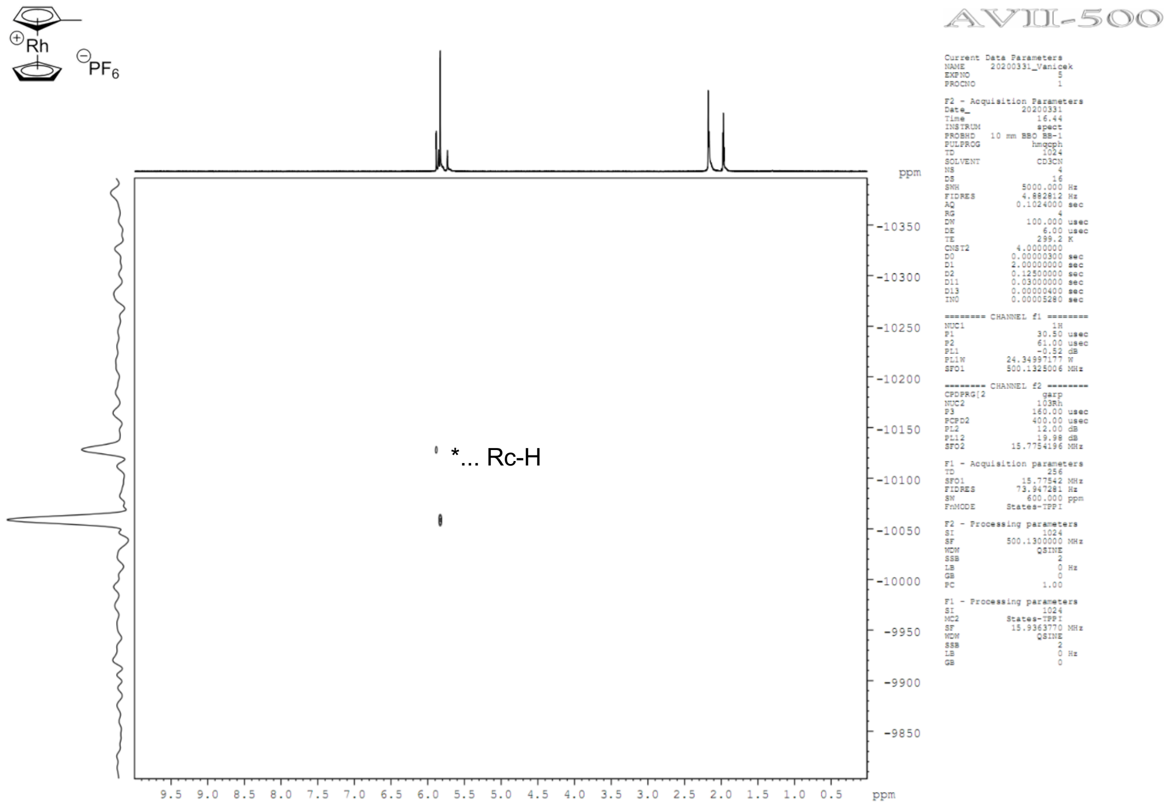

*Figure S46: 2D $^{1}H,^{103}Rh$-HMQC-NMR of **12** in CD_3CN.*

2.3 Rhodococenium carboxylic acid hexafluoridophosphate (13)

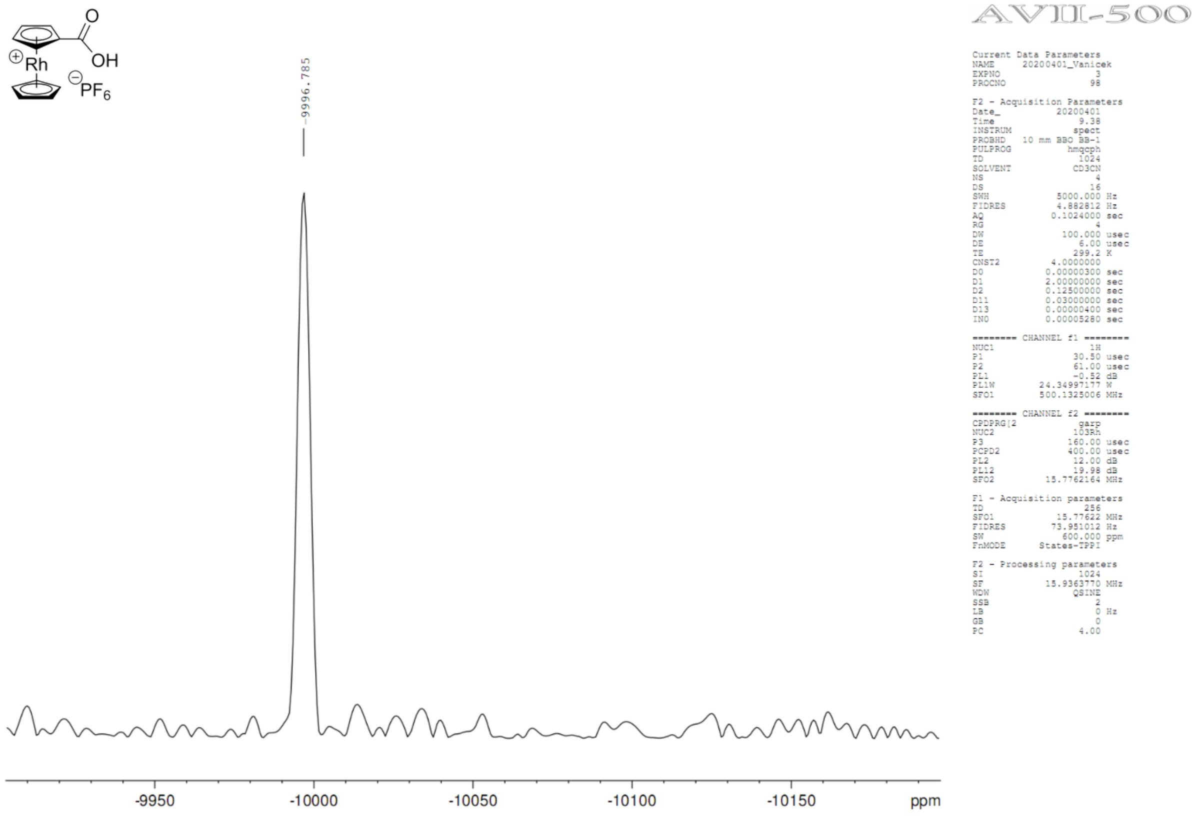

*Figure S47: 1D ^{1}H,^{103}Rh-HMQC-NMR of **13** in CD_3CN.*

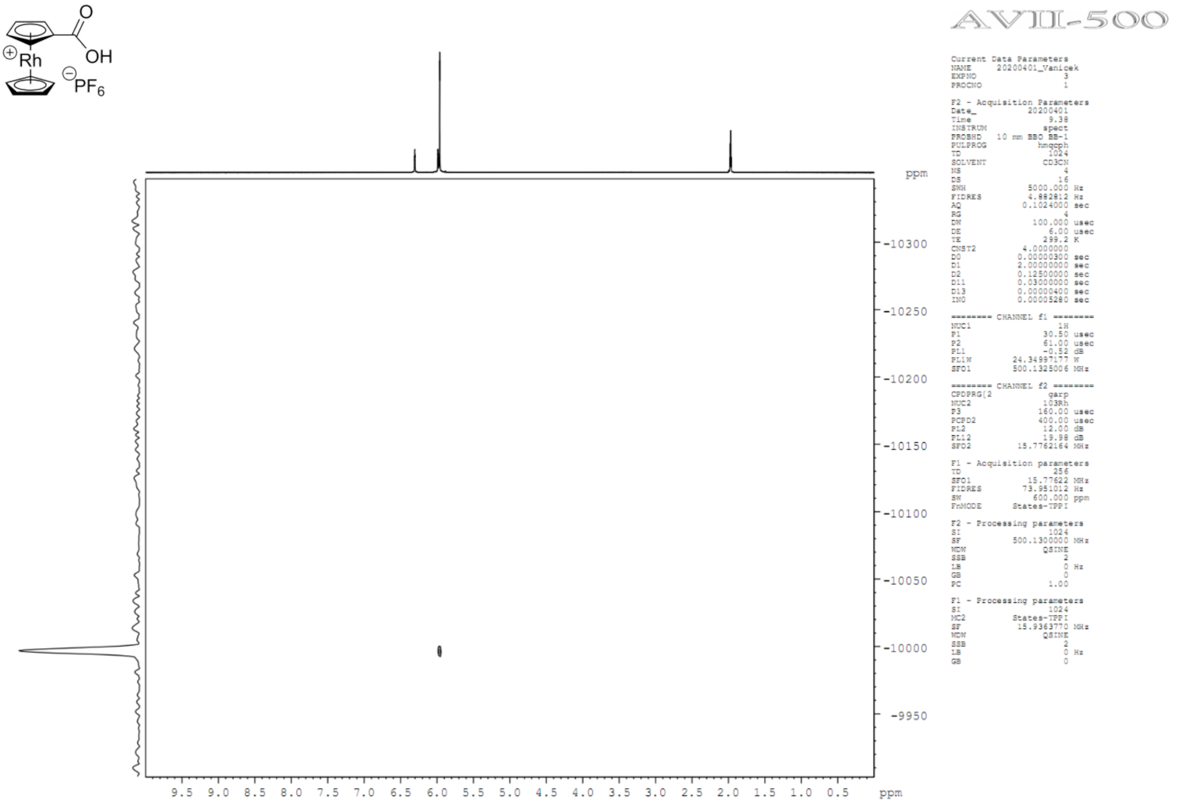

*Figure S48: 2D ^{1}H,^{103}Rh-HMQC-NMR of **13** in CD_3CN.*

2.4 Methyl rhodocenium carboxylate hexafluoridophosphate (14)

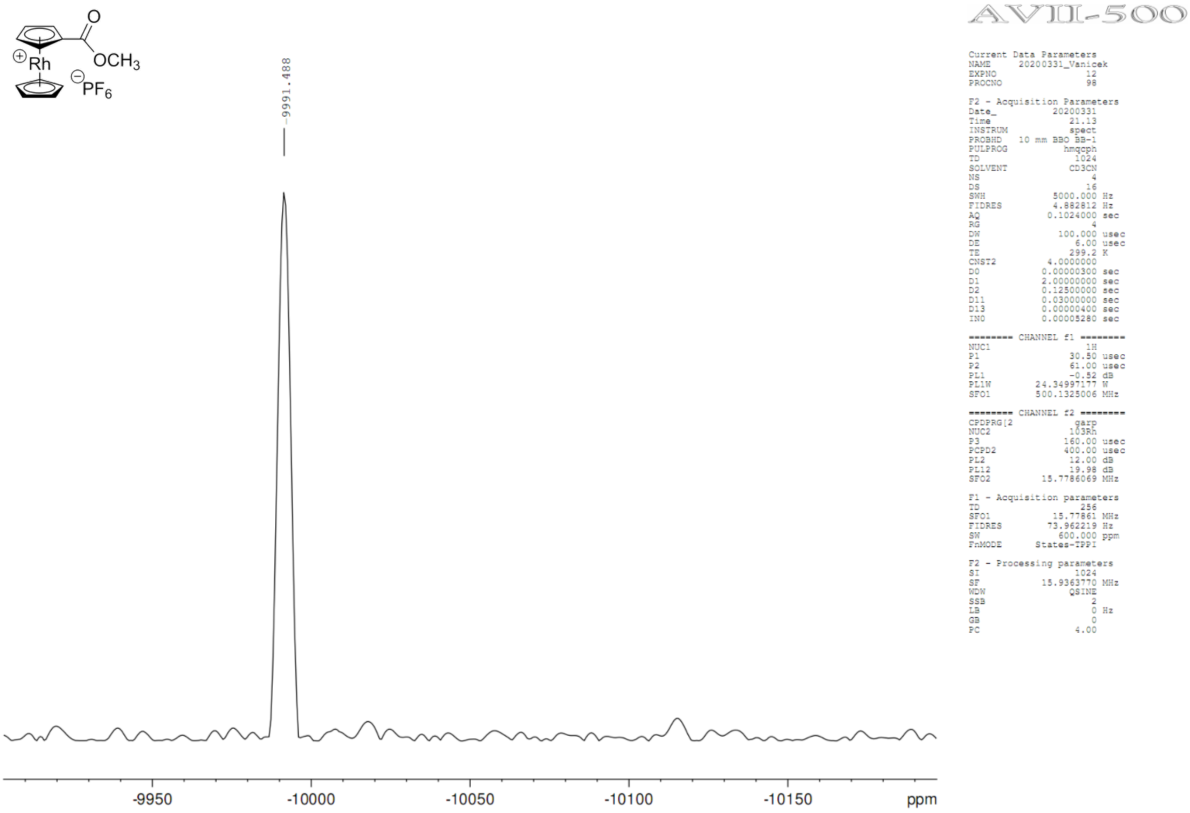

*Figure S49: 1D $^{1}H,^{103}Rh$-HMQC-NMR of **14** in CD_3CN.*

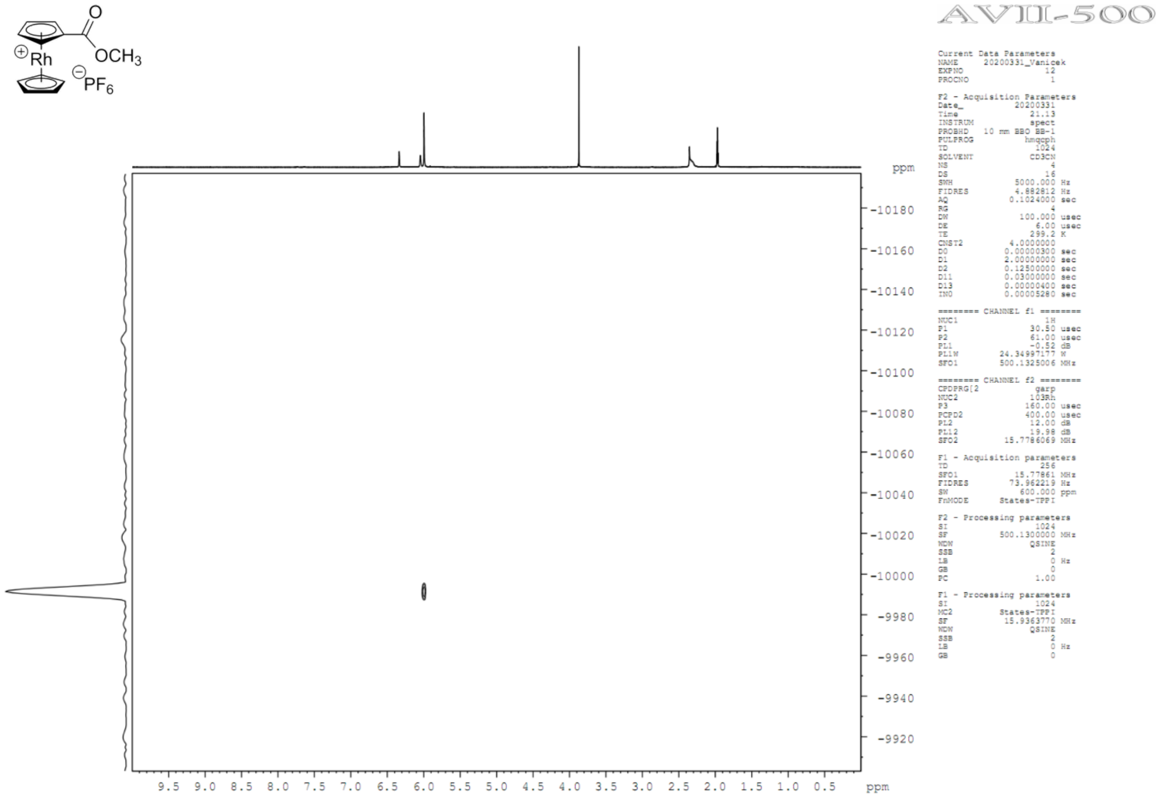

*Figure S50: 2D $^{1}H,^{103}Rh$-HMQC-NMR of **14** in CD_3CN.*

2.5 Rhodocenium carboxylic acid amide hexafluoridophosphate (15)

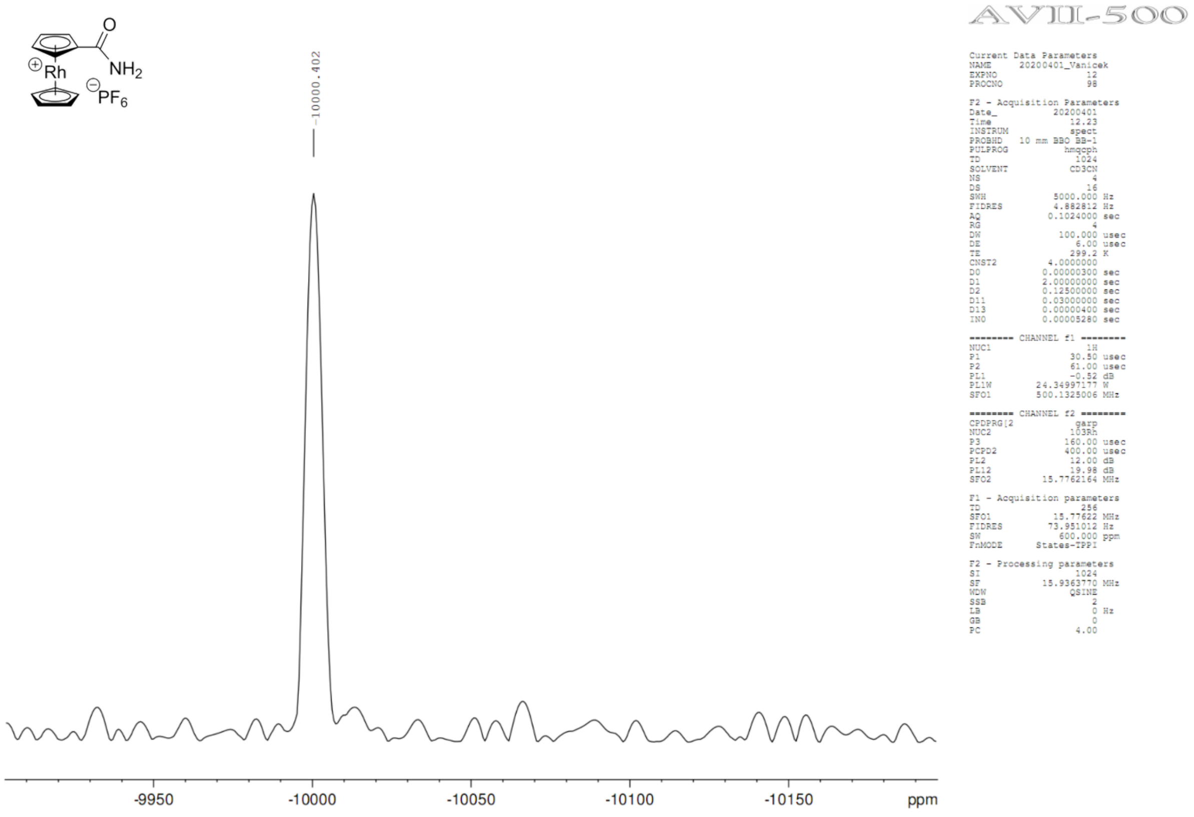

*Figure S51: 1D $^{1}H,^{103}Rh$-HMQC-NMR of **15** in CD_3CN.*

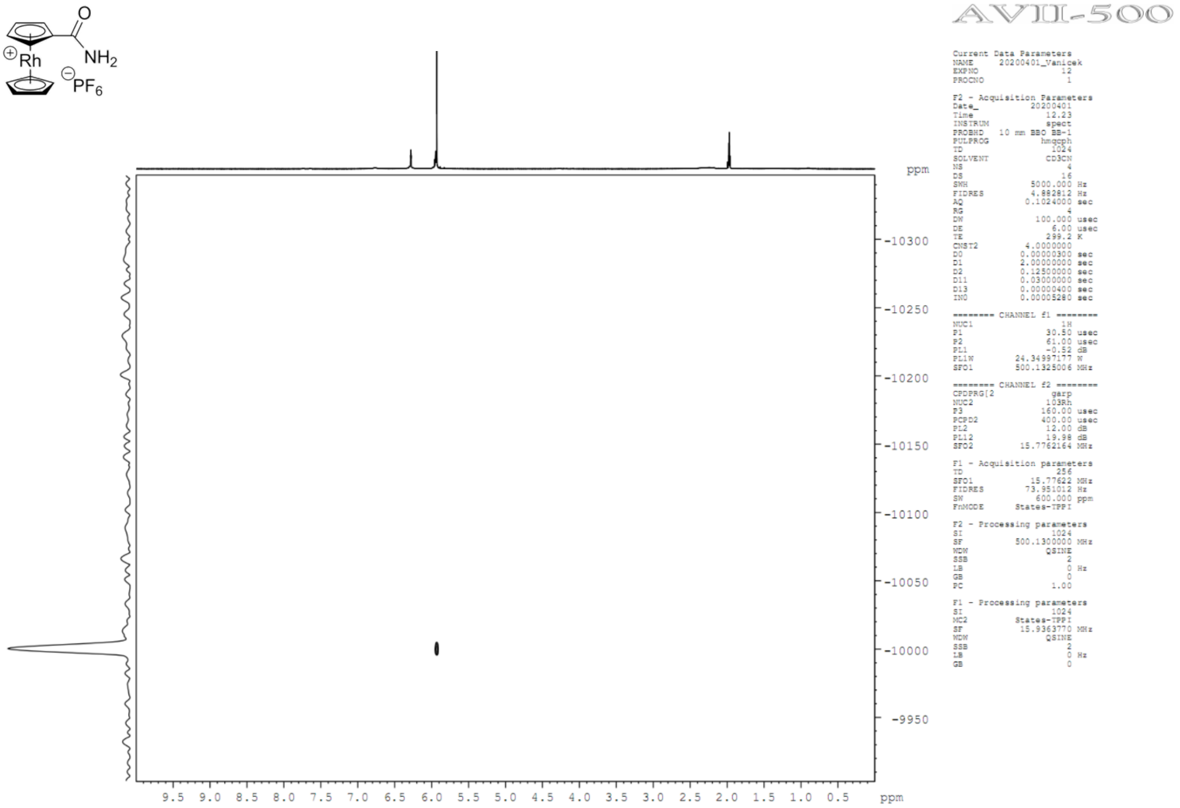

*Figure S52: 2D $^{1}H,^{103}Rh$-HMQC-NMR of **15** in CD_3CN.*

3. Literature

[1] M. Jochriem, L. A. Casper, S. Vanicek, D. Petersen, H. Kopacka, K. Wurst, T. Müller, R. F. Winter, B. Bildstein, *Eur. J. Inorg. Chem.* **2020**, *14*, 1300-1310.
